# Synthesis of Randomly Substituted Anionic Cyclodextrins in Ball Milling

**DOI:** 10.3390/molecules22030485

**Published:** 2017-03-19

**Authors:** László Jicsinszky, Marina Caporaso, Emanuela Calcio Gaudino, Cristina Giovannoli, Giancarlo Cravotto

**Affiliations:** 1 Department of Drug Science and Technology, University of Turin, Via P. Giuria 7, 10125 Turin, Italy; marina.caporaso@unito.it (M.C.); emanuela.calcio@unito.it (E.C.G.); 2 Department of Chemistry, University of Turin, Via P. Giuria 7, 10125 Turin, Italy; cristina.giovannoli@unito.it

**Keywords:** high-energy ball milling, anionic cyclodextrin, solvent-free reactions, random substitution

## Abstract

A number of influencing factors mean that the random substitution of cyclodextrins (CD) in solution is difficult to reproduce. Reaction assembly in mechanochemistry reduces the number of these factors. However, lack of water can improve the reaction outcomes by minimizing the reagent’s hydrolysis. High-energy ball milling is an efficient, green and simple method for one-step reactions and usually reduces degradation and byproduct formation. Anionic CD derivatives have successfully been synthesized in the solid state, using a planetary ball mill. Comparison with solution reactions, the solvent-free conditions strongly reduced the reagent hydrolysis and resulted in products of higher degree of substitution (DS) with more homogeneous DS distribution. The synthesis of anionic CD derivatives can be effectively performed under mechanochemical activation without significant changes to the substitution pattern but the DS distributions were considerably different from the products of solution syntheses.

## 1. Introduction

Cyclodextrins are cyclic oligosaccharides that consist of α (1→4) linked glucopyranosides and have been well known since the last decade of the nineteenth century [1]. Although these macrocycles show dynamic behavior in solution, the average structure of their thermal motion leads to the molecules having a truncated cone shape. Inside this truncated cone, the constituting glucopyranoside units create a less hydrophilic pocket, which allows these macrocycles to from so-called inclusion complexes with geometrically appropriate organic structures.

The spatial arrangements and dissociation properties of the C(6), C(2) and C(3) hydroxyls result in variations in reactivity; in particular, the flexible nature of the primary hydroxyl moiety and steric hindrance can strongly affect the regioselectivity and the yield of conversion. Under classic organic reaction conditions in solution, the preferred position for nucleophilic alkylation is the O(2) position, which may sometimes be considered "reversed" reactivity compared to the regular primary > secondary hydroxyl reactivity order of alcohols [2,3]. After a certain degree of substitution (DS, number of substituents/macrocycle) or at higher temperatures, the primary hydroxyls are easier to substitute. Substitution of C(3) OHs is usually very difficult because of a significant steric hindrance. Inclusion complex formation, solvation and steric effects can considerably influence the composition of the final products, even reverting it to the normal organic reactivity order with the primary hydroxyl reaction becoming dominant. Strong and small electrophile reaction centers, like acylium ions, react unselectively and usually completely with hydroxyl groups at various positions [4].

Ball milling (BM) has long been used for the effective preparation of inclusion complexes [5,6], particulation [7] for better redissolution properties and, quite recently, it has also become a useful and flexible tool for green organic chemists [8,9,10,11], in the form of the more energy efficient high-energy ball milling (HEBM) [12,13,14]. Nowadays, the mechanochemical manipulation of covalent bonds [15] in carbohydrates [16,17], particularly in CD derivatization [12,13,14], is more of a curiosity than routine work. However, the potential of HEBM in various nucleophilic substitution reactions of 6^I^-monotosyl-β-CD has been demonstrated in a previous article [12], and a practical application of this method has been reported recently [14]. It was found that HEBM is highly efficient when good nucleophiles, such as sulfur nucleophiles, are used. Although Menuel et al. successfully synthesized and isolated 2-*O*-monotosyl-α-, -β-, and –γ-CDs in a mixer type mill after flash chromatography, these derivatives currently suffer from a lack of uses. The one-pot transformation of the prepared 2-*O*-monotosyl CDs to CD-derived cyclic macrocycles that contain one 2,3-mannoepoxide residue has opened up a new page in the regioselective derivatization of CDs [13]. This work gave direct evidence that the side reactions that appear to be unavoidable in the classical solution syntheses can be eliminated in the solventless method.

The most common cyclodextrin derivatives on the market, except the randomly methylated ones, are prepared in aqueous medium. The isolation of the reaction products usually requires the removal of huge amounts of water, which is not an energy efficient process. In our paper, we compare the statistical substitution solution reactions of the most popular anionic CD derivatives with those in a ball mill. Performing reactions by mechanochemical activation, particularly HEBM, is less complicated than the majority of solution reactions. HEBM reactions are easily engineered and the scale up causes fewer difficulties than in solution, and, as the laboratory process has been finalized, the kilo-lab or pilot plant scale-up is faster.

The aversion to ball mill assisted organic chemical reactions is principally laid in the relatively high (>100) balls/reagent mass ratio, which makes the work-up size dependent, as we have demonstrated in a previous article [14]. Gram-scale preparations enabled the reagent/balls ratio to be reduced to a reasonable level. Our main goals are to confirm the concept of mechanochemical activation in nucleophilic reactions involving oxygen and introduce a green alternative to CD derivatizations.

## 2. Results and Discussion

**Scheme 1:**
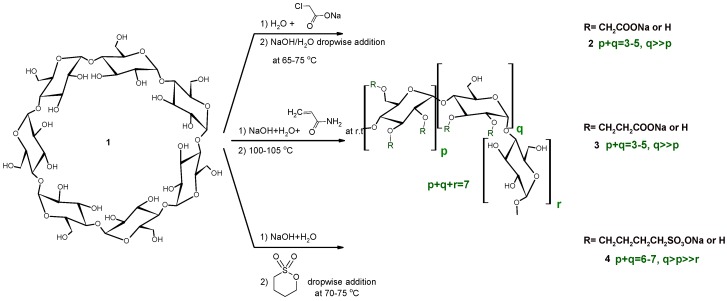
Synthesis of anionic βCD derivatives in solution.

The three different anionic CD derivatives were prepared in different ways in solution, as seen in Scheme 1. While the carboxymethylated CDs (CMCDs) are prepared via the addition of sodium hydroxide to a solution of the parent CD and chloroacetic acid sodium salt (ClAcONa) at elevated temperature, the reagents in the carboxyethylated CDs (CECDs) are weighed together and reacted at room temperature, while the sulfobutylated CDs (SBCDs) are prepared by the addition of 1,4-butanesultone to a very concentrated solution of CD and NaOH at elevated temperature. Despite the very different reaction conditions, the substitution patterns show similarity; secondary hydroxyls are dominantly substituted. Additionally, the DS distribution of the products shows a similar profile, as a Gaussian-like DS distribution. Solution reactions are carried out in water, which causes hydrolysis as a side reaction. Considerable primary rim substitution appears only at higher DS (SBβCD). Although similar results were generally found across the mechanochemical syntheses, some minor differences were found in the substitution patterns. Owing to the lower flexibility of the molecules and lack of a solvation effect, the solid state ball milling reactions usually have a four-member transition state and nucleophilic substitution “SN2-type” reactions are dominant [18].

**Scheme 2:**
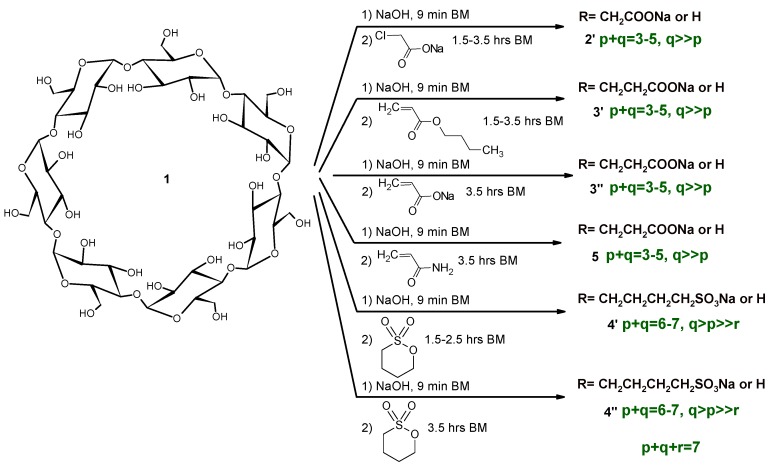
Synthesis of anionic βCD derivatives with high-energy ball milling (HEBM).

Mechanochemistry usually uses reagent molar ratios that are different to the solution reactions. It is also true that their relative ratios can be controlled via the addition method, despite the overall molar ratios being identical across the various solution reactions. In order to compare the products of the green and classic reaction conditions, identical molar ratios were chosen in the majority of reactions. The summary of our reaction conditions can be seen in Scheme 2.

Reactions were carried out in two steps in order to reduce the potential for reagent hydrolysis. In the first, shorter period, the preparation of the βCD sodium salt was carried out before adding the reagents. It was found that the formation of the βCD sodium salt is somehow exothermic, despite the temperature not being considerably higher (<40 °C) than without reagents [12]. The temperature course of the reaction showed the same saturation behavior as before [3,12] and remained below 100 °C in all cases.

The scarce (couple of tenth-percent) linear oligosaccharide residues in the starting material always produced coloration and the aqueous solutions changed from light to dark yellow after the HEBM reactions. This effect is heavily temperature dependent and more evident in solution and sometimes—particularly in the case of CE and SB syntheses when heating at ≈100 °C was necessary —the reaction mixture turned red before work-up. In ball milling, similar coloration had been found, but this usually was rather dark yellow than red. These impurities could be easily removed with active carbon treatment in both methods. Yields were calculated from the DS, which is, in turn, determined from the NMR spectra of isolated products.

### 2.1. Carboxymethylation (Compounds ***2*** and ***2*’**)

The synthesis of carboxymethylated cyclodextrins (CMCDs) in solution can be carried out in two different ways: (a) dropwise addition of the ClAcONa to a basic solution of the CD and (b) the reverse, addition of a base to the solution of CD and reagent. Although CD solubility is considerably higher in strong basic solutions, thanks to ionization, the high concentration of the base results in the decomposition of ClAcONa. The hydrolysis of ClAcONa reduces the effectiveness of the reaction. This side reaction is pH and temperature dependent, while the lower nucleophilicity of the oxygen means that higher temperatures, compared to nitrogen and sulfur nucleophiles, are needed to achieve successful substitution. The reverse reaction also suffers from some drawbacks, namely the low solubility of βCD in water, the relatively long residence time of ClAcONa at elevated temperatures and the possible formation of diglycolic acid. Chloroacetic acid has been found to be unstable in aqueous solutions, even in neutral or acidic conditions [19,20,21]. Although the pH of the solution is controlled by the addition rate of the base, the instability of the reagent, even at low pH values, can reduce the added base, which results in a lower DS for the product. Because the CMCD derivatives are randomly substituted CD derivatives, the laboratory/production site effect makes the exact reproduction difficult. The dependency on the in-house personnel in the statistical substitution of CDs can be reduced but not totally eliminated.

As expected, some residual diglycolic acid (<0.5%) was found after workup (compound **2**, item 1 in Table 1). Similar by-products were not expected after the HEBM reaction, as not only is the hydrolysis restricted, but further reaction with another ClAcONa is practically negligible. In our previous publications, the reaction times were set to the 90–120 min range; however, we used the shorter period (1.5 h) in our first reactions here in order to prove the concept. In the ClAcONa case, this period appeared to be a little short because, although the yield was high, the product contained an unacceptable level of ClAcONa and/or glycolic acid (>2.5%), while the DS was low but not essentially lower (item 2 in Table 1). It was assumed that, because the product contained ClAcONa—present in too high a quantity for the ion-exchanger to remove—a longer milling time would not only increase the DS of the CD derivative, but also reduced the unreacted ClAcONa derived impurities in the product. A one-hour longer reaction resulted in a slightly higher DS, which became almost identical to that of the solution reaction, and no ClAcONa derived by-product was found in the NMR spectrum (item 3 in Table 1).

Natural CDs contain a considerable amount of water (10%–14%, depending on the CD-version) and are therefore sometimes called CD-hydrates in some fine chemical catalogues. While it was an obvious thought to test the reaction with dried βCD, it was not practically reasonable. This is because drying is an expensive process and dried CDs are difficult to weigh, very electrostatic and hygroscopic materials. The unfavorable properties of the anhydrous form are more inconvenient on a larger scale as well. Additionally, air-dry βCD contains ≈10 mole water, and this amount was commensurable with the molar amounts formed upon sodium salt formation (≈6 mole), with some coming from the reagents and air upon manipulation. Not only was the yield surprisingly found to be lower (item 4 in Table 1) in the HEBM reaction as compared to the hydrate case (item 3 in Table 1), but the noticeable amount of the diglycolic acid by-product highlighted the existence of higher-than-expected hydrolysis and the concurrent substitution of the formed glycolic acid. The DS of the product was in a similar range as found previously, despite the lower yield. A plausible explanation for this may be that, because of the solid state, the water formed upon salt formation resided very close to the reaction centers. This proximity can result in the hydrolysis of the reagent, and a second ClAcONa might also react with the hydrolytic compound due to the slow solid-state diffusion. The reduced reagent amount consequently resulted in lower yields but does not explain the similar DS range. The low importance of the usage of dried βCD in the preparation of CMCDs meant that further study into the reactions of dried CD in the carboxymethylation was stopped.

**Figure 1:**
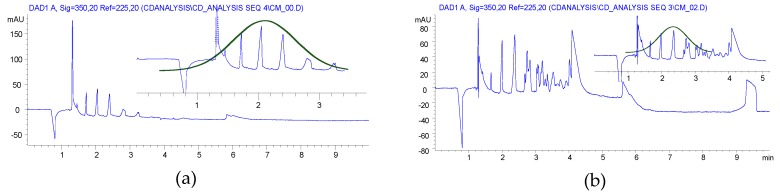
Capillary electropherograms of carboxymethylated βCD (**a**) synthesized in solution (compound 2, item 1 in Table 1); (**b**) synthesized in ball mill (compound **2′**, item 3 in Table 1).

CE analyses of the HEBM reaction showed similar charge distribution of the products, which is reflected in the substitution distribution pattern. Significant differences were found between the solution reaction and BM reactions, as it is seen in Figure 1. While the solution reaction resulted in a Gaussian-like distribution of the different DS, the HEBM reactions showed a balanced distribution of the variously charged CD derivatives. Although the 1 h longer reaction time also resulted in a Gaussian-like DS distribution with a slightly higher maximum (item 3 in Table 1) and the highly substituted components also appeared. It was also found that the removal of the ClAcONa related impurities was less effective than in the solution case. The longer milling time had an effect on the charge (DS) distribution (item 2 vs. item 3 in Table 1), but, using dried βCD as starting material, the DS distribution showed less similarity to the solution case (item 2 vs. item 4 in Table 1) and it was also shifted to the higher substitution range (see Figure S10 in Supplementary Materials). Although the CE is less sensitive to the isomeric differences of CD derivatives the wider and multiple peaks in the higher charged region show the formation of isomers of identical DS. From the NMR studies, the dominant, almost exclusive O(2) substitution can be deduced, and the multiple but relatively sharp peaks of the higher substituted CMβCDs also confirm this assumption. The lack of solvation effects and a more rigid structure could result in less steric effects and, predominantly, the most reactive and most accessible O(2) can react.

Although NMR spectra of the compounds, prepared by both methods, showed similar substitution patterns in terms of the substitution site, the CE experiments showed a more balanced distribution of the differently substituted macrocycles. The more efficient utilization of the reagent suggests that the variation of the ClAcONa/CD ratio can result in identical DS of CMCD with similar charge distribution to the solution synthesis. Further optimization should be done in order to find optimal milling time, reagent ratio, and a still economic, but more effective work-up.

### 2.2. Carboxyethylation (Compounds ***3*** and ***3*′**)

The preparation of CECDs in solution is better reproducible when the Michael reaction is applied. All of the reaction components are weighed together, practically simultaneously, which takes out the majority of production site effects. In the first step of the reaction, the acrylamide is added to the CDs and then the hydrolysis of the amide gives the CEβCD (compound **3**, item 8 in Table 1) in good yields. Although the amides are more resistant to hydrolysis in aqueous solution, the longer reaction time can lead to this very same reaction. The acrylic acid is a much less suitable Michael acceptor and hydrolysis of the reagent can reduce the yield and/or DS of the product. While the low reaction temperature in the first step is a real problem, adjusting the appropriate acrylamide/CD ratio can easily resolve the issue. In the Michael reaction, the base works as catalyst and the low amount has a lesser effect on the DS of the CD derivative. The hydrolysis of the CD-attached amide also reduces the base amount, but it does not influence possible impurities because the final product is the acid. A major drawback of the solution method is that the CD-amide intermediate is difficult to isolate because the Michael reaction results in random substitution and the hydrolysis of the product is also statistical. After the hydrolysis of the CD-amide, the similar work-up, as in the CMCD, was not enough effective. The less hydrophilic acrylic compounds than acetic acid derivatives, without changing the substitution patterns, are more difficult to remove with anion-exchangers, and dialysis was necessary to reach acceptable purity levels (item 8 in Table 1). Although dialysis is a convenient method to remove inorganic salts and small molecular weight impurities, not only the reproducibility but also the scale up and environmental issues restrict its use in large scale organic syntheses. Infra-Red (IR) studies of the product confirmed that the product does not contain a noticeable amount of amide, indicating that the hydrolysis was practically complete (see Supplementary Materials, Figures S24–S26).

The direct synthesis of CEβCD using the HEBM method is more difficult than it seems. Since the halogen-propionic acids are very reactive, both hydrolysis and elimination can take place in basic solution. Nucleophilic conjugate addition, like the Michael reaction, is a convenient tool that can eliminate some reagent-derived by-products. The Michael reaction requires a loosened double bond that can be found in the acrylic acid derivatives, but that are not so prevalent in the acrylate salts. The methyl and ethyl acrylates have boiling points that are too low to carry out the reaction in standard ball mill equipment, they have an unpleasant odor, and their vapors are usually toxic. As it could be seen in the CM cases, the hydrolysis of OH sensitive reagents in the solid state is not completely eliminated, despite being suppressed. Considering the technical difficulties, *n*-butyl acrylate was used as a reagent with a high enough boiling point to be safe, more resistant against hydrolysis and lipophilic enough to make the work-up effective. The longer, 2.5 h milling period was decided upon based on previous experience with CM reactions. After ball milling, the reaction mixtures were soluble in acetone and EtOH, while the added water was able to hydrolyze the esters giving the products (compound **3′**, item 5 and 6 in Table 1). Low percentages of the partially hydrolyzed CD derivative and residual 1-butanol were also found in the isolated solids. The obtained low yields highlighted the room for more optimization. In order to reduce ester hydrolysis, the reaction was repeated with dried βCD as well. In this case, a reduced yield was also found like in the CM reactions. Although the hydrolytic product, acrylic acid sodium salt, is less reactive than the ester, we could not find a reasonable explanation for the significantly lower yield from dried βCD.

The possibly incompletely hydrolyzed CD-esters remained in solution and further hydrolysis after solvent removal was still incomplete. TLC and NMR experiments showed that the higher DS fragments of the βCD derivative remained in solution. Aqueous hydrolysis could have been completed with the use of more base and higher temperatures, but that additional effort makes the work-up procedures more complicated and so they were not applied. The substitution pattern and DS were in the acceptance range of the solution phase reaction, but the inherited impurities from the incomplete hydrolysis somewhat modulate the positive picture.

**Figure 2:**
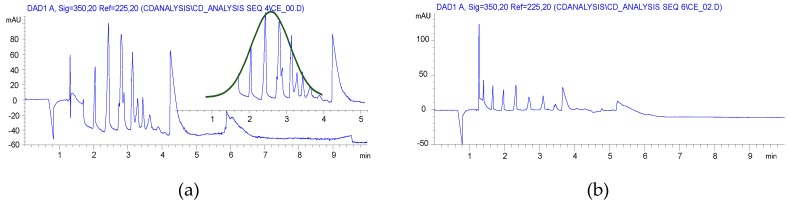
Capillary electropherograms of carboxyethylated βCD (**a**) synthesized in solution (compound **3**, item 8 in Table 1); (**b**) synthesized in ball mill (compound **3′**, item 6 in Table 1).

CE analysis of the solution preparation showed also a Gaussian-like envelope like the carboxymethyl case (Figure 2a), while the ball mill reaction gave a more balanced charge distribution (Figure 2b).

Acrylic salts are less common Michael acceptors and our attempts to use sodium acrylate for the preparation of carboxyethylated cyclodextrins (CECDs) would be a great achievement in the synthesis. Not only would the side reaction be eliminated, but the work-up could also simplify the CM case. Considering the lower reactivity, the reaction time was further increased (item 7 in Table 1). The NMR spectra revealed that a considerable amount of acrylic acid remained in the product, but the yield seemed to be good. Unfortunately, big differences in the substitution patterns, compared with both the solution and *n*-butyl acrylate reactions, were found although the DS were in the acceptation range. It was also found that a large part of the used βCD was unreacted in this reaction. It was found that the acrylate reacts almost exclusively with the O(2) of the glucopyranoside units.

**Figure 3:**
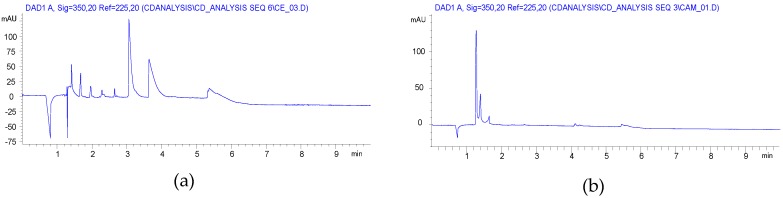
Capillary electropherograms of carboxyethylated βCD (**a**) synthesized from sodium acrylate (compound **3′′**, item 7 in Table 1); and (**b**) carboxyethyl residue in carbamoylethyl βCD (compound **5**, item 9 in Table 1).

The CE analysis confirmed that the relatively high acrylic acid related impurities and the charge distribution were different, not only from the solution reaction but also from the HEBM reactions using acrylic ester as reagent. The lower reactivity of the acrylate salt is seen also by the dominant of low DS products (Figure 3a).

### 2.3. Carbamoylethylation (Compound ***5***)

The isolation of the CD-amide was made a target with the idea of exploiting the lower rate of hydrolysis in the HEBM reactions. This derivative is a somewhat more flexible CD derivative and the hydrolysis of the product leads to the previously targeted CECDs; however, the amide can be reduced to the amine under suitable conditions, which can widen the easy-to-prepare amino-CD repertoire.

At first, we extended the reaction time to 5.5 h (item 9 in Table 1), and used a periodical analysis of the reaction mixture of the βCD/NaOH/acrylamide system to achieve a clear picture of the addition/hydrolysis ratio of this reaction. At somewhere between 3 and 4 h milling, the residual acrylic derivative became minimal. After work-up, the NMR spectra showed the presence of acid residues on the CD. The acrylic residues are around the acceptance level. While the longer reaction time resulted in higher DS, the approx. 20% amide was hydrolyzed, as seen in Figure 3b, and the yield was somewhat lower than at the shorter, 3.5 h milling time. The DS 3.5 h reaction (item 10 in Table 1) was closer both to the solution and the acrylic ester HEBM reactions, but the substitution pattern seems to be a little different from the solution reaction, where a small portion of primary substitution can be assumed. The comparison of the IR spectra of the isolated products (items 9 and 10 in Table 1) showed the presence of an acidic moiety in compound **5** of longer HEBM (item 9 in Table 1) and the lack of it in the product of shorter milling time (items 10 in Table 1) (see Supplementary Materials, Figures S30, S31, S35, and S36).

The CD-amide was isolated from a milled βCD/NaOH/acrylamide mixture and very similar DS and substitution patterns to the classic reaction were achieved. A shorter milling time reduced the amide hydrolysis which allows—by good reaction engineering—to synthesize the more flexible carbamoylethyl intermediate.

### 2.4. Sulfobutylation (Compounds ***4*** and ***4*′**)

The sulfobutylation reaction is the most difficult of the reactions studied. This is not due to the difficulties of the reaction, but rather the patent policy of some countries. While the originally patented and later FDA approved product was a poorly characterized compound, despite its being state-of-the-art at the time of filing, more and more restraints were later brought in to protect the intellectual property. The design of a workaround process in solution is difficult but not impossible. An exact copy of a randomly substituted complex derivative with an essentially different method seems to be impossible, while the higher demand for a successful drug carrier requires improved technologies. In the classic reaction, the DS and substitution patterns can be well controlled by the addition of the reagent. In this case, the low aqueous solubility of the BS restricts the applicable variations in the implementation of the reaction. The solution reaction resulted in a good yield of targeted product with—in terms of analysis—acceptable purity (item 11 in Table 1).

HEBM reactions have the advantage of more standardized reaction conditions and easier handling of the toxic reagent, 1,4-butanesultone. At the same time, the complete hydrolysis of the unused reagent is essential because it is limited to the ppm level for pharmaceutical applications. This task is not obvious even in solution because the complex formation between the CD and BS protects the unreacted BS from hydrolysis. In aqueous solutions, hydrolysis can be accelerated via the addition of ethanol which decomposes the formed BS/SBβCD, but this is more difficult under HEBM conditions as water is usually necessary not only for complex formation with CDs, but also for the release of the entrapped compounds. In solution, the hydrolysis of the residual BS is a part of the reaction and its reduction to the acceptable level belongs to the work-up procedure [22]. Owing to the technical differences between the classic and mechanochemistry methods, the decomposition of the BS is more restricted. In order to minimize the residual BS content, the effect of milling time and NaOH/BS ratio was also studied. The usual 90 min milling resulted in relatively high BS content, ≈2.5 mole by NMR, with practically identical amounts of the NaOH as the solution reaction (item 12 in Table 1). The DS was considerably lower, around 3, and the product contained some unsubstituted βCD as well. Extending the reaction time to 2.5 h (item 13 in Table 1) almost halved the residual BS content and the DS was brought closer to the DS of the solution reaction product. The hydrolysis product, 4-hydroxy-butanesulfonic acid, could be also identified in the NMR spectrum and obviously in the capillary electropherograms (≈2.9 min migration time). The higher DS resulted in somehow higher yield, but the reaction mixture still contained unsubstituted βCD on TLC.

Double the amount of NaOH practically eliminated the unsubstituted βCD (item 14 in Table 1), while the BS content was reduced to about 1/5 of the previous product (item 14 in Table 1). The DS of the product did not change essentially. Substituting the air-dry βCD with the dried one (item 15 in Table 1) led to the residual BS content being further reduced to 1/3 of the previous reaction (item 14 in Table 1), but the high 4-hydroxy-butanesulfonic acid content could not be removed by the applied method. Repetition of the purification was not successful and the product was further contaminated with the resin decompositions, which seems to be good guests for the CD. Owing to the highly UV active impurities, the capillary electropherogram became useless. The DS of this product was a little higher than targeted, signifying a better utilization of the reagent. The lower BS content demonstrated also that unreacted BS is a good guest for the βCD and that complex formation prevents its hydrolysis.

In solution, the correct DS and substitution patterns can be controlled by varying the addition of the base and reagent, so that a simulation of the addition was also carried out. The BS was divided into two equal parts (item 16 in Table 1) and milled for 1.5–1.5 h after its addition. Although the yield and DS increased in comparison with the first HEBM reaction (item 12 in Table 1), the residual BS content was almost identical to the lower NaOH ratio and 2.5 h milling (item 13 in Table 1). The DS distribution smoothly shifted to the highly charged derivatives.

A further one-hour extension of the reaction time (item 17 in Table 1) increased the yield, while the DS did not change essentially and remained around 7 as compared to the 2.5 h milling time with identical reagent ratio (item 14 in Table 1). Although the residual BS content decreased considerably in comparison with the identical reaction after only 2.5 h milling, it was a little lower than in the dried βCD case (item 15 in Table 1). The substitution pattern changed a little and became closer to the solution reaction by means of the appearance of the primary substitution signal in the NMR spectrum, but this similarity was not supported by the capillary electrophoresis.

**Figure 4:**
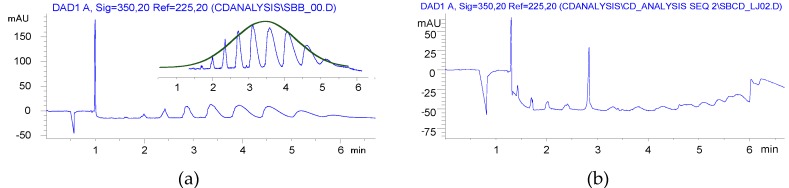
Capillary electropherograms of sulfobutylated βCD (**a**) synthesized in solution (compound **4**, item 7 in Table 1); (**b**) synthesized in ball mill (compound **4′**, item 13 in Table 1).

Furthermore, a Gaussian-like envelope was found in the solution reaction by CE analysis, while the balanced distribution of products of various DS was similar to the previous derivatives produced in HEBM reactions (Figure 4).

Although BS was used at a slightly lower ratio in HEBM than in solution, its utilization was somehow resulted in higher DS than the solution reaction. The yields were close to the solution method, but the residual BS content could not be reduced to ppm levels.

### 2.5. Other Reactions (Compounds ***6*** and ***7***)

Ethylated CDs can be used for the controlled release of various drugs. However, the ethylation of cyclodextrins in solution is more difficult than their methylation, (2-hydroxy)propylation or the reactions described above. Not only are the usable reagents fewer in number, but the higher lipophilicity of the product means that it is not possible to use pure aqueous solutions. In fact, the prices of ethylated CDs mirror the synthetic difficulties and a more effective synthetic route can accelerate the spread of this version of CD derivatives.

Reagent volatility is a serious factor in HEBM reactions. Diethyl sulfate is a suitable reagent for *O*-ethylations, and its lower volatility makes this reagent more equipment compatible and safer. According to previous experience, it was decided to use a long milling time. Only traces of product were isolated after 3.5 h of HEBM. Extending the reaction time to 6 h resulted in no improvements. It was found that the low conversion of βCD to an ethylated derivative is restricted by at least three factors: (i) the reaction centers are poorly accessible for the bulky reagent; (ii) the liquid phase not only resulted in lower reaction temperatures, by at least 20 °C, but also solubilized the CD; and (iii) under the applied conditions, the hydrolysis of the diethyl sulfate is faster than the substation of the CD hydroxyls. The solubilization of the CD sodium salt accelerates its dissociation and the side reactions became dominant. The hydrolysis product of the diethyl sulfate, EtOH, was easily converted to the diethyl ether, which made the reaction hazardous.

Further substitution of a symmetrically methylated βCD reduces its melting point and this property made them suitable additives for capillary GC. The more sterically hindered C(3) hydroxyls have difficulty reacting and a very strong base, such as NaH, is necessary. An additional difficulty for these reactions is that exhaustive substitution usually requires multiple reactions. Heptakis(2,6-di-*O*-methyl-3-*O*-*n*-pentyl)-βCD has been prepared in solution [23] and this synthesis was attempted by HEBM as it provides a significant challenge.

No CD substitution was found and the majority of the reagent was converted to dipentyl ether when NaOH was used both in equimolar and at some molar excess to 1-bromopentane and heptakis(2,6-di-*O*-methyl)-βCD (DIMEB). Although the formation of the Na salt was successful, the methyl groups and sodium ion completely covered the reaction centers. There was also not enough room for the fourth participant in the reaction despite the bulk bromine being close to sodium.

These unsuccessful reactions effectively demonstrate the importance of steric rigidity in solid state reactions.

## 3. Materials and Methods

### 3.1. General Information

β-Cyclodextrin hydrate was a generous gift from Roquette Frères (Lestrem, France). All other reagents were purchased from Alfa Aesar (Karlsruhe, Germany) and used without any further purification. Activated, strong ion-exchangers (Amberlite IRA-402(OH) and Amberlite^®^ IR-120(H)) were washed with water and then MeOH before use until the liquid phases were found to be colorless and poorly UV-active. They were dried at r.t overnight and stored in a deep-freezer until use. The dialysation cassette, Slide-A-Lyzetr 2k G2, was from Thermo Scientific (Waltham, MA, USA).

Ball mill: Retsch PM100 High Speed Planetary Ball Mill, 500 steel balls of 1 mm diameter (14.49 g) and 50 steel balls of 5 mm diameter (25.54 g, total weight of balls = 40.0 g), in a jar of 50 mL, sun wheel speed 650 min^−1^, the rotation direction was changed every 15 min, weight = 733.6 g (jar, cap, and balls).

Thermometer: Lafayette TRI-88 no-contact thermometer with built-in laser pointer, with ±2 °C reading accuracy, distance to spot size = 8:1, measuring distance 18–23 cm. The measurement matrix formed “a five on a die”, two measurements were made at each point and the values were averaged.

NMR: ^1^H and HSQC-DEPT (Heteronuclear Single Quantum Correlation-Distortionless Enhanced by Polarization Transfer) spectra were recorded at 20 °C on a Bruker Avance 300 MHz (Billerica, MA, USA) at 300.13 MHz and 75.47 MHz, respectively. The ^1^H-NMR spectra were obtained using standard pulse programs from the Bruker library and 16 K data points of 64 transients in a 4496.40 Hz spectral width. The HSQC-DEPT experiments were acquired using the pulse sequence *hsqcedtpg* from the Bruker library using 128 × 64 data points of 32 transients. In addition, **4** NMR analysis were performed on a Varian System 600 MHz spectrometer (Palo Alto, CA, USA). Solvent signals were used for referencing. NMR data were processed with ACD/NMR Processor Academic Edition Release 12.00 product version 12.01 build 39104, (Advanced Chemistry Development Inc., Toronto, Ontario, ON, Canada).

Calculation of DS was done by setting the anomeric proton signal (around 5–5.3 ppm, see Table S1 in Supplementary Materials) integral to 7.0. DS of carboxymethylated CD was calculated from the all-other-proton (≈3.4–4.2 ppm, see Table S1 in Supplementary Materials) integral by the following formula DS ≈ (all-other-proton-integral-42)/2. DS of carboxyethylated and carbamoylethylated CDs were calculated from the β-carbon (from the attachment) of the propionyl moiety (≈2.3–2.6 ppm, see Table S1 in Supplementary Materials) integral by dividing 2. DS of sulfobutylated CD was done as for CECD but δ-carbon (from the attachment) of the sulfobutyl moiety (≈2.8–3.1 ppm, see Table S1 in Supplementary Materials) integral was used. In all cases, the calculated values were checked by calculations used the CD core proton (other than anomeric proton) + substituent CH_2_ proton integrals. Approximate impurity quantitation has been made from the integrals of the impurity signals.

ESI (Electrospray Ionization) mass spectra were recorded on Waters MicromassZQ equipment (Milford, MA, USA) in positive mode in MeOH/formic acid solution of product prepared in solution and carbamoylethylβCD prepared in ball mill using MassLynx v5.5 software (Waters Corp., Milford, MA, USA). Peak identifications were made by the aid of mMass v5.5 (Open Source Mass Spectrometry Tool) software [24].

IR spectra were recorded on a PerkinElmer 1005 reflection IR spectrometer ( Waltham MA, USA) in KBr matrix using 2 cm^−1^ resolution and 32 scans.

Capillary Electrophoresis: Fingerprint electropherograms were recorded with a Hewlett-Packard ^3D^CE equipped with Diode Array Detector (Waldbronn, Germany) using uncoated fused-silica capillary of 33.5 cm (25 cm effective length) × 50 μm and indirect detection (375 μm OD) (Composite Metal Services Ltd., Worcestershire, UK). Indirect detection used benzoic acid (30 mM) (sample: 350 nm/20 nm; reference: 225 nm/20 nm). Analyses were performed in tris(hydroxyl-methyl)aminomethane buffer (60 mM, pH = 8.0). Injection at 25 mbar × 4 s at 25 °C using +20 kV voltage.

Ultrasonication: Sonica US Cleaner (130 w), Soltec srl., Milano, Italy.

TLC: TLC experiments used Merck 5554 Silicagel 60, a saturated chamber, the regular running distance was 7 cm. Samples were dissolved in DMF or water at a 10% concentration. Runs were performed in a saturated chamber with 10:7 (*v*/*v*) 1,4-dioxane:cc. NH_3_ solvent system. Each plate was run twice under the same conditions, and the plates between the two runs were dried at room temperature until NH_3_ had been completely removed. The completely dry and NH_3_-free TLC plate was sprayed with 20% cc. H_2_SO_4_/EtOH and heated for min 10 min at 100–105 °C. Organic materials are seen as black spots. R_F_ values are presented in the Supplementary Materials in Table S4.

DS values were calculated from the ^1^H-NMR spectrum, and yields are based on the calculated DS. Amounts of reagent related impurities were calculated from the ^1^H-NMR spectrum, and they are approximate values because the NMR experiments had not been optimized for quantitation.

### 3.2. Solution Reactions

#### 3.2.1. Synthesis of Randomly Carboxymethylated βCD (**2**)

The syntheses were carried out with minor modifications of [25]. Air-dried (contains 14% water) βCD (33.0 g, 0.025 m) was suspended in water (60 mL) that contains chloroacetic acid sodium salt (15.0 g, 0.125 m) at 25 °C. The reaction mixture was warmed and the dropwise addition of NaOH solution (6.0 g, 0.15 m in 14 mL water) started when the temperature reached 65 °C. The addition was carried out at such a rate to ensure that the reaction temperature remained in the 70 ± 5 °C range (≈50 min). As the addition finished, a further 2 h of stirring at the above temperature completed the reaction. The reaction mixture was allowed to cool to room temperature and stirred at r.t. overnight.

The pH of the reaction mixture was adjusted to <3 using a strong cation-exchanger (30 g), which was filtered and washed with water (90 mL). The solution of the product was concentrated to ~1/3 and the product precipitated with an EtOH-*i*PrOH (85:15 *v*/*v*, 600 mL) mixture. The solvents were decanted, the residue was dissolved in water (70) and the residual reagent/by-product was removed using a mixture of an activated strong anion- (40 g) and cation-exchanger (10 g), and was then clarified with activated carbon (2 g). Solids were filtered off and washed with water (100 mL) and the pH was then adjusted to 6.0–6.5 using 1 M NaOH. The product was isolated as a white, light electrostatic powder by freeze-drying. Yield: 33.0 g (88.6%), DS = 4.3–4.5 (^1^H-NMR), contains <1.5% (≈0.15 m) diglycolic acid.

#### 3.2.2. Synthesis of Randomly Carboxyethylated βCD (**3**)

Air-dried (contains 14% water) βCD (3.30 g, 0.0025 m) was dissolved in water (7 mL) that contained NaOH (0.6 g, 0.015 m, 6 molar-fold to CD) at r.t. and then cooled to 0–5 °C. Acrylamide (0.96 g, 0.0125 m) was added and allowed to warm to r.t. The reaction mixture was stirred for one day at r.t. and then boiled for 8 h. A dialysis cassette (MWCO 2k) was filled with the cold reaction mixture and dialyzed for 24 h against deionized water. The dialyzed solution was treated with a strong anion- (2 g) and cation-exchanger (1.0 g) for 2 h and then clarified with active carbon from 30 min. The solids were filtered off, washed with water (10 mL) and the pH was adjusted to 6.0–6.5 with 1 M NaOH. The product was solidified by freeze-drying. Yield: 2.02 g (56.3%), DS = 3.0–3.4 (^1^H-NMR).

Conversion to acidic form (for IR): 0.050 g was dissolved in deionized water (2 mL) and stirred with a strong cation exchanger (H^+^-form, 0.5 g) overnight at room temperature. The resin was filtered off, washed with water (2 mL) and freeze-dried. Comparative IR in the carbonyl region of both the protonated (1725 cm^−1^) and ionic form (1577 cm^−1^) of the product with the carbamoylethyl derivatives (compound **5**, item 9 and 10) showed the lack of amide peak (1664 cm^−1^). For spectra, see the Supplementary Materials, Figure S27.

#### 3.2.3. Synthesis of Randomly Sulfobutylated βCD (**4**)

The synthesis was carried out with minor modifications (downsized) of a known method [22]. Air-dried (contains 14% water) βCD (33.0 g, 0.025 m) was suspended in water (45 mL) and NaOH (9.3 g, 0.233 m, 9.3 molar-fold to CD) was added without cooling when the temperature of the reaction mixture reached 50–55 °C. The reaction was heated and the addition of 1,4-butanesultone (22.0 mL, 29.3 g, 0.215 m, 8.6 molar-fold to CD) started from 60–62 °C and finished at 70–75 °C (≈45 min). The reaction mixture was stirred for another 4 h at 70–75 °C when EtOH (95%, 5 mL) was added to decompose the unreacted, complexed 1,4-butanesultone and accelerate its hydrolysis. The reaction mixture was stirred for an additional 4 h at 95–105 °C and then allowed to cool to r.t.

A strong cation-exchanger (25) was added and stirred for 2 h and then clarified with activated carbon (5 g) for 2 h. The suspension was filtered, washed with water (50 mL) and a strong anion-exchanger (10 g) was added to the solution and stirred for 1.5 h before a new portion of cation-exchanger (15 g) was added and stirred for another 3.5 h. The reaction mixture was repeatedly clarified with charcoal (5 g) for 2 h. After the removal and washing of the solids, the pH of the solution was adjusted to 6.5–7.0 using 1 M NaOH and freeze-dried. Yield: 39.1 g (65.9%), DS = 6.2–6.5 (^1^H-NMR). Residual 1,4-butanesultone was <0.2%.

##### 4-Hydroxybutane-1-Sulfonic Acid Sodium Salt (Dominant Impurity of Compounds ***4*** and ***4*′**)

1,4-Butanesultone (1.02 mL, 1.36 g, 0.010 m) was slowly added with a syringe to a boiling 1 N NaOH (15 mL) solution. As the addition was completed, the reaction mixture was stirred for an additional 4 h under reflux. The reaction mixture was cooled to room temperature and stirred overnight with a strong cation-exchanger (5 g). The resin was filtered and washed with water (15 mL) and the pH was adjusted to 7.0–7.5 and the solution was freeze dried. Yield: 1.73 g, 98% white slightly hygroscopic powder.

##### Di(1,1’-sulfonatobutyl)ether Disodium Salt(Potential Impurity of Compounds ***4*** and ***4*′**)

4-hydroxybutane-1-sulfonic acid sodium salt (0.44 g, 0.0025 m) was suspended in 1,4-butanesultone (0.41 mL, 0.54 g, 0.004 m) at room temperature, powdered NaOH (0.10 g, 0.0275 m) was added and the reaction mixture was stirred at 100 °C for 4 h. The reaction mixture was cooled to room temperature and treated with 10 mL dried acetone. The solids were filtered, washed with acetone (3 mL) and then dried. The obtained crude was dissolved in water (10 mL) and freeze-dried. Yield: 0.81 g, 61%, white or pale yellow slightly hygroscopic solid.

### 3.3. Reactions in Ball Mill

#### 3.3.1. Drying of βCD

Air-dried βCD (7.00 g) was dried in a vacuum oven at 95–105 °C for a day in the presence of P_2_O_5_ and KOH. It yielded 6.02 g (86%) of a light, very electrostatic, very hygroscopic powder.

#### 3.3.2. General Method of the HEBM Reactions

βCD (0.0010 m, 1.14 g dried or 1.32 g air-dry) was ball milled with sodium hydroxide for 10 min before the addition of the reagents. When the ball milling was finished, water (10 mL) was added to the reaction mixture, except in the *n*-butyl acrylate reactions (2′, items 5 and 6 in Table 1), and the solutions were transferred to an Erlenmeyer flask and stirred with a strong cation-exchanger (2.5 g) for 1 h. The balls and jar then were washed with water (30 mL). Activated carbon (0.15 g, sulfobutyl reactions: 0.8 g) and a strong anion-exchanger (1.5 g) was added and stirred for another hour. Solids were filtered off, washed with water (15 mL) and the filtrate was sonicated for 30 min with toluene (50 µL), except in the sulfobutyl reactions (3′, items 11–16 in Table 1), and allowed to stand overnight. The precipitate was filtered off using a thin active carbon layer and neutralized with a satd. sodium hydrocarbonate solution to pH ≈ 7, and then freeze-dried.

#### 3.3.3. Synthesis of Randomly Carboxymethylated βCD (**2’**)

Reagents used, milling time, temperatures at the end of the reactions and yield can be seen in Table 2. Residual βCD content of the products was below 1%, as measured by TLC.

#### 3.3.4. Synthesis of Randomly Carboxyethylated βCD (**3′**)

Reagents used, milling time, temperatures at the end of reactions and yield can be seen in Table 3. The work-up of the *n*-butyl acrylate reactions was different from the general procedure because the majority of the reaction mixture was not soluble in water at the end of the milling process and the sealing of the jar was not acetone resistant. The reaction mixture was dissolved in acetone (30 mL), while the jar and balls were washed with acetone (50 mL) and water (30 mL). The two solutions were combined and an additional portion of acetone (50 mL) was added, and the solution was allowed to stand for 3 days at room temperature, after which a waxy precipitate formed. The liquid phase was decanted, washed with acetone (10 mL), and then the acetone was allowed to evaporate. The residue was dissolved in water (10 mL), sonicated for 30 min and then allowed to crystallize overnight. The precipitate was filtered off through a thin active carbon filter and freeze-dried.

Residual βCD content of the products was below 1% as measured by TLC.

#### 3.3.5. Direct Synthesis of Randomly Carboxyethylated βCD (**3′′**)

Sodium acrylate was prepared from aqueous solutions of acrylic acid and sodium hydrocarbonate with freeze-drying. The work-up of the Na acrylate was identical to the general procedure described above. Residual βCD content of the product was below 1% as measured by TLC.

#### 3.3.6. Synthesis of Randomly Carbamoylethylated βCD (**5**)

Reagents used, milling time, temperatures at the end of reactions and yield are in Table 4. After the ion-exchanger treatment, the pH of the solutions was 5–6, identical with the water used and no neutralization was carried out. The first reaction (item 9 in Table 1) was monitored by NMR for acrylic content. Sampling (≈15 mg) was performed after 0.5, 1.5, 2.5, 3.5, 4.5 and 5.5 h milling and samples were analyzed using ^1^H-NMR without neutralization in D_2_O.

#### 3.3.7. Synthesis of Randomly Sulfobutylated βCD (**4′**)

Reagents used, milling time, temperatures at the end of reactions and yield are in Table 5. In case of item 15, the purification was repeated to further reduce the 4-hydroxy-butanesulfonic acid content.

## 4. Conclusions

Mechanochemical syntheses of anionic βCD derivatives were more effective than analogous solution reactions due to the suppressed hydrolysis of reagents. Substitution reactions in the solid state also preferred the secondary hydroxyls, particularly the C(2) hydroxyls. Higher DS and peculiar substitution distribution patterns were found in the mechanochemical reactions as compared to the solution reactions. The one-step synthesis of CEβCD was successfully carried out using a weak Michael acceptor, sodium acrylate, and, although the DS of the product was close to the solution reaction, the DS distribution was quite different not only from the solution but from the HEBM reactions too, which used an acrylic ester as Michael acceptor. Based on our results, the synthesis carboxymethylation seems to be substituted with a mechanochemical method while, at present, the products of carboxyethylation and sulfobutylation are far from that of the solution synthesis. Bulky reagents also showed poor reactivity in S_N_2-type reactions in ball milling.

## Figures and Tables

**Table 1:** Summary of solution and HEBM reactions (shaded lines are the solution reactions).

No	Compound	Reagent ^1^	NaOH/Reagent/CD (mole:mole:mole)	Reaction Time (h)	Yield (%)	DS	Residual Reagent ^2^ (%)
1	2	ClAcONa ^3^	6:5:1	3	89	4.3–4.5	<2.5
2	2’	ClAcONa	6:5:1	1.5	96	4.3–4.6	<2.5
3	2’	ClAcONa	6:5:1	2.5	98	4.4–4.6	<0.5
4	2’	ClAcONa ^4^	6:5:1	2.5	77	4.4–4.7	<1.0
5	3’	BuOAcr	6:5:1	1.5	50	3.0–3.3	≈1.0
6	3’	BuOAcr ^4^	6:5:1	2.5	30	2.8–3.2	<1.0
7	3”	AcrONa	6:5:1	3.5	52	2.8–3.1	<11
8	3	AcrAm ^3^	6:5:1	24 + 8	56	3.0–3.4	<0.05
9	5	AcrAm	6:5:1	5.5	73	3.8–4.3	<0.9
10	5	AcrAm	6:5:1	3.5	83	3.3–3.5	<0.5
11	4	BS ^3^	9.3:8.6:1	8	66	6.2–6.5	<0.2 ^5^
12	4’	BS	9:8:1	1.5	56	3.1–3.3	≈17 ^5^
13	4’	BS	9:8:1	2.5	57	5.2–5.8	≈9.5 ^5^
14	4’	BS	16:8:1	2.5	67	6.2–6.6	<2 ^5^
15	4’	BS ^4^	16:8:1	2.5	72	7.5–7.8	<0.6 ^5^
16	4’	BS ^6^	16:8:1	3 (1.5 + 1.5)	71	4.4–4.9	≈9 ^5^
17	4’	BS	16:8:1	3.5	79	6.9–7.5	≈0.6 ^5^

^1^ ClAcONa: Sodium chloroacetate, BuOAcr: *n*-butyl acrylate, AcrONa: sodium acrylate, AcrAm: acrylamide, BS: 1,4-butanesultone; ^2^ or reagent derived by-product, calculated from ^1^H-NMR; ^3^ solution reaction; ^4^ from dried βCD (air dry βCD contains ≈14% water which was removed at 95–105 °C under reduced pressure using P_2_O_5_ and KOH); ^5^ residual 1,4-butanesultone; ^6^ BS was added in two equal portions.

**Table 2:** Summary of carboxymethylation HEBM reactions (numbering is identical with Table 1).

No	NaOH (g (mole))	ClAcONa (g (mole))	Milling Time (h)	Final Temp. (°C)	Yield (g (%))	DS	Residual Reagent ^1^ (%)
2	0.24 (0.006)	0.61 (0.005)	1.5	84	1.42 (95.9)	4.3–4.6	<2.5
3	0.24 (0.006)	0.61 (0.005)	2.5	87	1.48 (98.4)	4.4–4.6	<0.5
4	0.24 (0.006)	0.61 (0.005) ^2^	2.5	87	1.15 (76.7)	4.4–4.6	<1.0

^1^ Or reagent derived by-product; ^2^ dried βCD was used.

**Table 3:** Summary of carboxyethylation HEBM reactions (numbering is identical with Table 1).

No	NaOH (g (mole))	Reagent (g (mole))	Milling Time (h)	Final Temp. (°C)	Yield (g (%))	DS	Residual Reagent ^1^ (%)
5	0.24 (0.006)	0.64 (0.005) ^2^	2.5	67	0.72 (50.3)	3.0–3.3	≈1.0
6	0.24 (0.006)	0.64 (0.005) ^2,3^	3.5	70	0.42 (29.6)	2.8–3.2	<1.0
7	0.24 (0.006)	0.54 (0.005) ^4^	3.5	93	0.74 ^5^ (52.1)	2.8–3.1	<11

^1^ Or reagent derived by-product; ^2^*n*-butyl acrylate; ^3^ dried βCD; ^4^ reaction of sodium acrylate; ^5^ corrected with sodium acrylate content.

**Table 4:** Summary of carbamoylethylation HEBM reactions (numbering is identical with Table 1).

No	NaOH (g (mole))	Reagent (g (mole))	Milling Time (h)	Final Temp. (°C)	Yield (g (%))	DS	Residual Reagent ^1^ (%)
9	0.24 (0.006)	0.36 (0.005)	5.5	97	1.05 (73.4)	3.8–4.3	<0.9
10	0.24 (0.0051)	0.36 (0.005)	3.5	88	1.15 (83.3)	3.3–3.5	<0.5

^1^ Or reagent derived by-product.

**Table 5:** Summary of sulfobutylation HEBM reactions (numbering is identical with Table 1).

No	NaOH (g (mole))	BS (g (mole))	Milling Time (h)	Final Temp. (°C)	Yield (g (%)) ^1^	DS	Residual Reagent ^2^ (%)
12	0.36 (0.009)	1.09 (0.008)	1.5	67	1.17 (55.6)	3.1–3.4	≈22
13	0.36 (0.009)	1.09 (0.005)	2.5	70	1.24 (54.6)	5.2–5.8	≈12
14	0.64 (0.018)	1.09 (0.005)	2.5	82	1.59 (66.6)	6.2–6.6	<10
15	0.64 (0.006)	1.09 (0.005) ^3^	2.5	82	1.78 (72.2)	7.5–7.8	≈5
16	0.64 (0.018)	1.09 (0.005) ^4^	3 (1.5 + 1.5)	82/92	1.52 (70.7)	4.4–4.9	≈13
17	0.64 (0.006)	1.09 (0.005)	3.5	88	1.89 (79.1)	6.9–7.5	≈5

^1^ Calculations were corrected according to the impurities; ^2^ or reagent derived by-product; ^3^ dried βCD was used; ^4^ 1,4-butanesultone was added in two portions.

## Supplementary Materials

NMR spectra, selected IR and capillary electropherograms of the prepared materials are available.
