# Supplementary material for: Synthesis of Randomly Substituted Anionic Cyclodextrins in Ball Milling
Source: Molecules. 2017 Mar 19;22(3):485. doi: 10.3390/molecules22030485 (PMC6155213; doi:10.3390/molecules22030485)
Supplement: Supplementary file 1 [file molecules-22-00485-s001.pdf]

Supporting Information

Synthesis of randomly substituted anionic cyclodextrins in a ball mill

László Jicsinszky[1,*], Marina Caporaso[1], Emanuela Calcio Gaudino[1], Cristina Giovannoli[2], and Giancarlo Cravotto[1,*]

[1] University of Turin, Department of Drug Science and Technology, Via P. Giuria 7, 10125 Turin, Italy; laszlo.jicsinszky@unito.it (LJ); marina.caporaso@unito.it (MC); Giancarlo.cravotto@unito.it (GC).

[2] University of Turin, Department of Chemistry, Via P. Giuria 7 - 10125 Turin, Italy; cristina.giovannoli@unito.it (CG)
* Correspondence: ljicsinszky@gmail.com (LJ), Giancarlo.cravotto@unito.it (GC). Tel.: +39-011-670-7663, +39-011-670-7684

Content

Figure S 1: Proton NMR spectrum of carboxymethylated ßCD (**2**) prepared in solution (item 1 in Table 1), DS ≈4.3-4.5 **S4**
Figure S 2: HSQC-DEPT spectrum of carboxymethylated ßCD (**2**) prepared in solution (item 1 in Table 1), DS ≈4.3-4.5 **S4**
Figure S 3: ^{13}C-NMR spectrum of carboxymethylated ßCD (**2**) prepared in solution (item 1 in Table 1), DS ≈4.3-4.5 **S5**

Figure S 4: ESI+ mass spectrum of carboxymethylated ßCD (**2**) prepared in solution (item 1 in Table 1), DS ≈4.3-4.5 **S5**
Figure S 5: Peak identification in ESI+ mass spectrum of carboxymethylated ßCD (**2**) prepared in solution (item 1 in Table 1), DS ≈4.3-4.5 [1,2] **S6**

Figure S 6: Proton NMR spectrum of carboxymethylated ßCD (**2'**) prepared in ball mill (item 2 in Table 1), DS ≈4.3-4.6 **S7**
Figure S 7: HSQC-DEPT spectrum of carboxymethylated ßCD (**2'**) prepared in ball mill (item 2 in Table 1), DS ≈4.3-4.6 **S7**
Figure S 8: Proton NMR spectrum of carboxymethylated ßCD (**2'**) prepared in ball mill (item 3 in Table 1), DS ≈4.4-4.6 **S8**
Figure S 9: HSQC-DEPT spectrum of carboxymethylated ßCD (**2'**) prepared in ball mill (item 3 in Table 1), DS ≈4.4-4.6 **S8**
Figure S 10: Proton NMR spectrum of carboxymethylated ßCD (**2'**) prepared in ball mill (item 4 in Table 1), DS ≈4.4-4.6 **S9**
Figure S 11: HSQC-DEPT spectrum of carboxymethylated ßCD (**2'**) prepared in ball mill (item 4 in Table 1), DS ≈4.4-4.6 **S9**
Figure S 12: Capillary electropherogram of carboxymethylated ßCDs **S10**

Figure S 13: Proton NMR spectrum of carboxyethylated ßCD (**3**) prepared in solution (item 8 in Table 1), DS ≈3.0-3.4 **S11**
Figure S 14: HSQC-DEPT spectrum of carboxyethylated ßCD (**3**) prepared in solution (item 8 in Table 1), DS ≈3.0-3.4 **S11**
Figure S 15: ^{13}C-NMR spectrum of carboxyethylated ßCD (**3**) prepared in solution (item 8 in Table 1), DS ≈3.0-3.4 **S12**

Figure S 16: ESI+ mass spectrum of carboxyethylated ßCD (**3**) prepared in solution (item 8 in Table 1), DS ≈3.0-3.4 **S12**
Figure S 17: Peak identification in ESI+ mass spectrum of carboxyethylated ßCD (**3**) prepared in solution (item 8 in Table 1), DS ≈3.0-3.4 **S13**

Figure S 18: Proton NMR spectrum of carboxyethylated ßCD (**3'**) prepared in ball mill (item 5 in Table 1), DS ≈3.0-3.3 **S14**

Figure S 19: HSQC-DEPT spectrum of carboxyethylated ßCD (**3'**) prepared in ball mill (item 5 in Table 1), DS ≈3.0-3.3 **S14**
Figure S 20: Proton NMR spectrum of carboxyethylated ßCD (**3'**) prepared in ball mill (item 6 in Table 1), DS ≈2.8-3.2 **S15**
Figure S 21: HSQC-DEPT spectrum of carboxyethylated ßCD (**3'**) prepared in ball mill (item 6 in Table 1), DS ≈2.8-3.2 **S15**
Figure S 22: Proton NMR spectrum of carboxyethylated ßCD (**3'**) prepared in ball mill (item 7 in Table 1), DS ≈2.8-3.1 **S16**
Figure S 23: HSQC-DEPT spectrum of carboxyethylated ßCD (**3'**) prepared in ball mill (item 7 in Table 1), DS ≈2.8-3.1 **S16**

Figure S 24: IR spectrum of carboxylethylated ßCD (**3**) prepared in solution (item 8 in Table 1) as acid, DS ≈3.0-3.4 **S17**
Figure S 25: IR spectrum of carboxylethylated ßCD (**3**) prepared in solution (item 8 in Table 1) as sodium salt, DS ≈3.0-3.4 **S17**
Figure S 26: Overlay of IR spectrum of carboxylethylated ßCD acid & salt form (**3**) prepared in solution (item 8 in Table 1), DS ≈3.0-3.4 and carbamoylethylated ßCD prepared in ball mill (item 10 in table 1) **S18**

Figure S 27: Proton NMR spectrum of carbamoylethylated ßCD (**5**) prepared in ball mill (item 9 in Table 1), DS ≈3.8-4.3 **S22**
Figure S 29: 13C-NMR spectrum of carbamoylethylated ßCD (**5**) prepared in ball mill (item 9 in Table 1), DS ≈3.8-4.3 **S20**

Figure S 30: ESI+ mass spectrum of carbamoylethylated ßCD (**5**) prepared in ball mill (item 9 in Table 1), DS ≈3.8-4.3 **S20**
Figure S 31: Peak identification in ESI+ mass spectrum of carbamoylethylated ßCD (**5**) prepared in ball mill (item 9 in Table 1), DS ≈3.8-4.3 **S21**

Figure S 32: Proton NMR spectrum of carbamoylethylated ßCD (**5**) prepared in ball mill (item 10 in Table 1), DS ≈3.3-3.5 **S22**
Figure S 33: HSQC-DEPT spectrum of carbamoylethylated ßCD (**5**) prepared in ball mill (item 10 in Table 1), DS ≈3.3-3.5 **S22**
Figure S 34: 13C-NMR spectrum of carbamoylethylated ßCD (5) prepared in ball mill (item 10 in Table 1), DS ≈3.3-3.5 **S31**

Figure S 35: ESI+ mass spectrum of carbamoylethylated ßCD (**5**) prepared in ball mill (item 10 in Table 1), DS ≈3.3-3.5 **S23**
Figure S 36: Peak identification in ESI+ mass spectrum of carbamoylethylated ßCD (**5**) prepared in ball mill (item 10 in Table 1), DS ≈3.8-4.3 **S23**

Figure S 37: IR spectrum of carbamoylethylated ßCD (**5**) prepared in ball mill (item 10 in Table 1), DS ≈3.3-3.5 **S25**
Figure S 38: IR spectrum of carbamoylethylated ßCD (**5**) prepared in ball mill (item 10 in Table 1), DS ≈3.3-3.5 **S25**
Figure S 39: Overlay of IR spectra of carbamoylethylated ßCD (**5**) prepared in ball mill (item 9&10 in Table 1), DS ≈3.3-3.5 carboxyethylated ßCD prepared in solution (item 8 in Table 1) **S26**
Figure S 40: Capillary electropherogram of carboxyethylated ßCDs **S26**

Figure S 41: Proton NMR spectrum of sulfobutylated ßCD (**4**) prepared in solution (item 11 in Table 1), DS ≈6.2-6.5 **S28**
Figure S 42: HSQC-DEPT spectrum of sulfobutylated ßCD (**4**) prepared in solution (item 11 in Table 1), DS ≈6.2-6.5 **S28**

Figure S 43: ESI+ mass spectrum of sulfobutylated ßCD (**4**) prepared in solution (item 11 in Table 1), DS ≈6.2-6.5 **S29**
Figure S 44: Peak identification in ESI+ mass spectrum of sulfobutylated ßCD (**4**) prepared in solution (item 11 in Table 1), DS ≈6.2-6.5 **S30**

Figure S 45: Proton NMR spectrum of sulfobutylated ßCD (**4’**) prepared in ball mill (item 12 in Table 1), DS ≈3.1-3.3 **S31**
Figure S 46: HSQC-DEPT spectrum of sulfobutylated ßCD (**4’**) prepared in ball mill (item 12 in Table 1), DS ≈3.1-3.3 **S31**
Figure S 47: Proton NMR spectrum of sulfobutylated ßCD (**4’**) prepared in ball mill (item 13 in Table 1), DS ≈5.2-5.8 **S32**
Figure S 48: HSQC-DEPT spectrum of sulfobutylated ßCD (**4’**) prepared in ball mill (item 13 in Table 1), DS ≈5.2-5.8 **S32**
Figure S 49: Proton NMR spectrum of sulfobutylated ßCD (**4’**) prepared in ball mill (item 14 in Table 1), DS ≈6.2-6.6 **S33**
Figure S 50: HSQC-DEPT spectrum of sulfobutylated ßCD (**4’**) prepared in ball mill (item 14 in Table 1), DS ≈6.2-6.6 **S33**
Figure S 51: Proton NMR spectrum of sulfobutylated ßCD (**4’**) prepared in ball mill (item 15 in Table 1), DS ≈7.5-7.8 **S34**
Figure S 52: HSQC-DEPT spectrum of sulfobutylated ßCD (**4’**) prepared in ball mill (item 15 in Table 1), DS ≈7.5-7.8 **S34**
Figure S 53: Proton NMR spectrum of sulfobutylated ßCD (**4’**) prepared in ball mill (item 16 in Table 1), DS ≈4.4-4.9 **S35**
Figure S 54: HSQC-DEPT spectrum of sulfobutylated ßCD (**4’**) prepared in ball mill (item 16 in Table 1), DS ≈4.4-4.9 **S35**
Figure S 55: Proton NMR spectrum of sulfobutylated ßCD (**4’**) prepared in ball mill (item 17 in Table 1), DS ≈6.9-7.5 **S36**
Figure S 56: HSQC-DEPT spectrum of sulfobutylated ßCD (**4’**) prepared in ball mill (item 17 in Table 1), DS ≈6.9-7.5 **S36**
Figure S 57: Proton NMR spectrum of 4-hydroxy-1-butanesulfonic acid sodium salt (HOBSANa) **S37**
Figure S 58: HSQC-DEPT spectrum of HOBSANa **S37**
Figure S 59: Proton NMR spectrum of di(1,1’-sulfonatobutyl)ether disodium salt (disodium 4,4'-oxydibutane-1,1’-disulfonate, BIBSANa) **S38**
Figure S 60: HSQC-DEPT spectrum of BIBSANa **S38**
Figure S 61: Capillary electropherogram of sulfobutylated ßCDs **S39**

Scheme S 1: Carbon atom numbering for the NMR assignment (OH: unsubstituted; OR: substituted) **S40**

Table S 1: Proton assignment of cyclodextrin derivatives prepared in solution(based on ^{1}H- and HSQC-DEPT experiments) **S40**
Table S 2: Carbon assignment of cyclodextrin derivatives prepared in solution (based on ^{13}C- and HSQC-DEPT experiments) **S41**

Table S 3: Characteristic C=O IR bands of the prepared (carbonyl)ethyl group containing CD derivatives **S41**

Table S 4: R_F-values of the prepared compounds after twice run of the same plate in the same solvent mixture at 7 cm distance in 100 µg **S41**

References **S42**

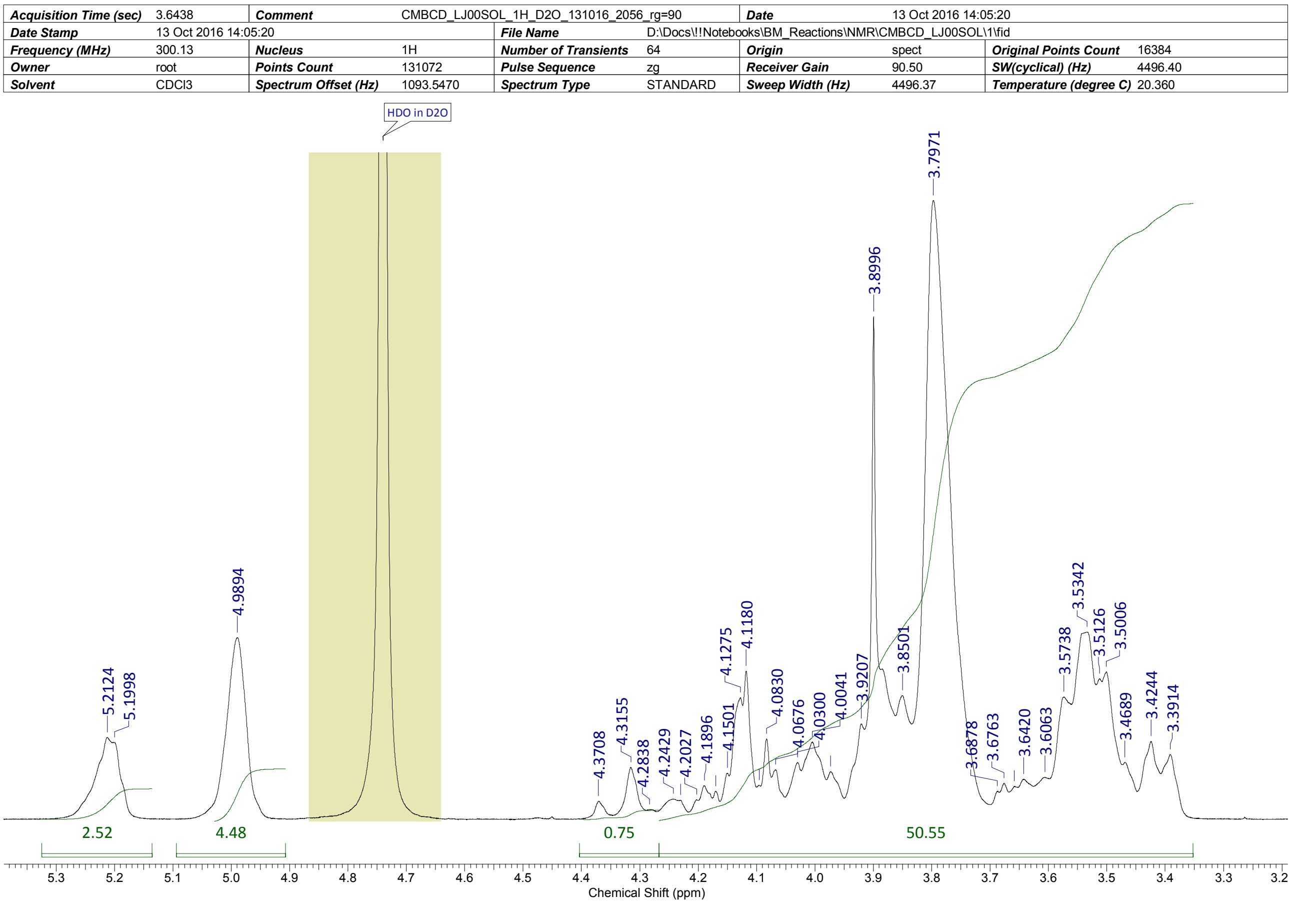

*Figure S 1: Proton NMR spectrum of carboxymethylated βCD (**2**) prepared in solution (item 1 in Table 1), DS ≈4.3-4.5*

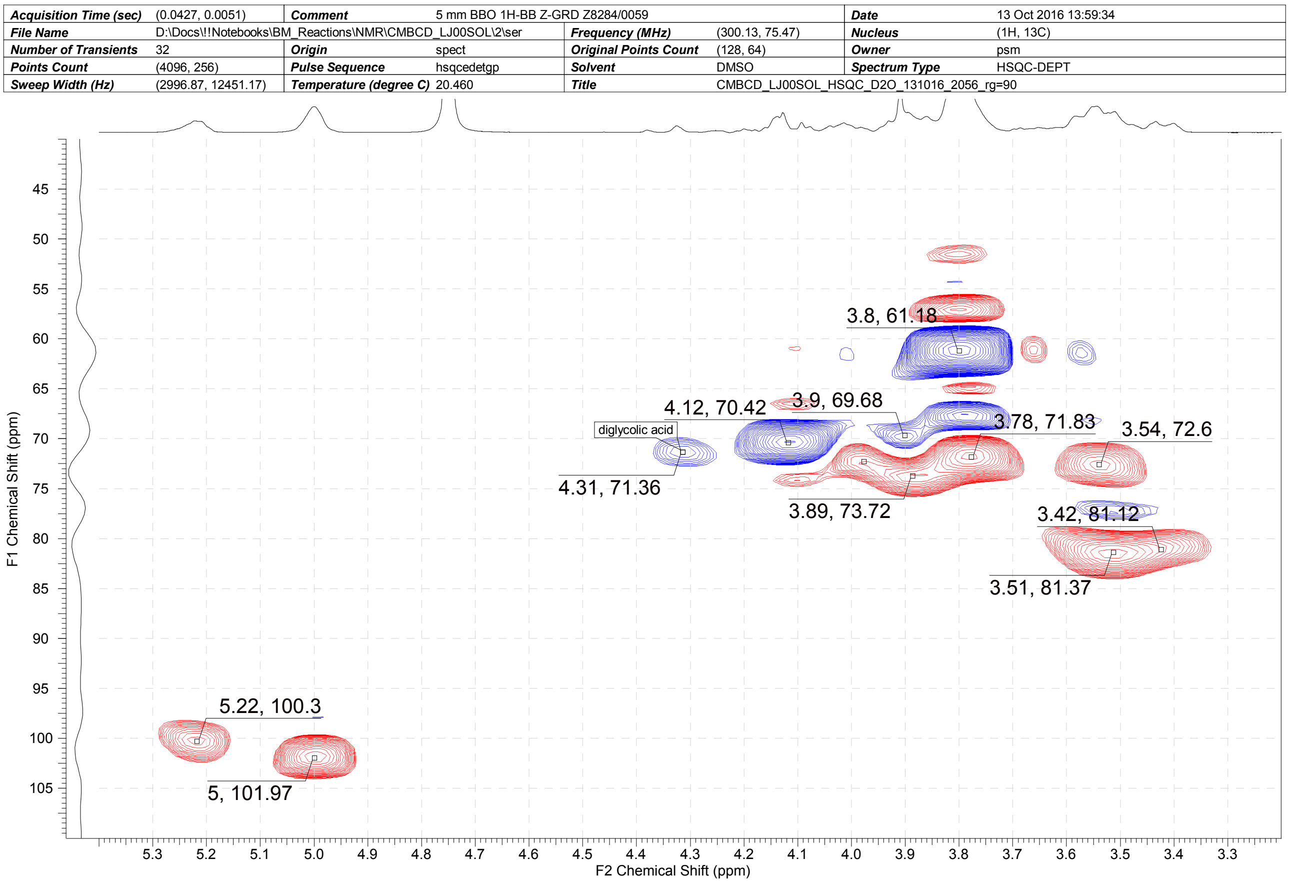

*Figure S 2: HSQC-DEPT spectrum of carboxymethylated βCD (**2**) prepared in solution (item 1 in Table 1), DS ≈4.3-4.5*

Acquisition Time (sec)	1.8088	Comment	CMBCD_LJ02_13C_D2O_230117_2134_RG=100			Date	23 Jan 2017 08:10:56		
Date Stamp	23 Jan 2017 08:10:56			File Name	E:\Documents\!!Molecules\Anal\CarbonNMR\CMBCD_LJ02\2\fid				
Frequency (MHz)	75.47	Nucleus	13C	Number of Transients	627	Origin	spect	Original Points Count	32768
Owner	root	Points Count	262144	Pulse Sequence	zgpg.save.txt	Receiver Gain	13004.00	SW(cyclical) (Hz)	18115.94
Solvent	D2O	Spectrum Offset (Hz)	8301.4463	Spectrum Type	STANDARD	Sweep Width (Hz)	18115.87	Temperature (degree C)	20.460

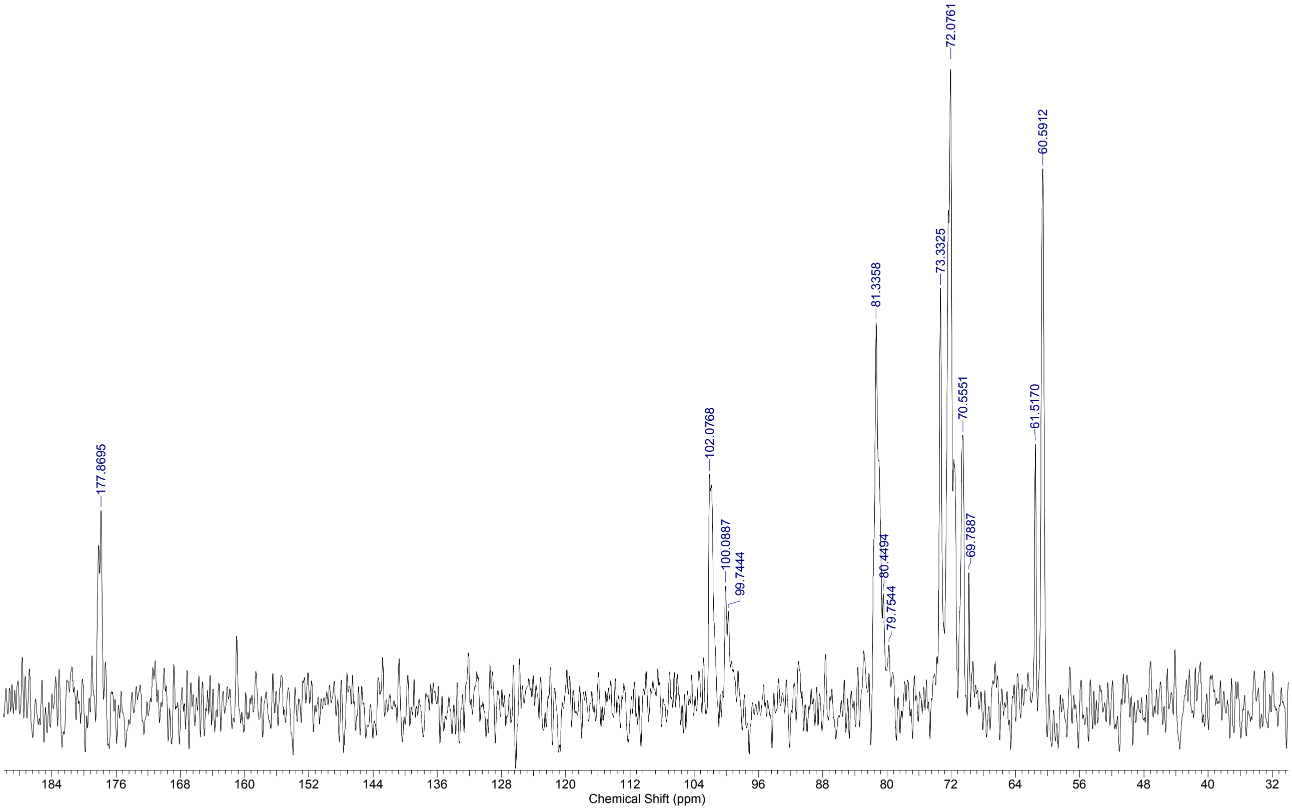

*Figure S 3: ^{13}C-NMR spectrum of carboxymethylated βCD (**2**) prepared in solution (item 1 in Table 1), DS ≈4.3-4.5*

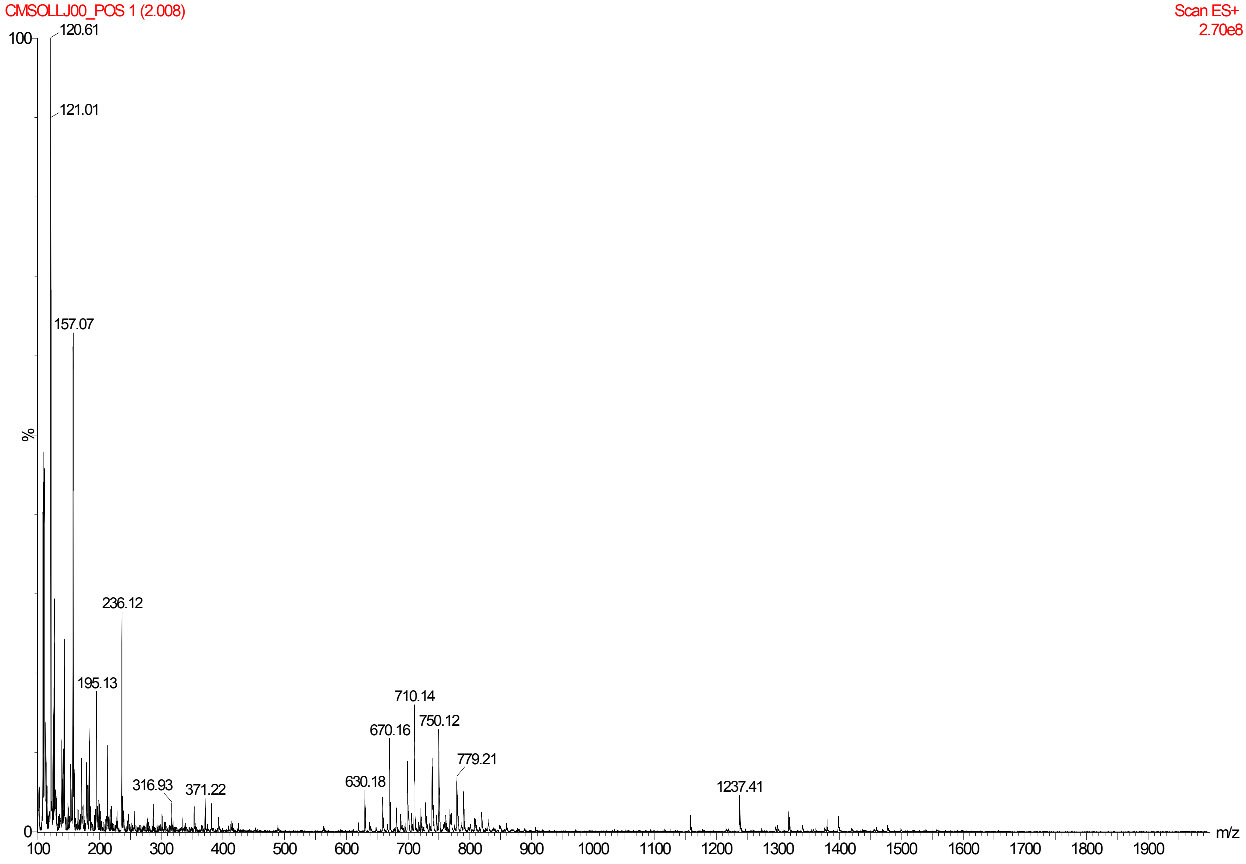

*Figure S 4: ESI+ mass spectrum of carboxymethylated βCD (**2**) prepared in solution (item 1 in Table 1), DS ≈4.3-4.5*

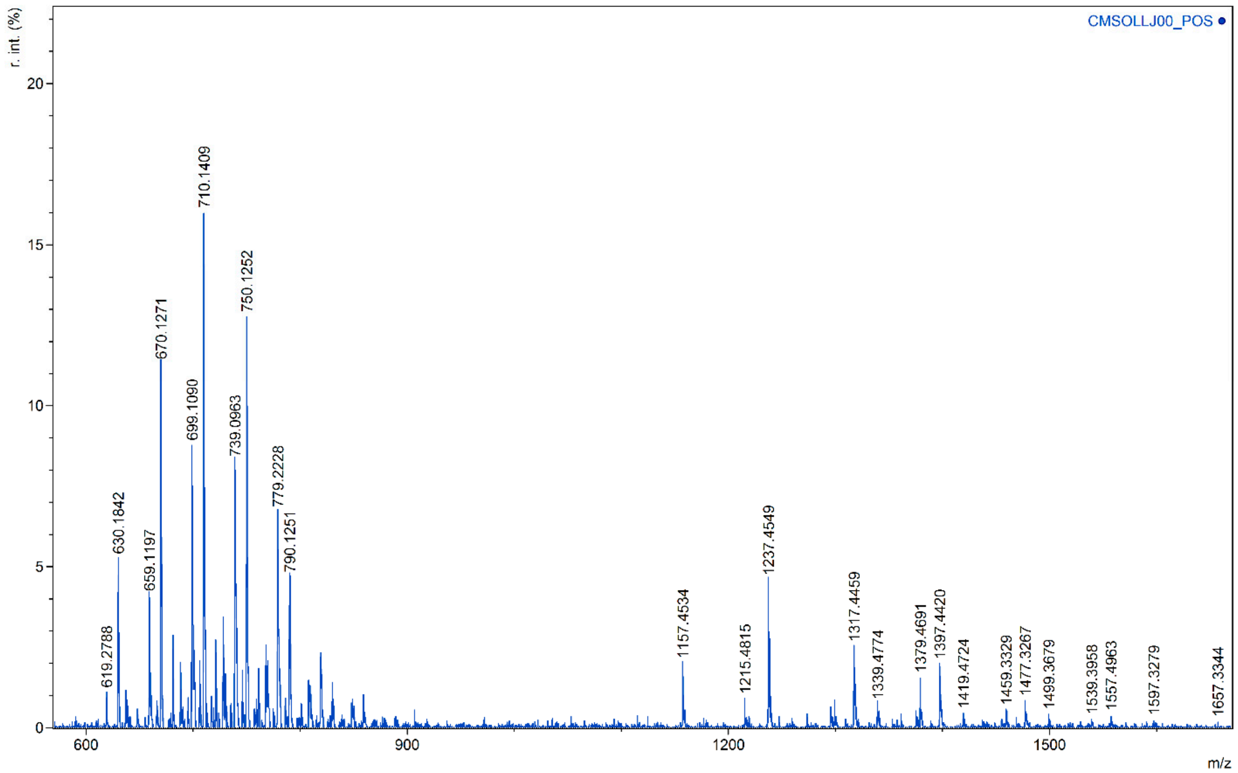

Meas. m/z	Calc. m/z	δ (Da)	z	Annotation	Formula
630.1842	630.1678	0.0164	2	(+1 CH2COONa)/2	(C42H70O35Na2)1(CH1COONa)1
659.1197	659.1706	-0.0509	2	(+1 CH2COOH+1 CH2COONa)/2	(C42H70O35Na2)1(CH1COOH)1(CH1COONa)1
699.1090	699.1760	-0.0670	2	(+1 CH2COOH +2 HCOOH +1 HCOONa)/2	(C42H70O35Na2)1(CH1COOH)1(CH1COONa)0(HCOOH)2(HCOONa)1(H2O)0
710.1409	710.1670	-0.0261	2	(+1 CH2COOH +1 HCOOH +2 HCOONa)/2	(C42H70O35Na2)1(CH1COOH)1(CH1COONa)0(HCOOH)1(HCOONa)2(H2O)0
739.0963	739.1697	-0.0734	2	(+2 CH2COOH +1 HCOOH +2 HCOONa)/2	(C42H70O35Na2)1(CH1COOH)2(CH1COONa)0(HCOOH)1(HCOONa)2(H2O)0
750.1252	750.1607	-0.0355	2	(+2 CHCOOH +3 HCOONa)/2	(C42H70O35Na2)1(CH1COOH)2(CH1COONa)0(HCOOH)0(HCOONa)3(H2O)0
779.2228	779.1635	0.0593	2	(+3 CH2COOH +3 HCOONa)/2	(C42H70O35Na2)1(CH1COOH)3(CH1COONa)0(HCOOH)0(HCOONa)3(H2O)0
790.1251	790.1544	-0.0293	2	(+2 CH2COOH + 1 CH2COONa +3 HCOONa)/2	(C42H70O35Na2)1(CH1COOH)2(CH1COONa)1(HCOOH)0(HCOONa)3(H2O)0
1157.4534	1157.3590	0.0944	1	C42H70O35 (βCD)	C42H70O35Na
1215.4815	1215.3645	0.1171	1	+1 CH2COOH	(C42H70O35Na)CH1COOH
1237.4549	1237.3464	0.1085	1	+1 CH2COONa	(C42H70O35Na)CH1COONa
1317.4459	1317.3338	0.1120	1	+2 CH2COONa	(C42H70O35Na)1(CH1COOH)0(CH1COONa)2(HCOOH)0(HCOONa)0(H2O)0
1379.4691	1379.4177	0.0515	1	+1 CH2COOH +2 HCOOH +4 H2O	(C42H70O35Na)1(CH1COOH)1(CH1COONa)0(HCOOH)2(HCOONa)0(H2O)4
1397.4420	1397.3213	0.1207	1	+3 CH2COONa	(C42H70O35Na)1(CH1COOH)0(CH1COONa)3(HCOOH)0(HCOONa)0(H2O)0
1419.4724	1419.3267	0.1457	1	+1 CH2COOH +3 HCOONa	(C42H70O35Na)1(CH1COOH)1(CH1COONa)0(HCOOH)0(HCOONa)3(H2O)0
1459.3329	1459.4051	-0.0722	1	+1 CH2COOH +1 CH2COONa	(C42H70O35Na)1(CH1COOH)1(CH1COONa)1(HCOOH)2(HCOONa)0(H2O)4
1477.3267	1477.4181	-0.0913	1	+3 CH2COOH +2 HCOOH +3 H2O	(C42H70O35Na)1(CH1COOH)3(CH1COONa)0(HCOOH)2(HCOONa)0(H2O)3
1499.3679	1499.4000	-0.0322	1	+3 CH2COOH +1 HCOOH +1 HCOONa +3 H2O	(C42H70O35Na)1(CH1COOH)3(CH1COONa)0(HCOOH)1(HCOONa)1(H2O)3
1539.3958	1539.3925	0.0032	1	+3 CH2COOH +2 HCOONa +4 H2O	(C42H70O35Na)1(CH1COOH)3(CH1COONa)0(HCOOH)0(HCOONa)2(H2O)4
1557.4963	1557.3196	0.1767	1	+2 CH2COOH +1 CH2COONa +3 HCOONa	(C42H70O35Na)1(CH1COOH)2(CH1COONa)1(HCOOH)0(HCOONa)3(H2O)0
1597.3279	1597.3980	-0.0701	1	+4 CH2COOH +2 HCOONa +4 H2O	(C42H70O35Na)1(CH1COOH)4(CH1COONa)0(HCOOH)0(HCOONa)2(H2O)4
1657.3344	1657.4215	-0.0872	1	+6 CH2COOH +1 CH2COONa +4 H2O	(C42H70O35Na)1(CH1COOH)6(CH1COONa)1(HCOOH)0(HCOONa)0(H2O)4

*Figure S 5: Peak identification in ESI+ mass spectrum of carboxymethylated βCD (**2**) prepared in solution (item 1 in Table 1), DS ≈4.3-4.5 [1,2]*

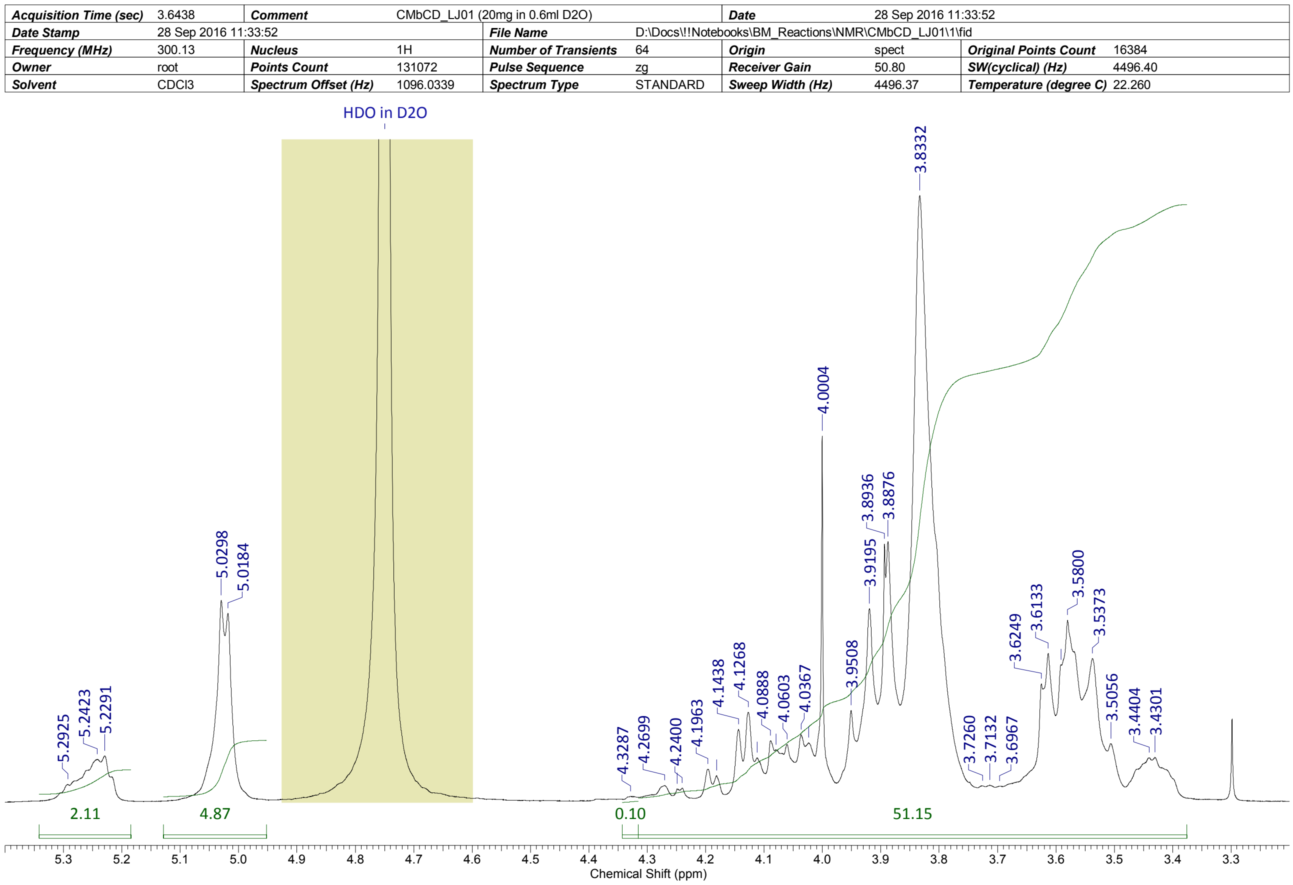

*Figure S 6: Proton NMR spectrum of carboxymethylated βCD (**2'**) prepared in ball mill (item 2 in Table 1), DS ≈4.3-4.6*

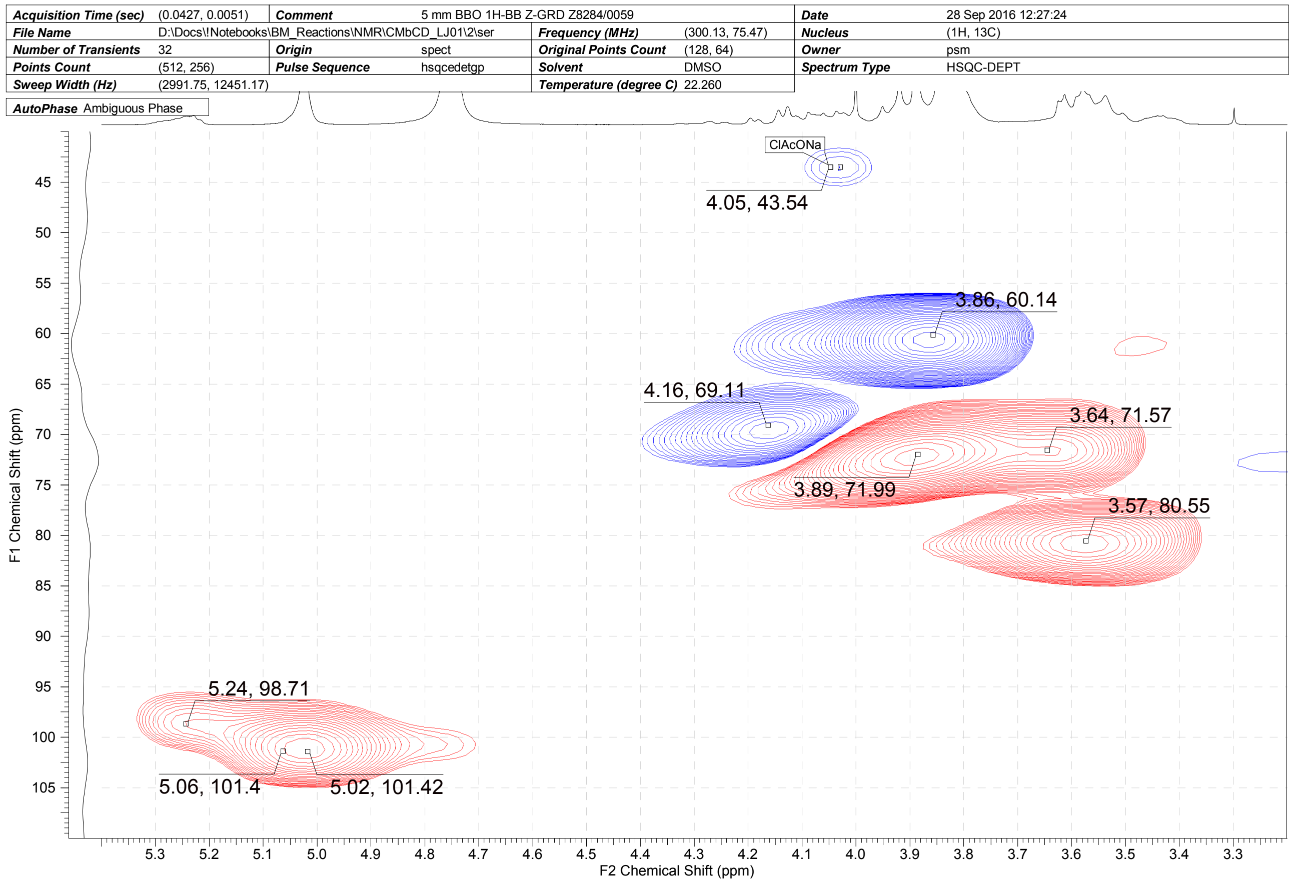

*Figure S 7: HSQC-DEPT spectrum of carboxymethylated βCD (**2'**) prepared in ball mill (item 2 in Table 1), DS ≈4.3-4.6*

Acquisition Time (sec)	3.6438	***Comment***	CBCD_LJ02 (20mg in 0.6ml D2O)			***Date***	30 Sep 2016 15:20:00		
Date Stamp	30 Sep 2016 15:20:00			***File Name***	D:\Docs\!!Notebooks\BM_Reactions\NMR\CMBCD_LJ02\1\fid				
Frequency (MHz)	300.13	***Nucleus***	1H	***Number of Transients***	64	***Origin***	spect	***Original Points Count***	16384
Owner	root	***Points Count***	131072	***Pulse Sequence***	zg	***Receiver Gain***	101.60	***SW(cyclical) (Hz)***	4496.40
Solvent	DEUTERIUM OXIDE			***Spectrum Offset (Hz)***	1096.2397	***Spectrum Type***	STANDARD	***Sweep Width (Hz)***	4496.37
Temperature (degree C)	22.160								

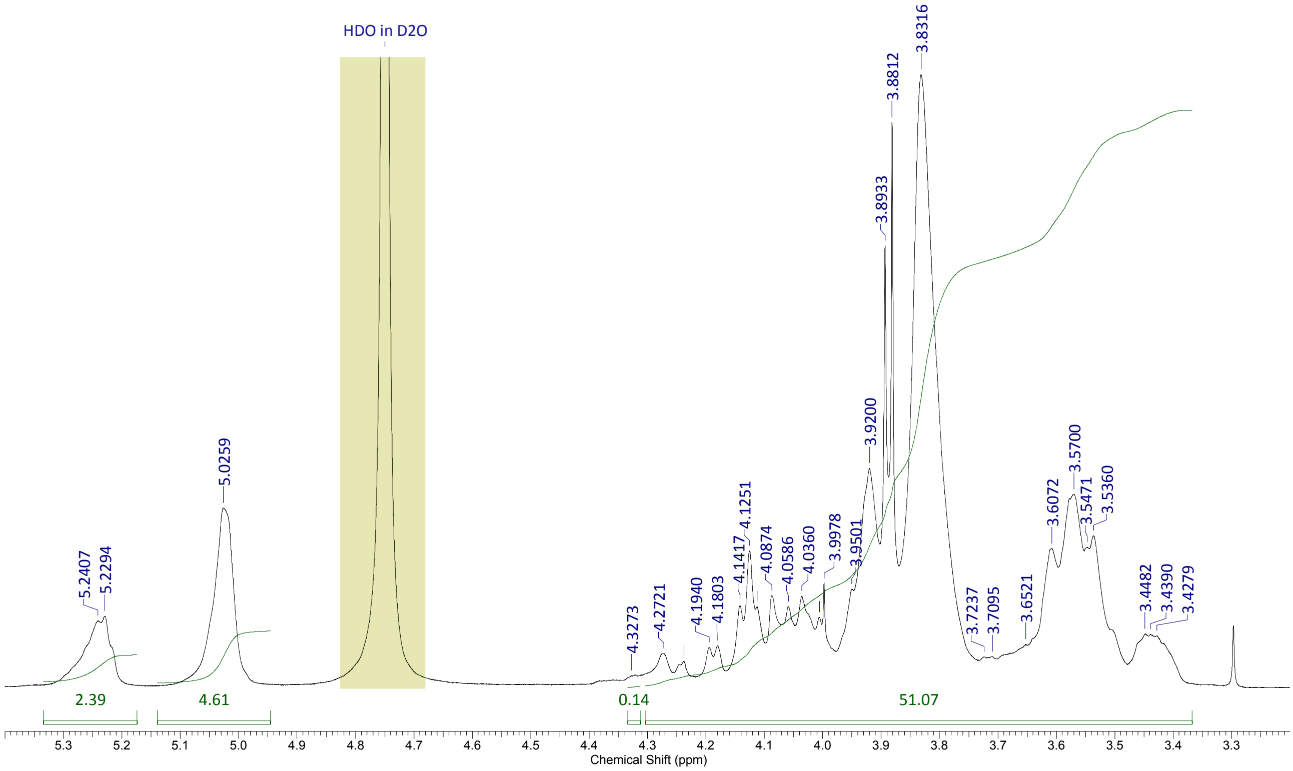

*Figure S 8: Proton NMR spectrum of carboxymethylated βCD (**2'**) prepared in ball mill (item 3 in Table 1), DS ≈4.4-4.6*

Acquisition Time (sec)	(0.0427, 0.0051)	***Comment***	5 mm BBO 1H-BB Z-GRD Z8284/0059			***Date***	03 Oct 2016 09:51:00
File Name	D:\Docs\!!Notebooks\BM_Reactions\NMR\CMBCD_LJ02\2\ser			***Frequency (MHz)***	(300.13, 75.47)	***Nucleus***	(1H, 13C)
Number of Transients	32	***Origin***	spect	***Original Points Count***	(128, 64)	***Owner***	psm
Points Count	(512, 256)	***Pulse Sequence***	hsqcedetgp	***Solvent***	DMSO	***Spectrum Type***	HSQC-DEPT
Sweep Width (Hz)	(2991.75, 12451.17)	***Temperature (degree C)***	21.760	***Title***	CBCD_LJ02 (20mg in 0.6ml D2O)_HSQC_03102016_2032		
AutoPhase	Ambiguous Phase						

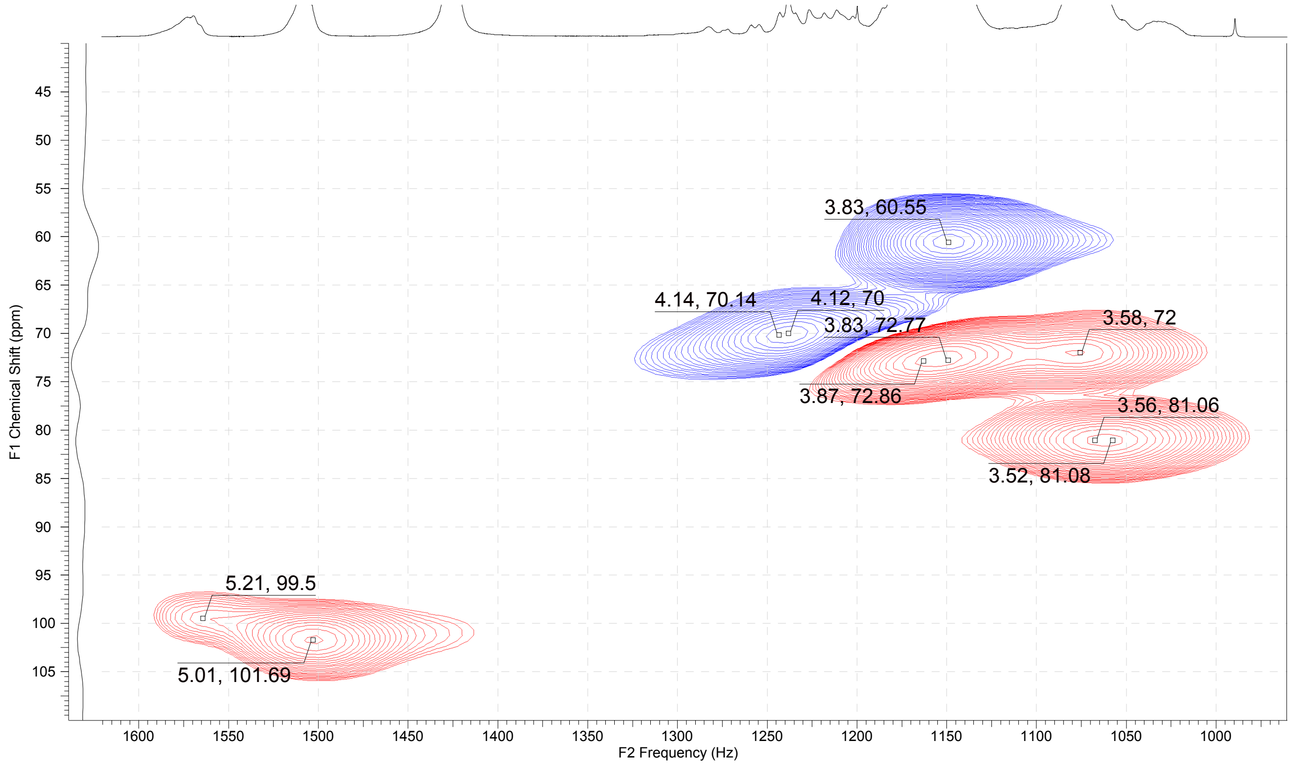

*Figure S 9: HSQC-DEPT spectrum of carboxymethylated βCD (**2'**) prepared in ball mill (item 3 in Table 1), DS ≈4.4-4.6*

Acquisition Time (sec)	3.6438	Comment	CMBCD-LJ03_1H_D2O_041016_2036_RG=50	Date	04 Oct 2016 15:34:56		
Date Stamp	04 Oct 2016 15:34:56	File Name	D:\Docs\!!Notebooks\BM_Reactions\NMR\CMBCD_LJ03\1\fid				
Frequency (MHz)	300.13	Nucleus	1H	Number of Transients	64	Origin	spect	Original Points Count	16384
Owner	root	Points Count	131072	Pulse Sequence	zg	Receiver Gain	50.80	SW(cyclical) (Hz)	4496.40
Solvent	CDCl3	Spectrum Offset (Hz)	1095.7936	Spectrum Type	STANDARD	Sweep Width (Hz)	4496.37	Temperature (degree C)	22.660

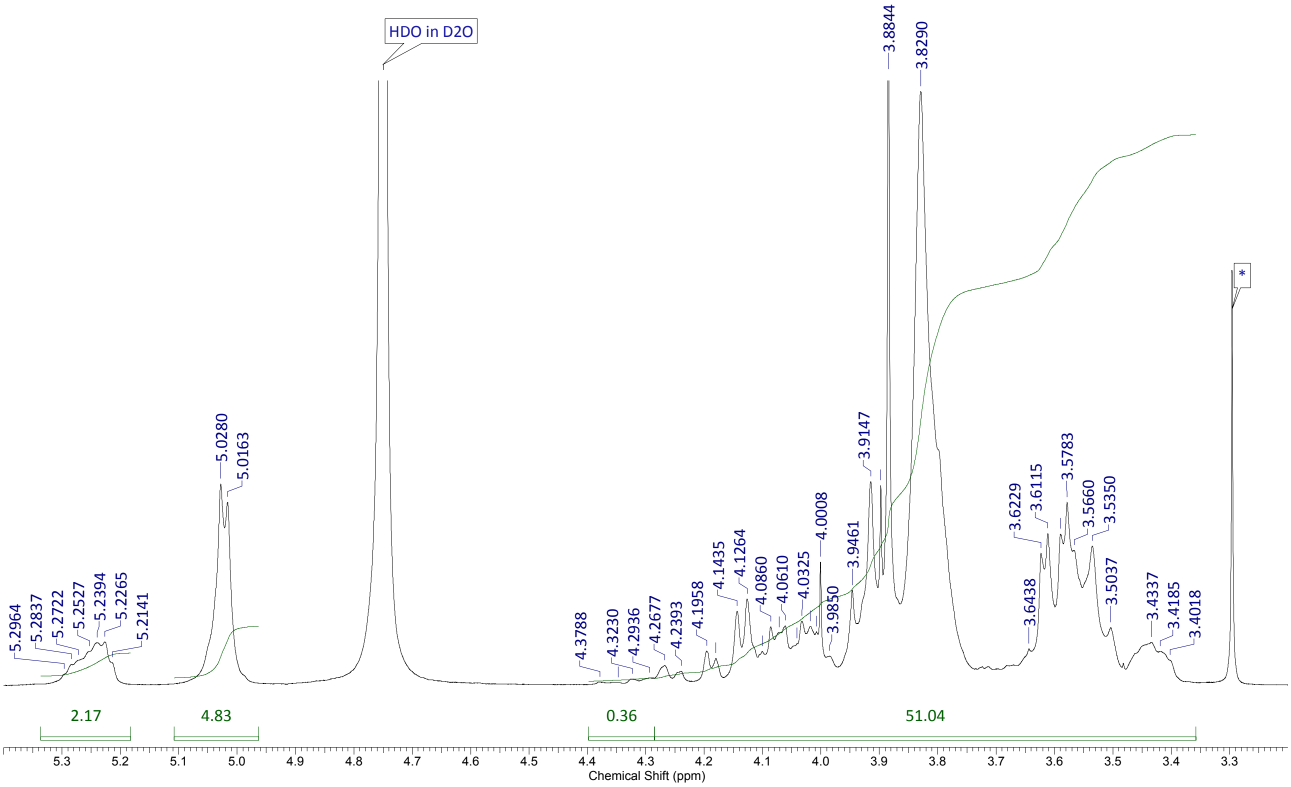

*Figure S 10: Proton NMR spectrum of carboxymethylated βCD (**2'**) prepared in ball mill (item 4 in Table 1), DS ≈4.4-4.6*

Acquisition Time (sec)	(0.0427, 0.0051)	Comment	5 mm BBO 1H-BB Z-GRD Z8284/0059	Date	04 Oct 2016 16:28:22		
File Name	D:\Docs\!!Notebooks\BM_Reactions\NMR\CMBCD_LJ03\2\ser			Frequency (MHz)	(300.13, 75.47)	Nucleus	(1H, 13C)
Number of Transients	32	Origin	spect	Original Points Count	(128, 64)	Owner	psm
Points Count	(512, 256)	Pulse Sequence	hsqcedetgp	Solvent	DMSO	Spectrum Type	HSQC-DEPT
Sweep Width (Hz)	(2991.75, 12451.17)	Temperature (degree C)	22.760	Title	CMBCD-LJ03_HSQC_D2O_041016_2036_RG=50		

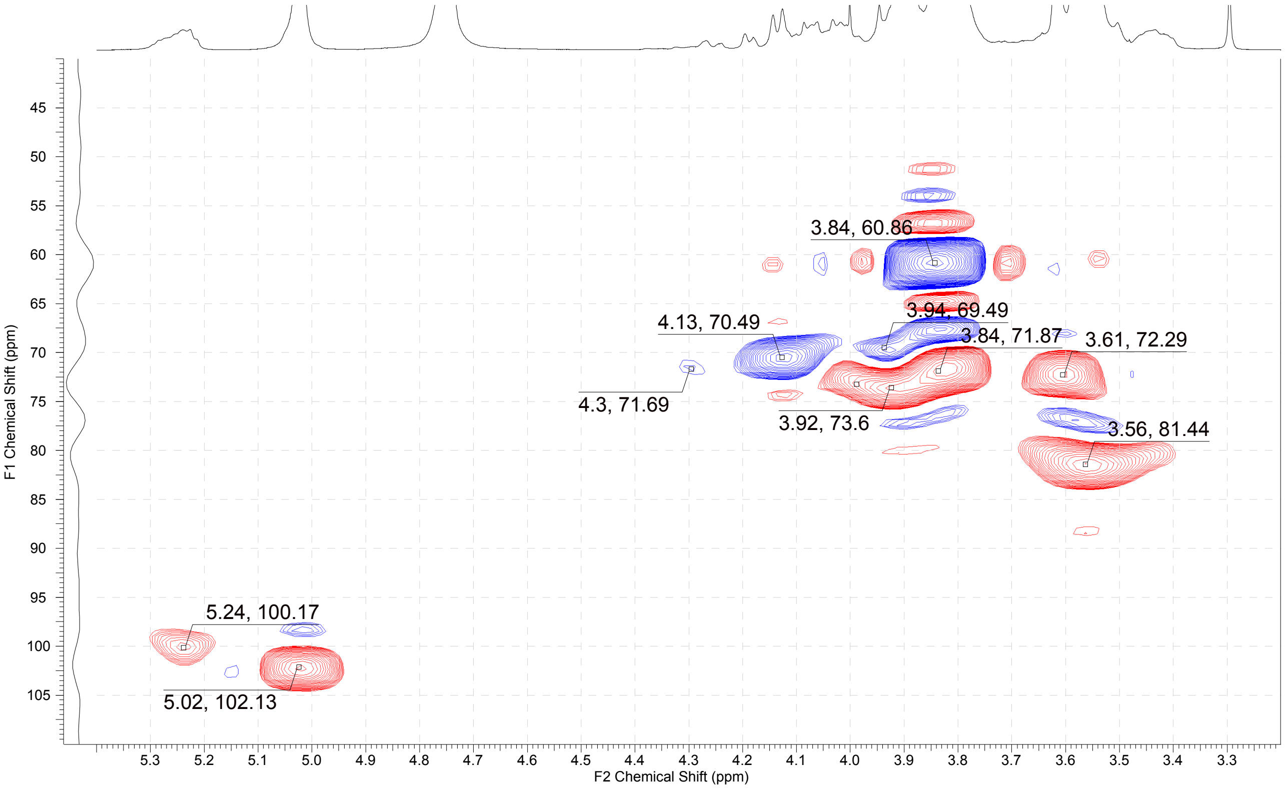

*Figure S 11: HSQC-DEPT spectrum of carboxymethylated βCD (**2'**) prepared in ball mill (item 4 in Table 1), DS ≈4.4-4.6*

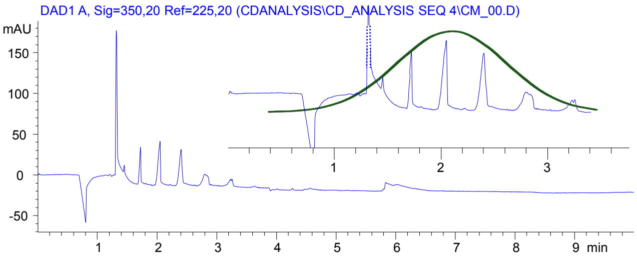

Carboxymethylated ßCD (**2**) DS 4.3-4.5, synthesized in solution(item 1 in Table 1)

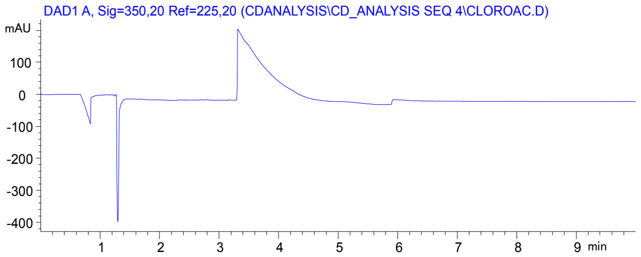

Chloroacetic acid sodium salt

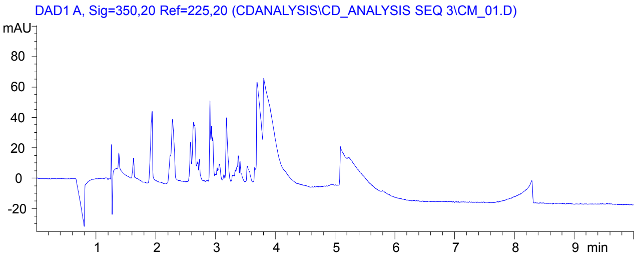

Carboxymethylated ßCD (**2**’) DS 4.3-4.6, synthesis in ball mill (item 2 in Table 1)

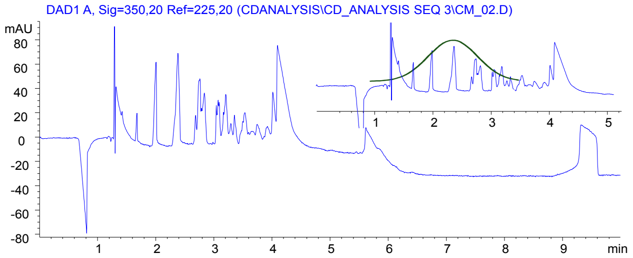

Carboxymethylated ßCD (**2**’) DS 4.4-4.6, synthesis in ball mill (item 3 in Table 1)

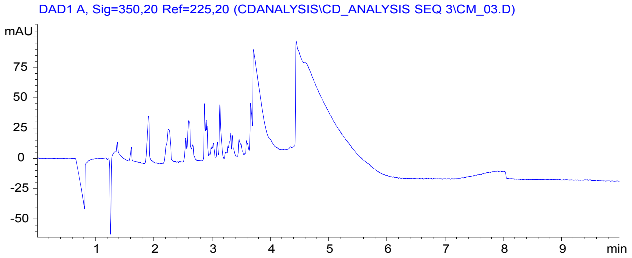

Carboxymethylated ßCD (**2**’) DS 4.4-4.6, synthesis in ball mill (item 4 in Table 1)

Figure S 12: Capillary electropherogram of carboxymethylated ßCDs

**Acquisition Time (sec)**	3.6438	**Comment**	CE_LJ00SOL_1H_D2O_131016_2057_rg=90	**Date**	13 Oct 2016 15:17:52		
**Date Stamp**	13 Oct 2016 15:17:52	**File Name**	E:\doc\!!molecules\Anal\NMR\CE_LJ00SOL\1\fid			**Frequency (MHz)**	300.13
**Nucleus**	1H	**Number of Transients**	64	**Origin**	spect	**Original Points Count**	16384
**Owner**	root	**Points Count**	262144	**Pulse Sequence**	zg	**Receiver Gain**	90.50
**SW(cyclical) (Hz)**	4496.40	**Solvent**	DEUTERIUM OXIDE	**Spectrum Offset (Hz)**	1096.8828	**Spectrum Type**	STANDARD
**Sweep Width (Hz)**	4496.39	**Temperature (degree C)**	20.160				

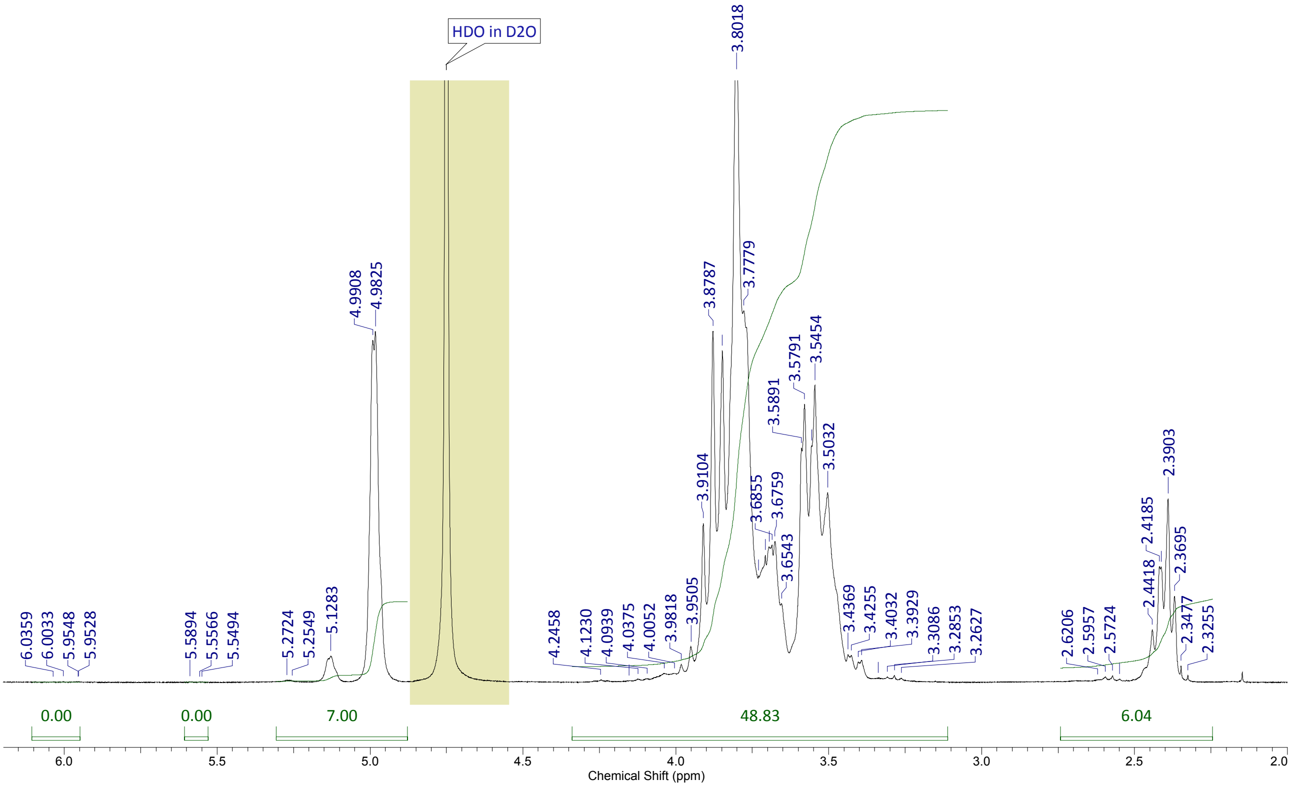

*Figure S 13: Proton NMR spectrum of carboxyethylated βCD (**3**) prepared in solution (item 8 in Table 1), DS ≈3.0-3.4*

**Acquisition Time (sec)**	(0.0427, 0.0051)	**Comment**	5 mm BBO 1H-BB Z-GRD Z8284/0059	**Date**	13 Oct 2016 16:11:42
**File Name**	D:\Docs\!!Notebooks\BM_Reactions\NMR\CE_LJ00SOL\2\ser	**Frequency (MHz)**	(300.13, 75.47)	**Nucleus**	(1H, 13C)
**Number of Transients**	32	**Origin**	spect	**Original Points Count**	(128, 64)
**Owner**	psm	**Points Count**	(1024, 512)	**Pulse Sequence**	hsqcedetgp
**Solvent**	DMSO	**Spectrum Type**	HSQC-DEPT	**Sweep Width (Hz)**	(2994.67, 12475.59)
**Temperature (degree C)**	20.260	**Title**	CE_LJ00SOL_HSQC_D2O_131016_2057_rg=90		

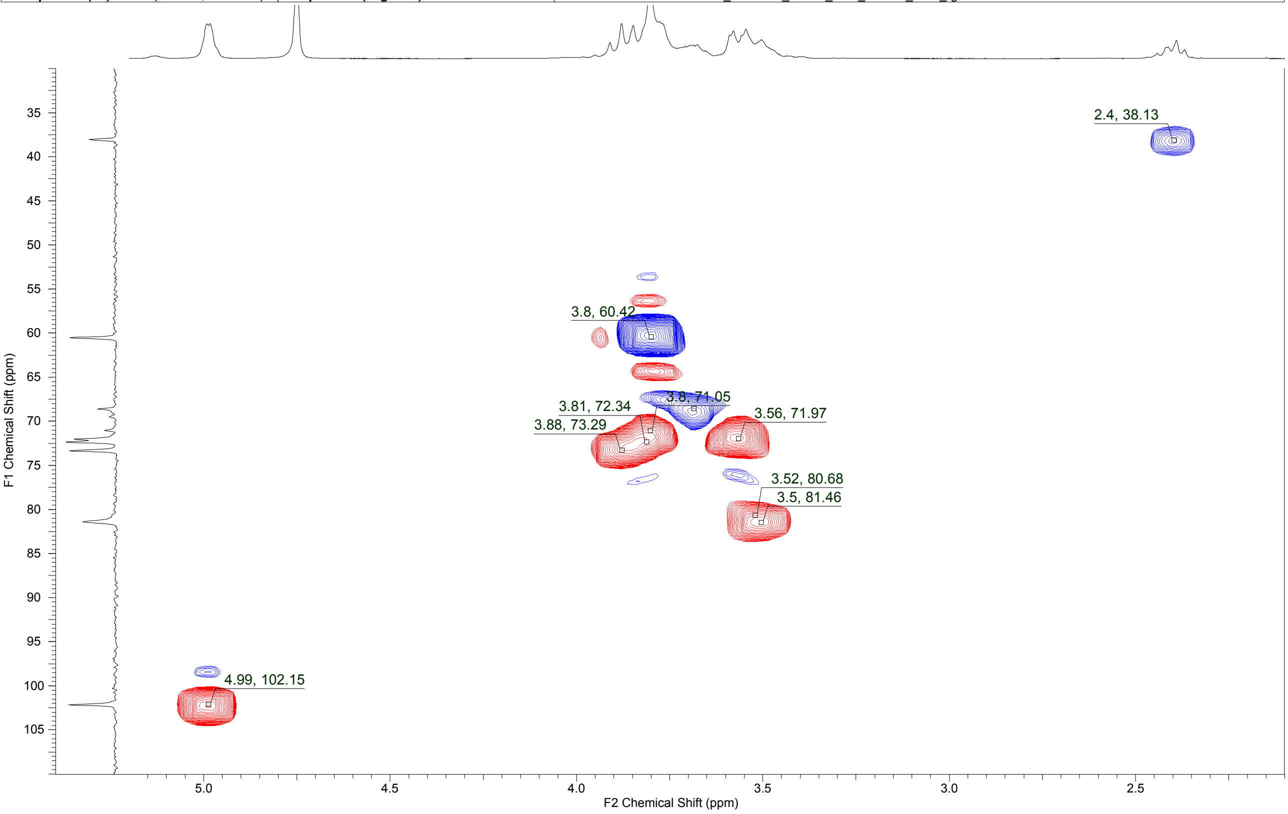

*Figure S 14: HSQC-DEPT spectrum of carboxyethylated βCD (**3**) prepared in solution (item 8 in Table 1), DS ≈3.0-3.4*

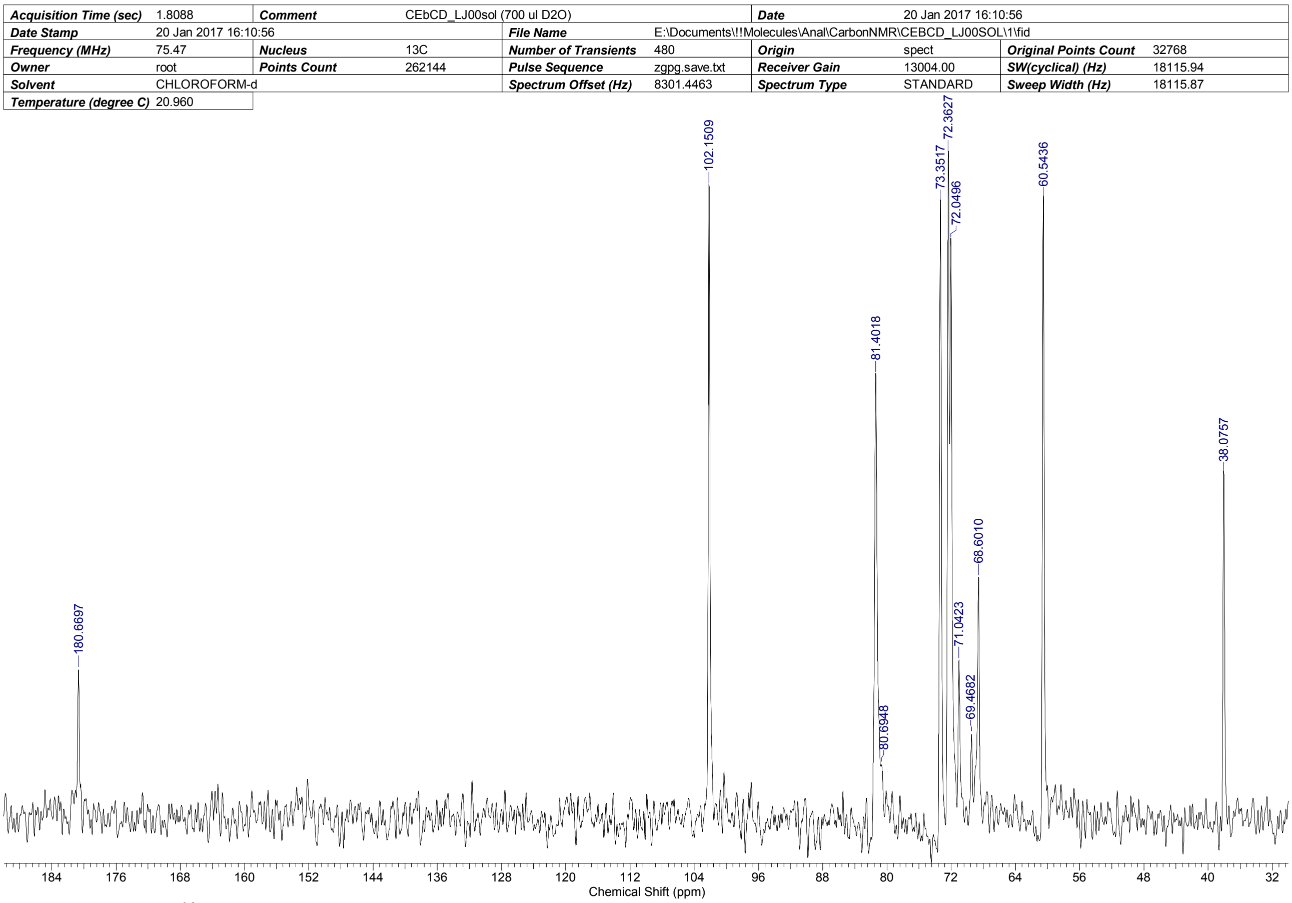

*Figure S 15: ^{13}C-NMR spectrum of carboxyethylated ßCD (**3**) prepared in solution (item 8 in Table 1), DS ≈3.0-3.4*

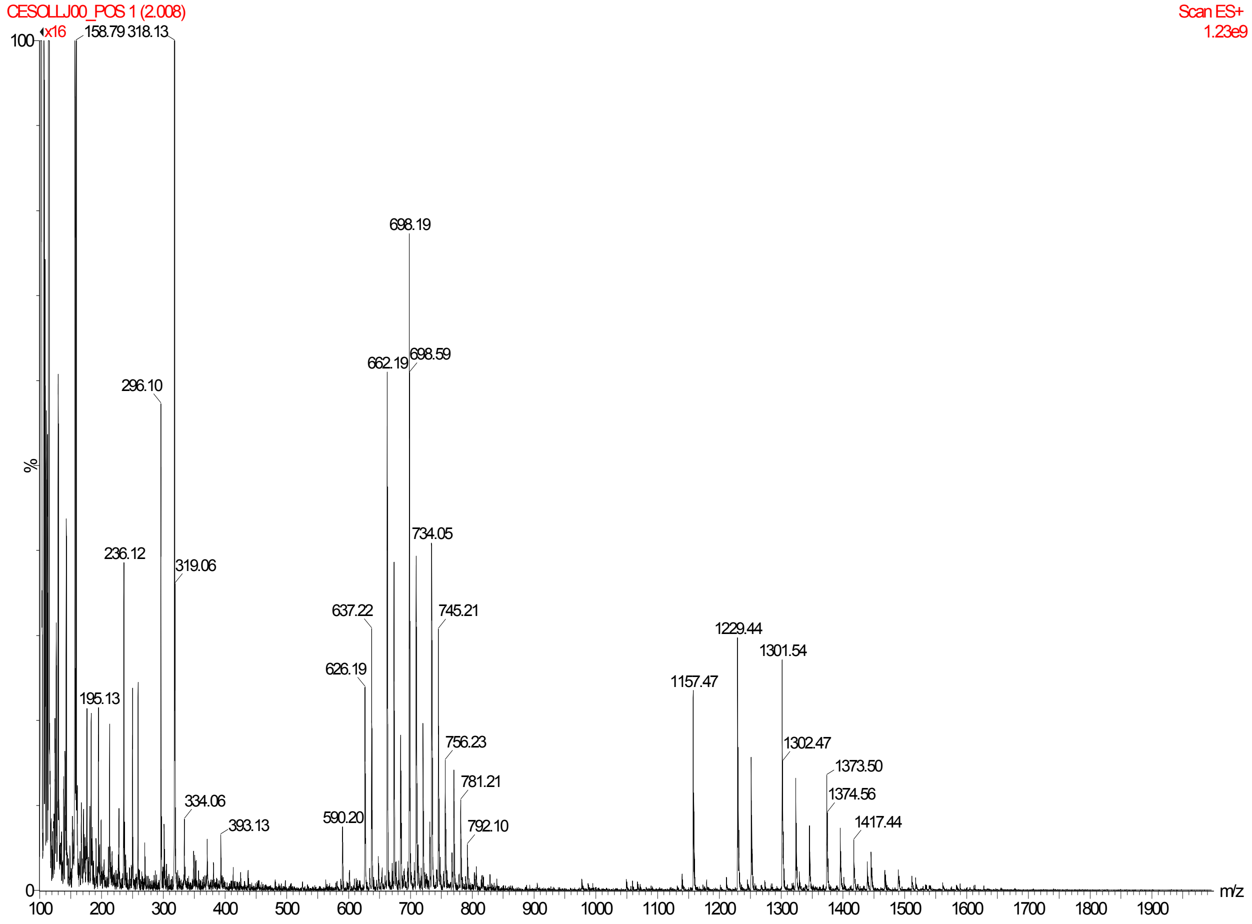

*Figure S 16: ESI+ mass spectrum of carboxyethylated ßCD (**3**) prepared in solution (item 8 in Table 1), DS ≈3.0-3.4*

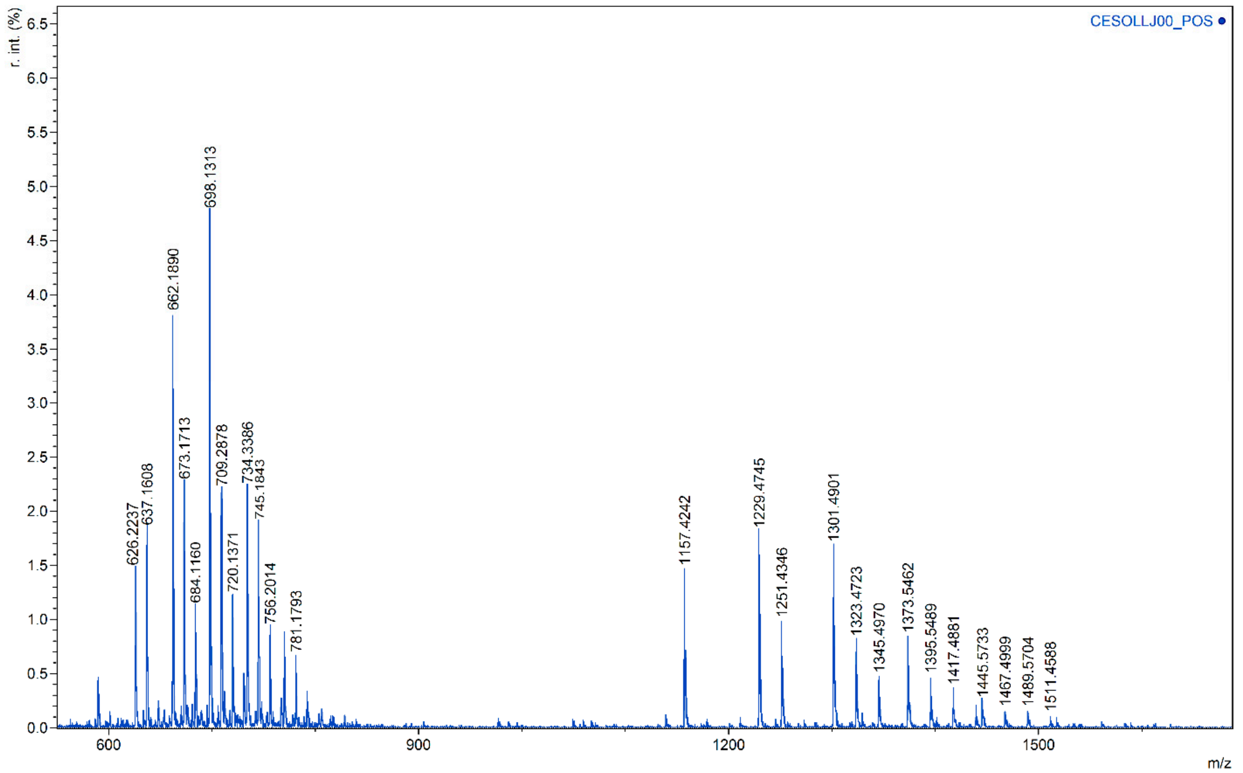

Meas. m/z	Calc. m/z	δ (Da)	Z	Annotation	Formula
626.2237	626.1847	0.0390	2	(+1 CH2CH2COOH)/2	(C42H70O35Na2)1(CH1CH2COOH)1(CH1CH2COONa)0
637.1608	637.1756	-0.0148	2	(+1 CH2CH2COONa)/2	(C42H70O35Na2)1(CH1CH2COOH)0(CH1CH2COONa)1
662.1890	662.1952	-0.0063	2	(+2 CH2CH2COOH)/2	(C42H70O35Na2)1(CH1CH2COOH)2(CH1CH2COONa)0
673.1713	673.1862	-0.0149	2	(+1 CH2CH2COOH +1 CH2CH2COONa)/2	(C42H70O35Na2)1(CH1CH2COOH)1(CH1CH2COONa)1
684.1160	684.1772	-0.0611	2	(+2 CH2CH2COONa)/2	(C42H70O35Na2)1(CH1CH2COOH)0(CH1CH2COONa)2
698.1313	698.2058	-0.0745	2	(+3 CH2CH2COOH)/2	(C42H70O35Na2)1(CH1CH2COOH)3(CH1CH2COONa)0
709.2878	709.1968	0.0911	2	(+2 CH2CH2COOH +1 CH2CH2COONa)/2	(C42H70O35Na2)1(CH1CH2COOH)2(CH1CH2COONa)1
720.1371	720.1877	-0.0507	2	(+1 CH2CH2COOH +2 CH2CH2COONa)/2	(C42H70O35Na2)1(CH1CH2COOH)1(CH1CH2COONa)2
734.3386	734.2164	0.1223	2	(+4 CH2CH2COOH)/2	(C42H70O35Na2)1(CH1CH2COOH)4(CH1CH2COONa)0
745.1843	745.2073	-0.0230	2	(+3 CH2CH2COOH +1 CH2CH2COONa)/2	(C42H70O35Na2)1(CH1CH2COOH)3(CH1CH2COONa)1
756.2014	756.1983	0.0031	2	(+2 CH2CH2COOH +2 CH2CH2COONa)/2	(C42H70O35Na2)1(CH1CH2COOH)2(CH1CH2COONa)2
770.2052	770.2269	-0.0217	2	+5 CH2CH2COOH	(C42H70O35Na2)1(CH1CH2COOH)5(CH1CH2COONa)0
781.1793	781.2179	-0.0386	2	(+4 CH2CH2COOH +1 CH2CH2COONa)/2	(C42H70O35Na2)1(CH1CH2COOH)4(CH1CH2COONa)1
792.1803	792.2089	-0.0286	2	(+3 CH2CH2COOH +2 CH2CH2COONa)/2	(C42H70O35Na2)1(CH1CH2COOH)3(CH1CH2COONa)2
1157.4242	1157.3590	0.0652	1	C42H70O35 (βCD)	C42H70O35Na
1229.4745	1229.3801	0.0944	1	+1 CH2CH2COOH	(C42H70O35Na)1(CH1CH2COOH)1(CH1CH2COONa)0
1251.4346	1251.3621	0.0726	1	+1 CH2CH2COONa	(C42H70O35Na)1(CH1CH2COOH)0(CH1CH2COONa)1
1301.4901	1301.4012	0.0888	1	+2 CH2CH2COOH	(C42H70O35Na)1(CH1CH2COOH)2(CH1CH2COONa)0
1323.4723	1323.3832	0.0891	1	+1 CH2CH2COOH + 1 CH2CH2COONa	(C42H70O35Na)1(CH1CH2COOH)1(CH1CH2COONa)1
1345.4970	1345.3651	0.1319	1	+2 CH2CH2COONa	(C42H70O35Na)1(CH1CH2COOH)0(CH1CH2COONa)2
1373.5462	1373.4224	0.1239	1	+3 CH2CH2COOH	(C42H70O35Na)1(CH1CH2COOH)3(CH1CH2COONa)0
1395.5489	1395.4043	0.1446	1	+2 CH2CH2COOH + 1 CH2Ch2COONa	(C42H70O35Na)1(CH1CH2COOH)2(CH1CH2COONa)1
1417.4881	1417.3863	0.1019	1	+1 CH2CH2COOH + 2 CH2Ch2COONa	(C42H70O35Na)1(CH1CH2COOH)1(CH1CH2COONa)2
1445.5733	1445.4435	0.1298	1	+4 CH2CH2COOH	(C42H70O35Na)1(CH1CH2COOH)4(CH1CH2COONa)0
1467.4999	1467.4254	0.0744	1	+3 CH2CH2COOH + 1 CH2CH2COONa	(C42H70O35Na)1(CH1CH2COOH)3(CH1CH2COONa)1
1489.5704	1489.4074	0.1630	1	+2 CH2CH2COOH + 2 CH2CH2COONa	(C42H70O35Na)1(CH1CH2COOH)2(CH1CH2COONa)2
1511.4588	1511.3893	0.0694	1	+1 CH2CH2COOH + 3 CH2CH2COONa	(C42H70O35Na)1(CH1CH2COOH)1(CH1CH2COONa)3

*Figure S 17: Peak identification in ESI+ mass spectrum of carboxyethylated βCD (**3**) prepared in solution (item 8 in Table 1), DS ≈3.0-3.4*

**Acquisition Time (sec)**	3.6438	**Comment**	CE_LJ01A_1H_D2O_281016_2081_rg=80			**Date**	28 Oct 2016 11:12:32
**Date Stamp**	28 Oct 2016 11:12:32			**File Name**	E:\doc\!!molecules\Anal\NMR\CE_LJ01\3\fid	**Frequency (MHz)**	300.13
**Nucleus**	1H	**Number of Transients**	64	**Origin**	spect	**Original Points Count**	16384
**Owner**	root						
**Points Count**	524288	**Pulse Sequence**	zg	**Receiver Gain**	80.60	**SW(cyclical) (Hz)**	4496.40
**Solvent**	DEUTERIUM OXIDE						
**Spectrum Offset (Hz)**	1096.4756	**Spectrum Type**	STANDARD	**Sweep Width (Hz)**	4496.39	**Temperature (degree C)**	20.660

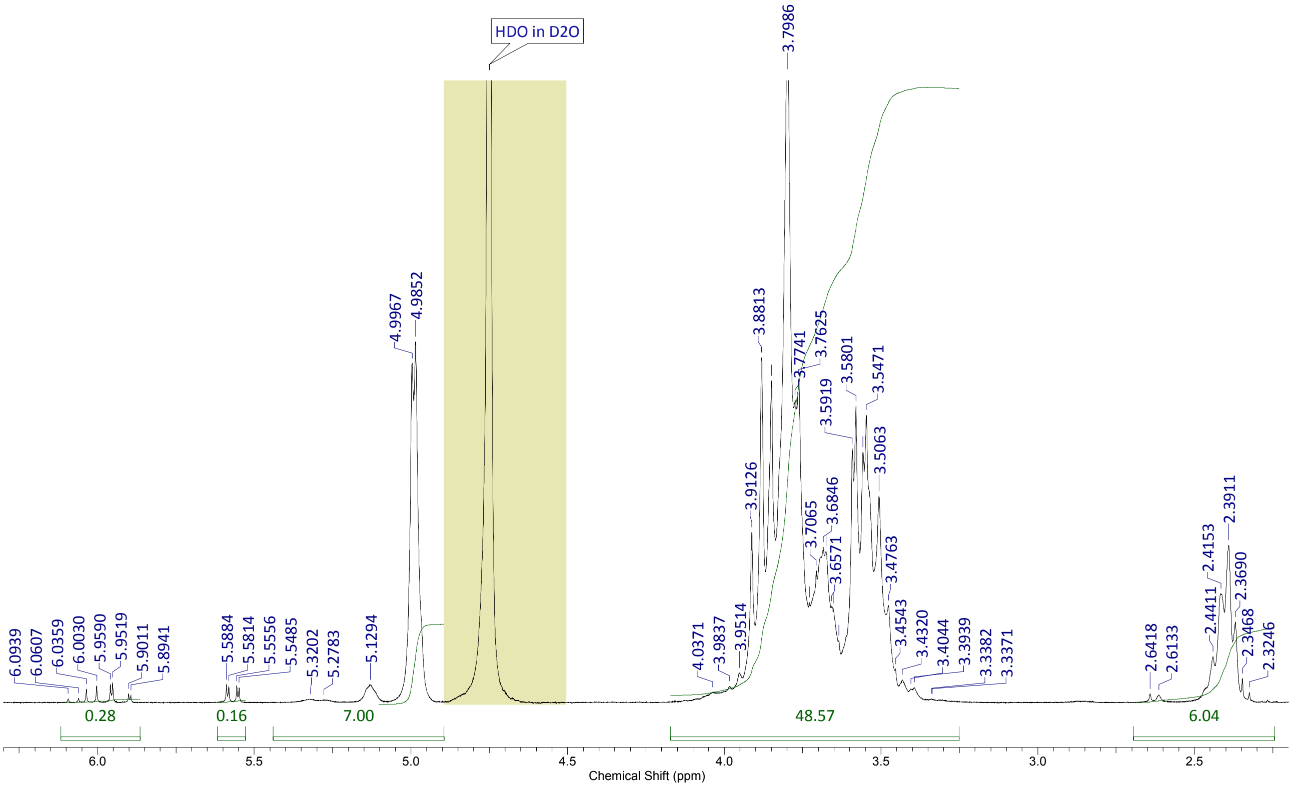

*Figure S 18: Proton NMR spectrum of carboxyethylated βCD (**3'**) prepared in ball mill (item 5 in Table 1), DS ≈3.0-3.3*

**Acquisition Time (sec)**	(0.0427, 0.0051)	**Comment**	5 mm BBO 1H-BB Z-GRD Z8284/0059			**Date**	28 Oct 2016 11:06:14
**File Name**	E:\doc\!!molecules\Anal\NMR\CE_LJ01\4\ser			**Frequency (MHz)**	(300.13, 75.47)	**Nucleus**	(1H, 13C)
**Number of Transients**	32	**Origin**	spect	**Original Points Count**	(128, 64)	**Owner**	psm
**Points Count**	(4096, 128)	**Pulse Sequence**	hsqcedetgp	**Solvent**	DMSO	**Spectrum Type**	HSQC-DEPT
**Sweep Width (Hz)**	(2996.87, 12402.34)			**Temperature (degree C)**	20.660	**Title**	CE_LJ01A_HSQC_D2O_281016_2081_rg=80

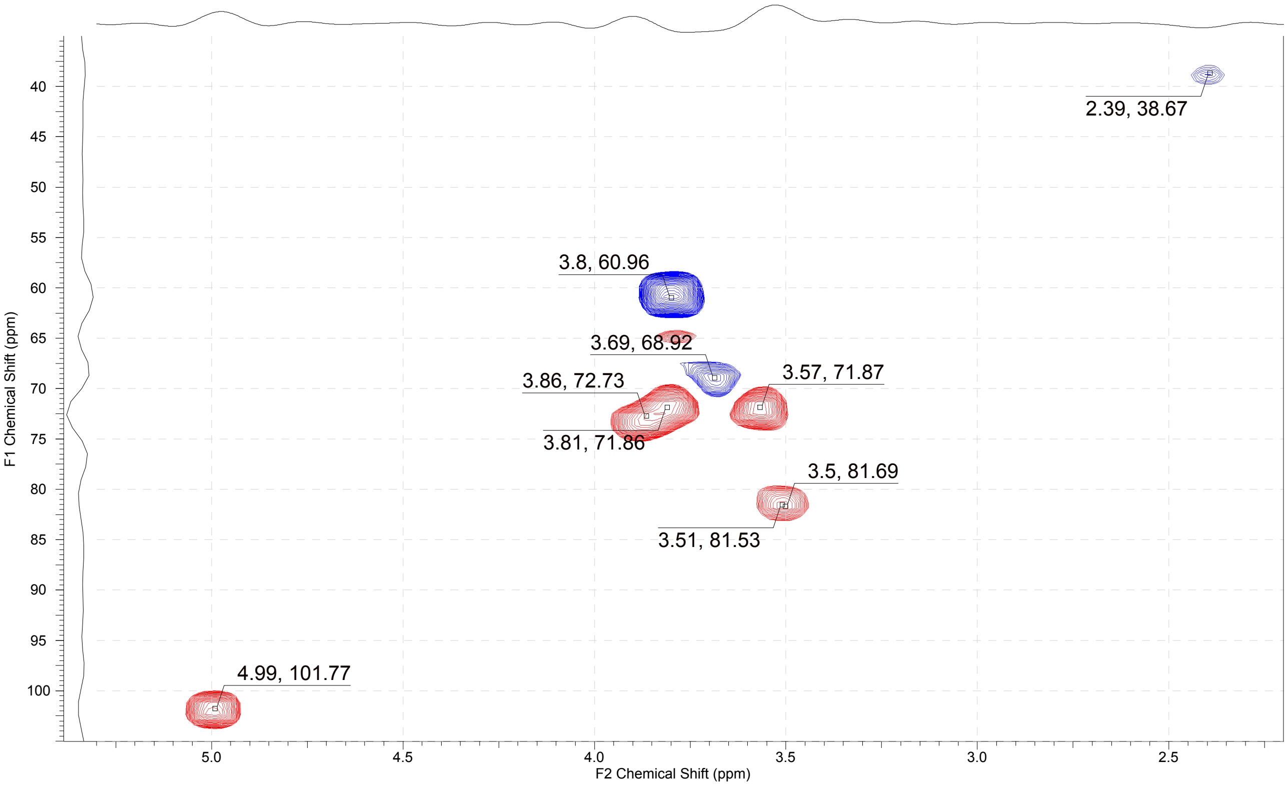

*Figure S 19: HSQC-DEPT spectrum of carboxyethylated βCD (**3'**) prepared in ball mill (item 5 in Table 1), DS ≈3.0-3.3*

**Acquisition Time (sec)**	3.6438	**Comment**	CE_LJ02A_1H_D2O_261016_2079_rg=80			**Date**	26 Oct 2016 11:46:40		
**Date Stamp**	26 Oct 2016 11:46:40			**File Name**	E:\doc\!!molecules\Anal\NMR\CE_LJ02\3\fid			**Frequency (MHz)**	300.13
**Nucleus**	1H	**Number of Transients**	64	**Origin**	spect	**Original Points Count**	16384	**Owner**	root
**Points Count**	524288	**Pulse Sequence**	zg	**Receiver Gain**	90.50	**SW(cyclical) (Hz)**	4496.40	**Solvent**	DEUTERIUM OXIDE
**Spectrum Offset (Hz)**	1096.3091	**Spectrum Type**	STANDARD	**Sweep Width (Hz)**	4496.39	**Temperature (degree C)**	20.460		

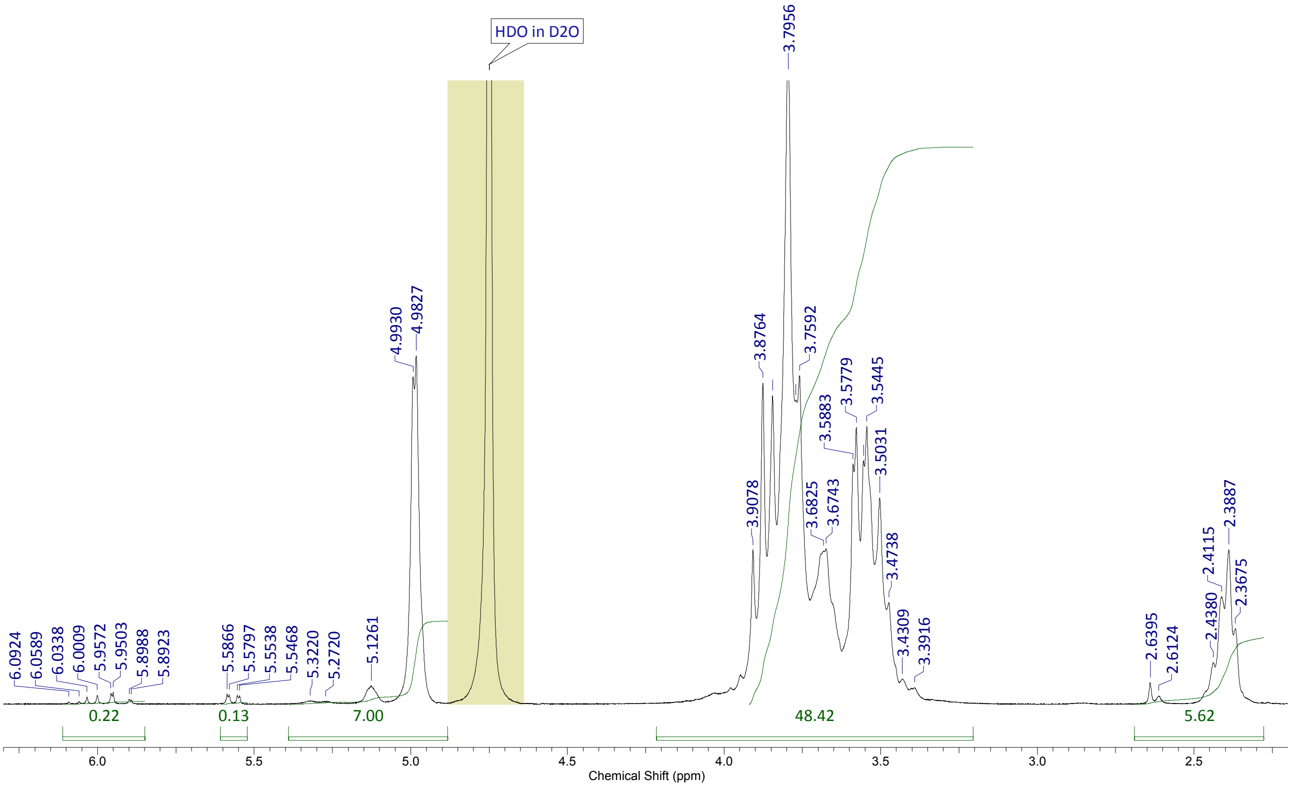

*Figure S 20: Proton NMR spectrum of carboxyethylated ßCD (**3'**) prepared in ball mill (item 6 in Table 1), DS ≈2.8-3.2*

**Acquisition Time (sec)**	(0.0427, 0.0051)	**Comment**	5 mm BBO 1H-BB Z-GRD Z8284/0059			**Date**	26 Oct 2016 11:40:52
**File Name**	E:\doc\!!molecules\Anal\NMR\CE_LJ02\4\ser			**Frequency (MHz)**	(300.13, 75.47)	**Nucleus**	(1H, 13C)
**Number of Transients**	32	**Origin**	spect	**Original Points Count**	(128, 64)	**Owner**	psm
**Points Count**	(256, 512)	**Pulse Sequence**	hsqcedetgp	**Solvent**	DMSO	**Spectrum Type**	HSQC-DEPT
**Sweep Width (Hz)**	(2985.89, 12475.59)			**Temperature (degree C)**	20.560	**Title**	CE_LJ02A_HSQC_D2O_261016_2079_rg=80

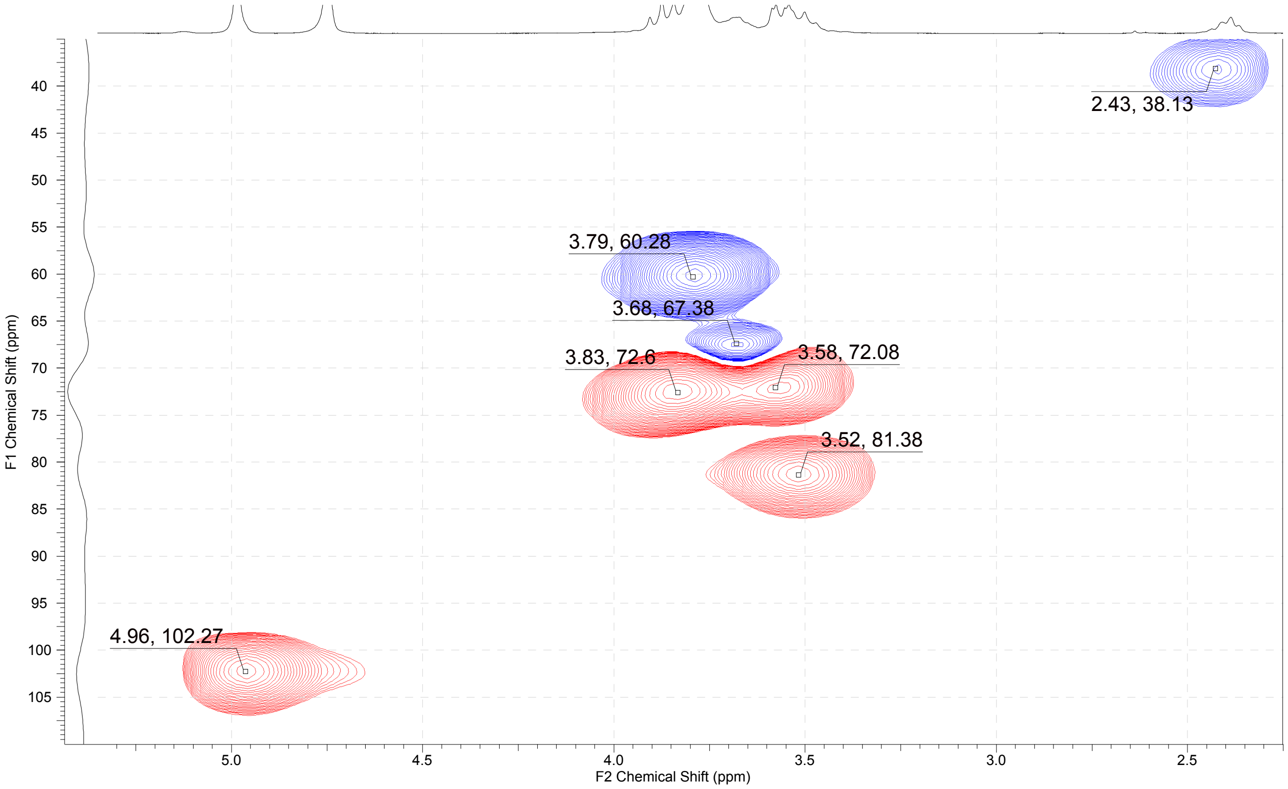

*Figure S 21: HSQC-DEPT spectrum of carboxyethylated ßCD (**3'**) prepared in ball mill (item 6 in Table 1), DS ≈2.8-3.2*

**Acquisition Time (sec)**	3.6438	**Comment**	CE_LJ03B_1H_D2O_281016_2082_rg=60			**Date**	28 Oct 2016 13:41:52
**Date Stamp**	28 Oct 2016 13:41:52			**File Name**	E:\doc\!!molecules\Anal\NMR\CE_LJ03\3\fid	**Frequency (MHz)**	300.13
**Nucleus**	1H	**Number of Transients**	64	**Origin**	spect	**Original Points Count**	16384
**Owner**	root	**Points Count**	524288	**Pulse Sequence**	zg	**Receiver Gain**	64.00
**SW(cyclical) (Hz)**	4496.40	**Solvent**	CDCl3	**Spectrum Offset (Hz)**	1096.4291	**Spectrum Type**	STANDARD
**Sweep Width (Hz)**	4496.39	**Temperature (degree C)**	20.660				

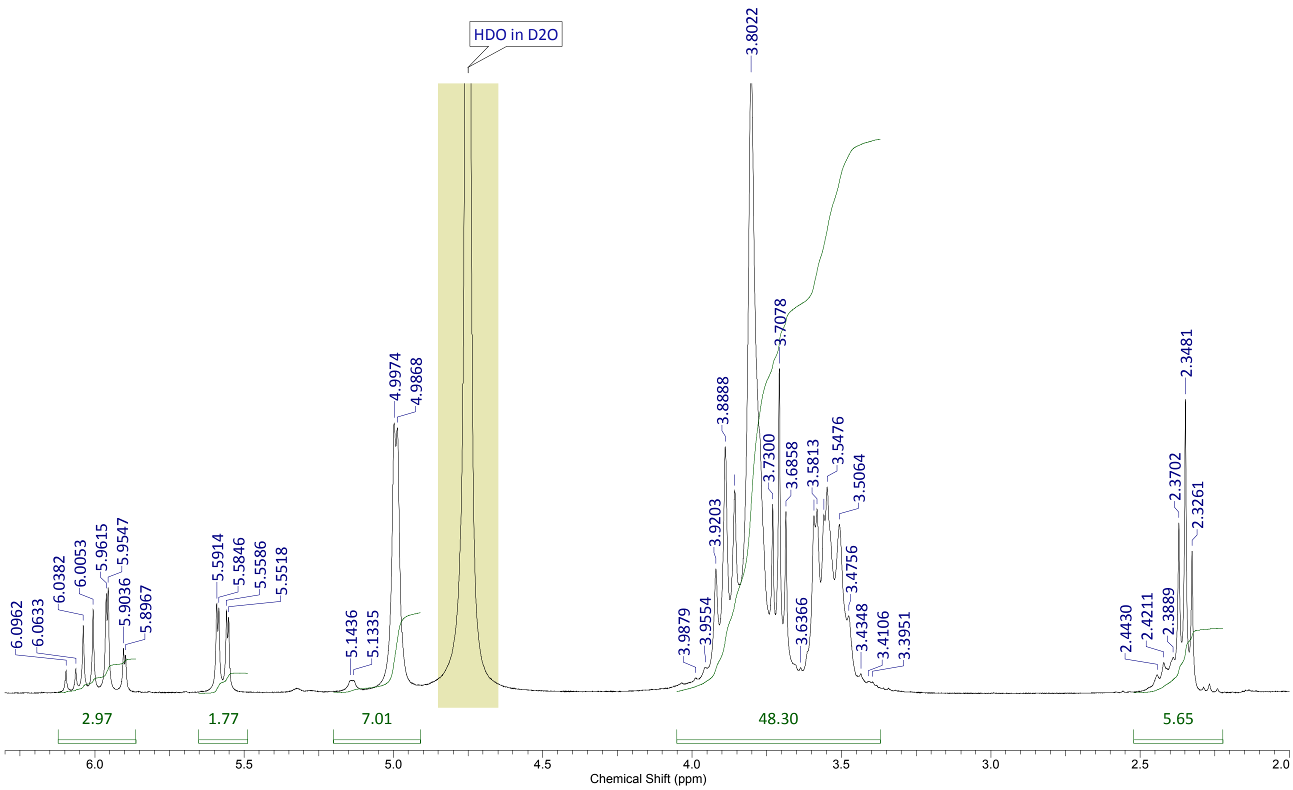

*Figure S 22: Proton NMR spectrum of carboxyethylated βCD (**3'**) prepared in ball mill (item 7 in Table 1), DS ≈2.8-3.1*

**Acquisition Time (sec)**	(0.0427, 0.0051)	**Comment**	5 mm BBO 1H-BB Z-GRD Z8284/0059			**Date**	28 Oct 2016 13:36:38
**File Name**	E:\doc\!!molecules\Anal\NMR\CE_LJ03\4\ser			**Frequency (MHz)**	(300.13, 75.47)	**Nucleus**	(1H, 13C)
**Number of Transients**	32	**Origin**	spect	**Original Points Count**	(128, 64)	**Owner**	psm
**Points Count**	(2048, 8192)	**Pulse Sequence**	hsqcedetgp	**Solvent**	DMSO	**Spectrum Type**	HSQC-DEPT
**Sweep Width (Hz)**	(2996.14, 12498.47)			**Temperature (degree C)**	20.760	**Title**	CE_LJ03B_HSQC_D2O_281016_2082_rg=60

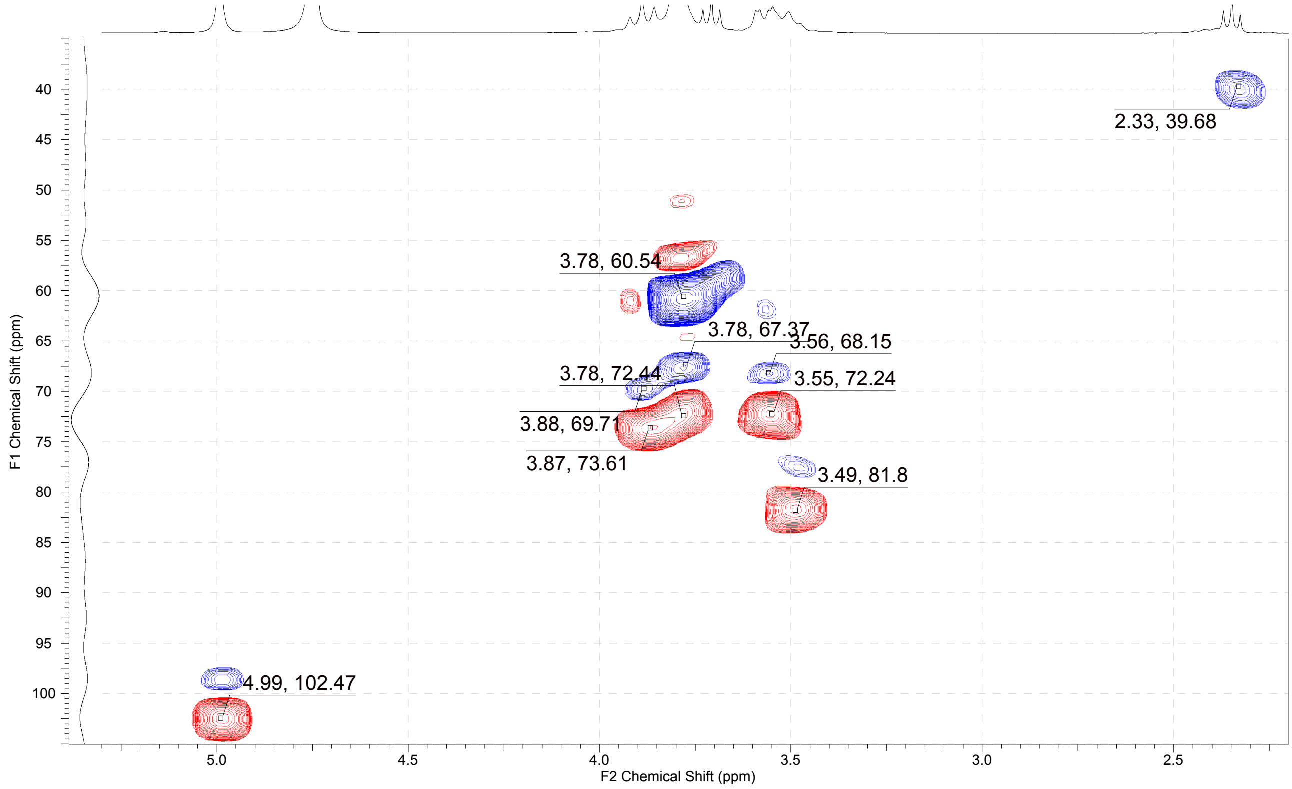

*Figure S 23: HSQC-DEPT spectrum of carboxyethylated βCD (**3'**) prepared in ball mill (item 7 in Table 1), DS ≈2.8-3.1*

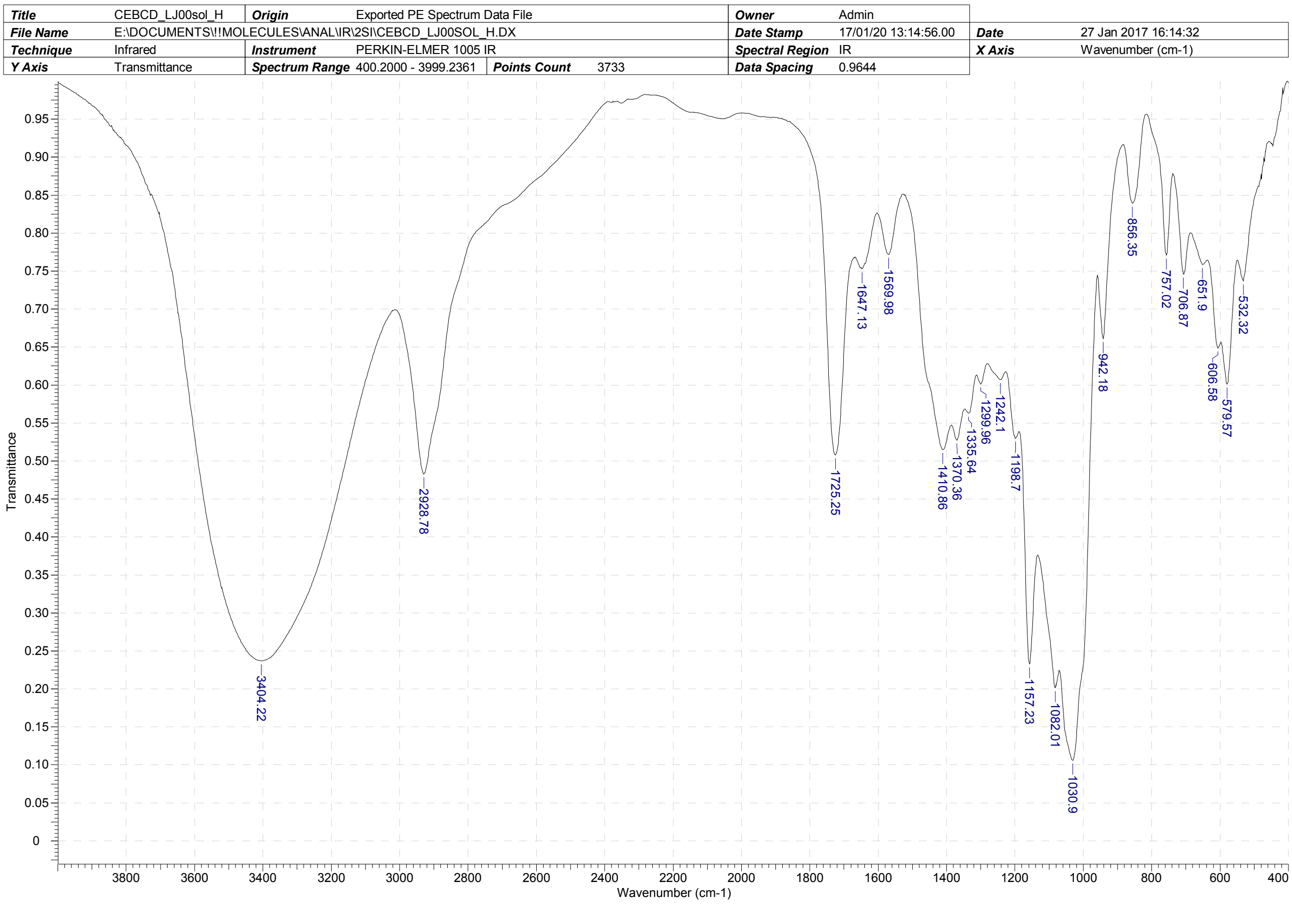

*Figure S 24: IR spectrum of carboxylethylated ßCD (**3**) prepared in solution (item 8 in Table 1) as acid, DS ≈3.0-3.4*

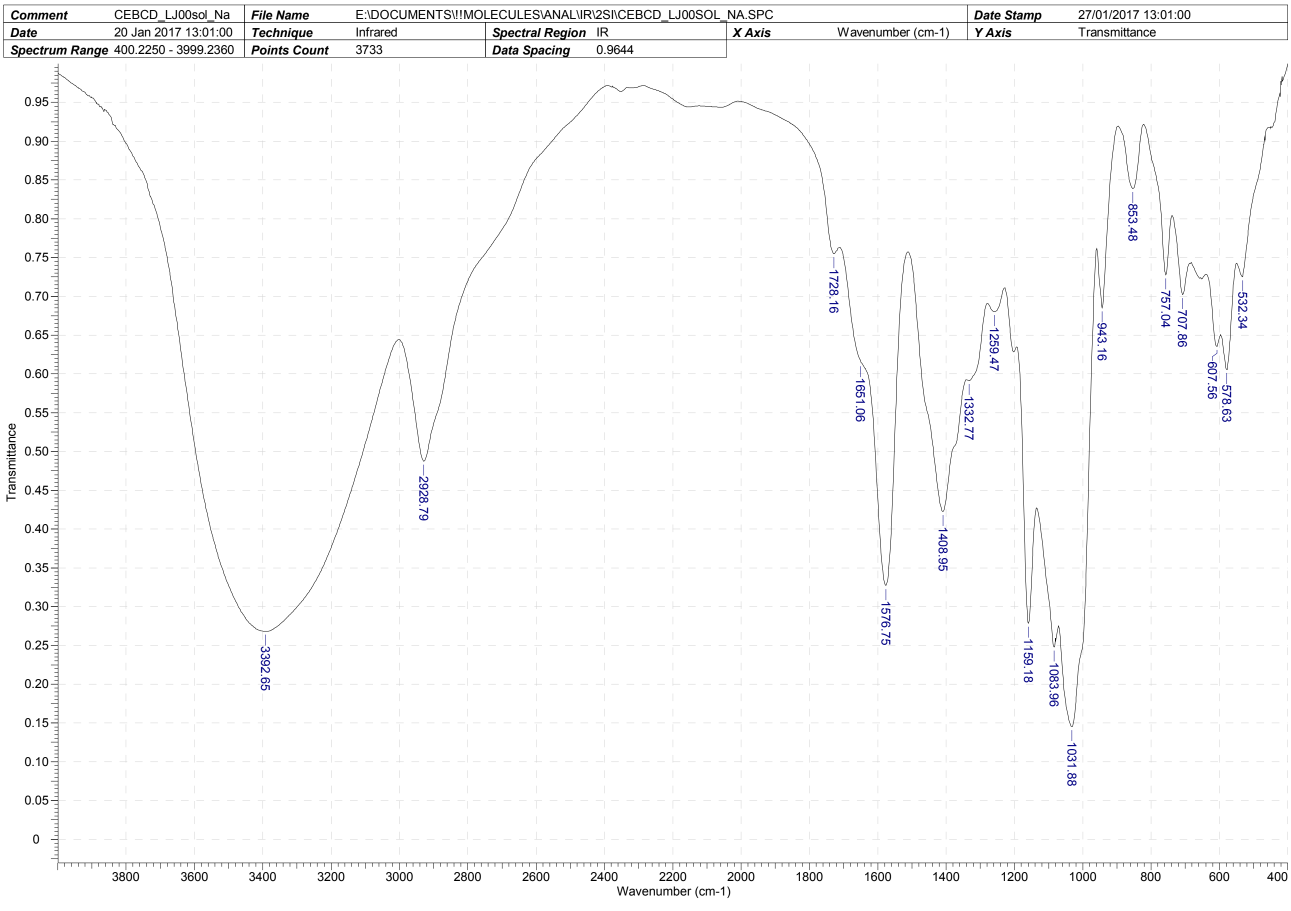

*Figure S 25: IR spectrum of carboxylethylated ßCD (**3**) prepared in solution (item 8 in Table 1) as sodium salt, DS ≈3.0-3.4*

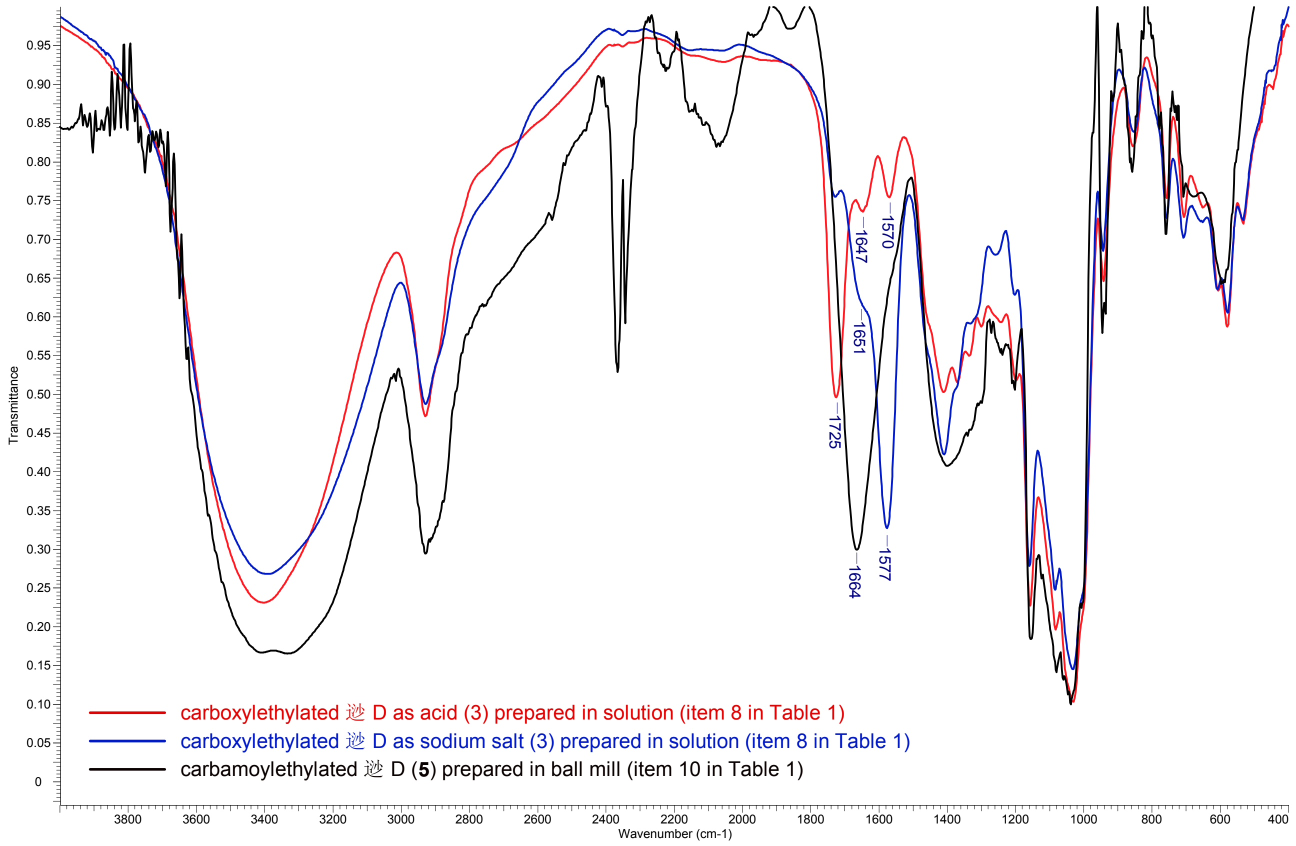

*Figure S 26: Overlay of IR spectrum of carboxylethylated ßCD acid & salt form (**3**) prepared in solution (item 8 in Table 1), DS ≈3.0-3.4 and carbamoylethylated ßCD prepared in ball mill (item 10 in table 1)*

Acquisition Time (sec)	3.6438	*Comment*	CAMBCD_LJ01w (30 mg in 0.6ml D2O)_19102016_1H_RG=180					*Date*	19 Oct 2016 11:40:16
Date Stamp	19 Oct 2016 11:40:16			*File Name*	E:\doc\!!molecules\Anal\NMR\CAMBCD_LJ01\7\fid			*Frequency (MHz)*	300.13
Nucleus	1H	*Number of Transients*	64	*Origin*	spect	*Original Points Count*	16384	*Owner*	root
Points Count	262144	*Pulse Sequence*	zg	*Receiver Gain*	181.00	*SW(cyclical) (Hz)*	4496.40	*Solvent*	CDCl3
Spectrum Offset (Hz)	1096.5278	*Spectrum Type*	STANDARD	*Sweep Width (Hz)*	4496.39	*Temperature (degree C)*	20.860		

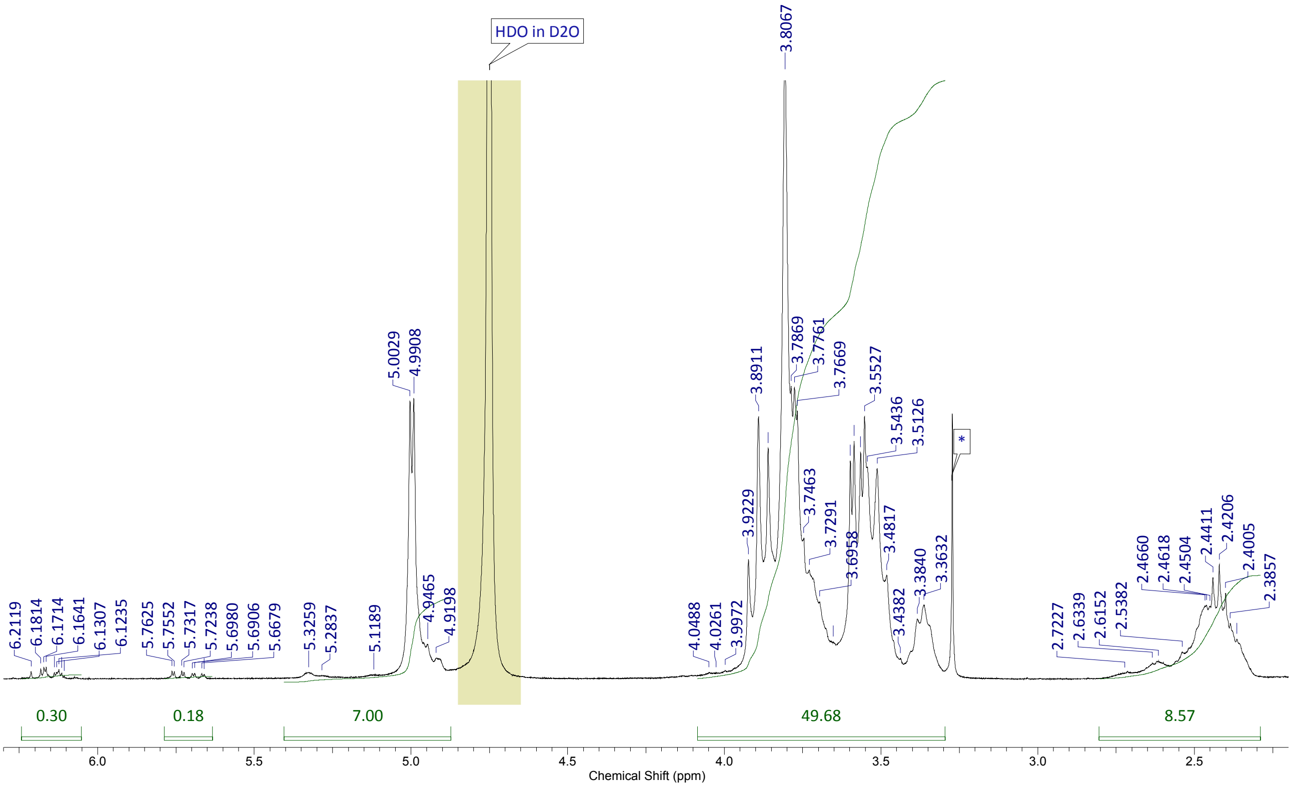

*Figure S 27: Proton NMR spectrum of carbamoylethylated βCD (**5**) prepared in ball mill (item 9 in Table 1), DS ≈3.8-4.3*

Acquisition Time (sec)	(0.0427, 0.0051)	*Comment*	5 mm BBO 1H-BB Z-GRD Z8284/0059			*Date*	19 Oct 2016 11:34:02
File Name	E:\doc\!!molecules\Anal\NMR\CAMBCD_LJ01\8\ser			*Frequency (MHz)*	(300.13, 75.47)	*Nucleus*	(1H, 13C)
Number of Transients	32	*Origin*	spect	*Original Points Count*	(128, 64)	*Owner*	psm
Points Count	(256, 256)	*Pulse Sequence*	hsqcedetgp	*Solvent*	DMSO	*Spectrum Type*	HSQC-DEPT
Sweep Width (Hz)	(2985.89, 12451.17)	*Temperature (degree C)*	20.960	*Title*	CAMBCD_LJ01w (30 mg in 0.6ml D2O)_19102016_HSQC		
AutoPhase	Ambiguous Phase						

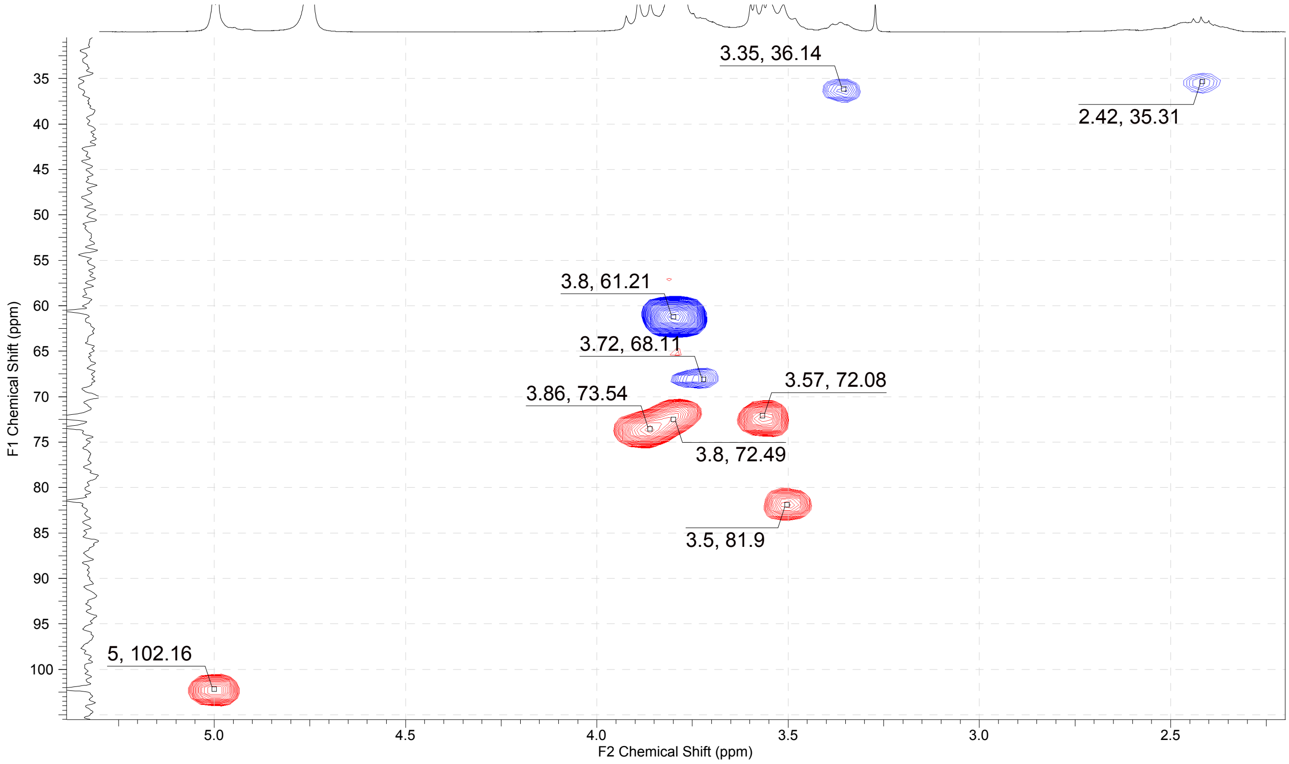

*Figure S 28: HSQC-DEPT spectrum of carbamoylethylated βCD (**5**) prepared in ball mill (item 9 in Table 1), DS ≈3.8-4.3*

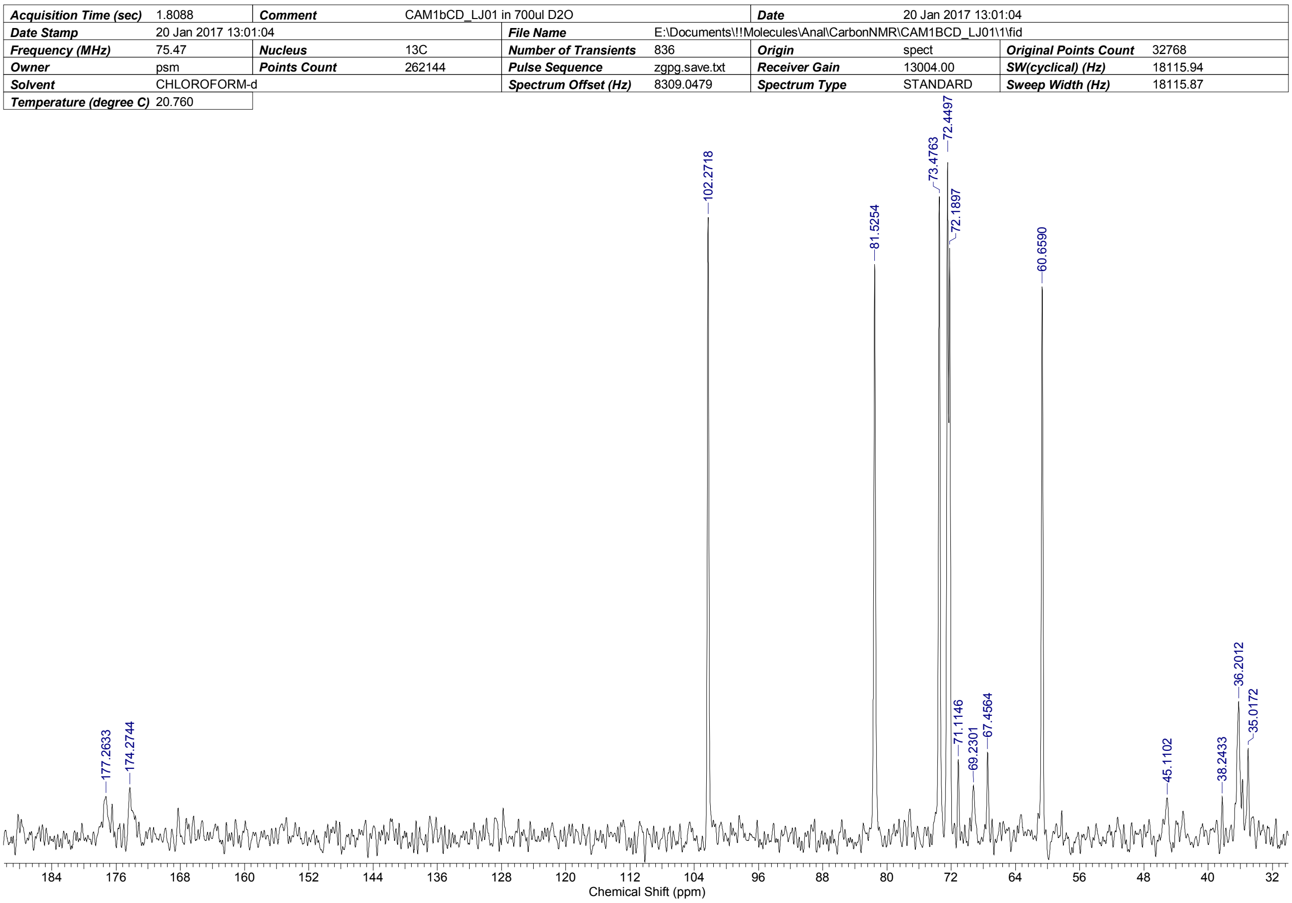

Figure S 29: ^{13}C-NMR spectrum of carbamoylethylated ßCD (**5**) prepared in ball mill (item 9 in Table 1), DS ≈3.8-4.3

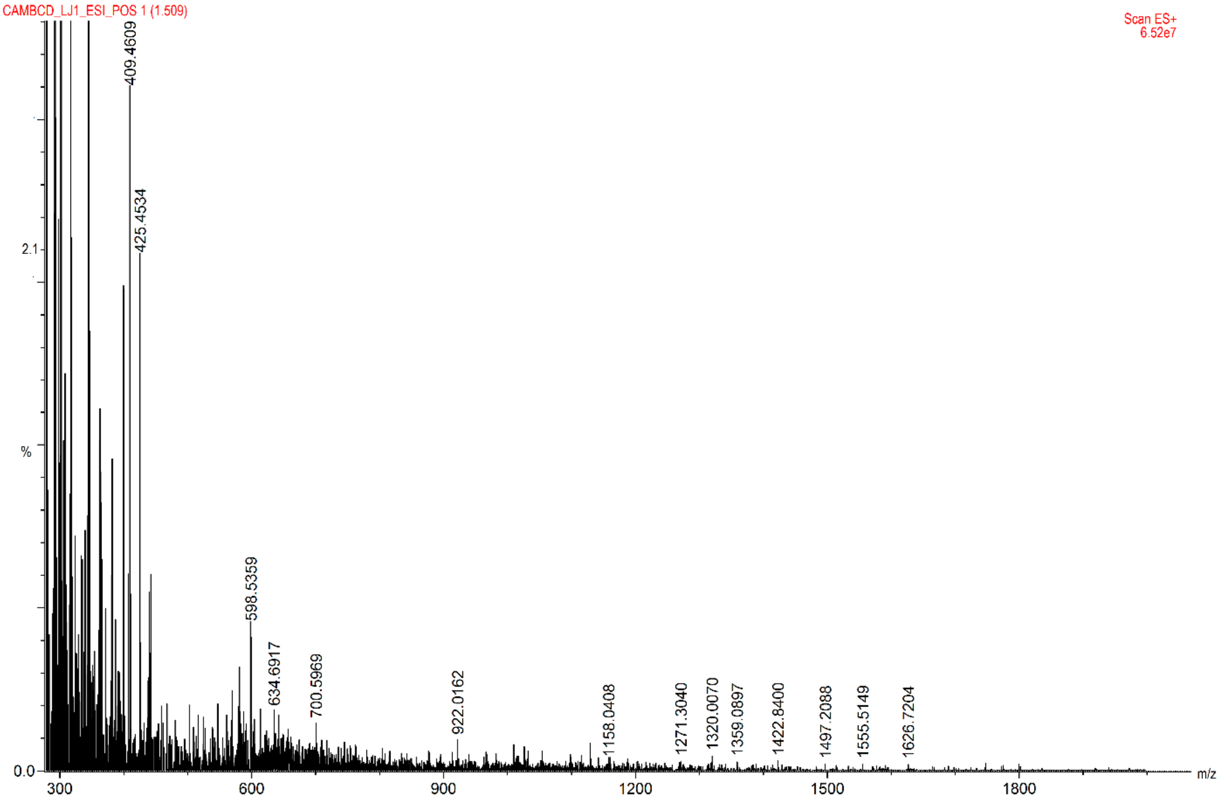

Figure S 30: ESI+ mass spectrum of carbamoylethylated ßCD (**5**) prepared in ball mill (item 9 in Table 1), DS ≈3.8-4.3

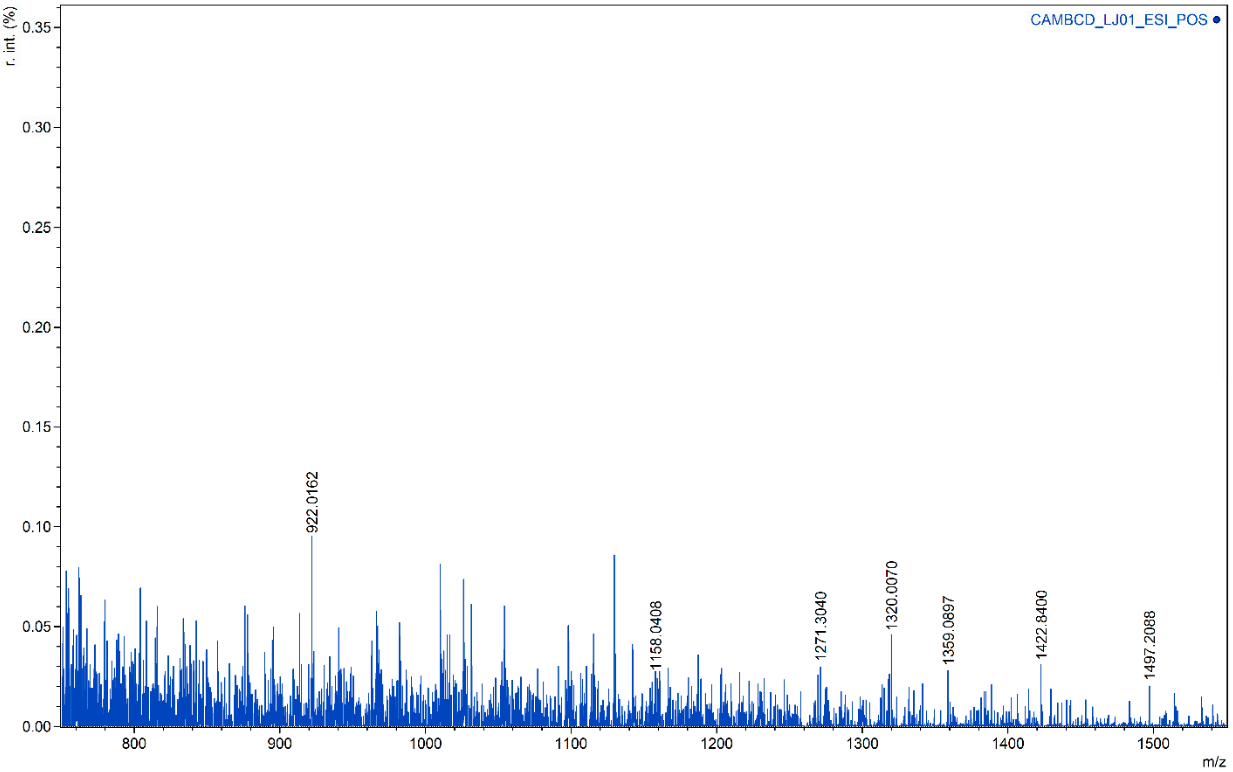

Meas. m/z	Calc. m/z	δ (Da)	Z	Annotation	Formula
634.6917	635.0289	-0.3372	2	+1 CH2CH2CONH2 +1 HaO	(C42H70O35Na2)1(CH1CH2CONH2)1(CH1C2CH2COOH)0(HCOOH)0(H2O)1
642.6066	643.7032	-1.0966	2	+1 CH2CH2COONH2	(C42H70O35Na2)1(CH1CH2CONH2)1(CH1C2CH2COOH)0(HCOOH)0(H2O)2
700.5969	700.2153	0.3816	2	+2 CH2CH2CONH2 +1 CH2CH2COONa	(C42H70O35Na2)1(CH1CH2CONH2)2(CH1CH2CONa)1
922.0162	923.7832	-1.7670	2	+5 CH2CH2CONH2 +4 CH2HC2COONa	(C42H70O35Na2)1(CH1CH2CONH2)5(CH1CH2CONa)4
1158.0408	1157.3590	0.6819	1	C42H70O35 (bCD)	C42H70O35Na
1271.3040	1271.3907	-0.0867	1	+1 CH2CH2COOH +1 H2O	(C42H70O35Na)1(CH1CH2CONH2)0(CH1C2CH2COOH)1(HCOOH)0(H2O)1

Figure S 31: Peak identification in ESI+ mass spectrum of carbamoylethylated ßCD (**5**) prepared in ball mill (item 9 in Table 1), DS ≈3.8-4.3

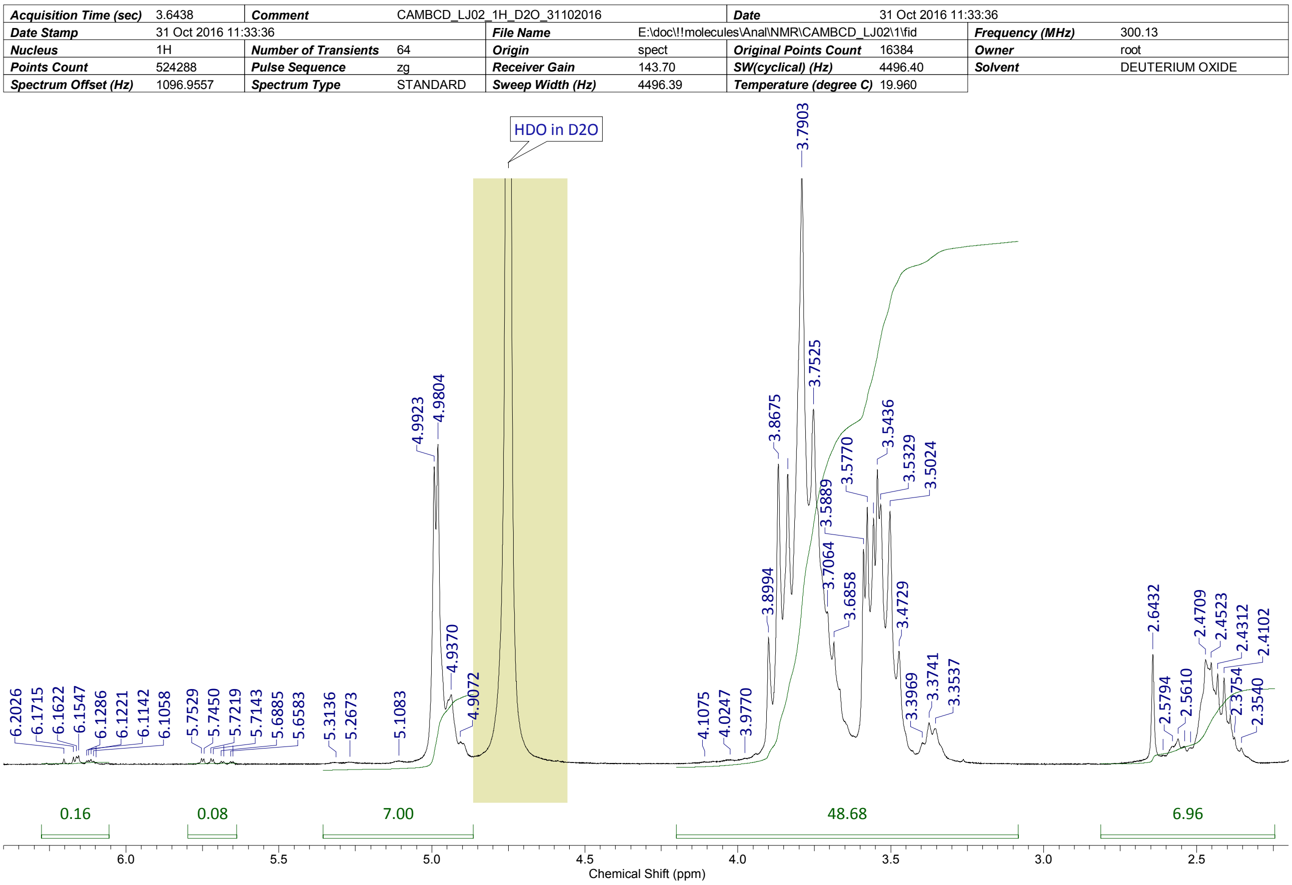

*Figure S 32: Proton NMR spectrum of carbamoylethylated βCD (**5**) prepared in ball mill (item 10 in Table 1), DS ≈3.3-3.5*

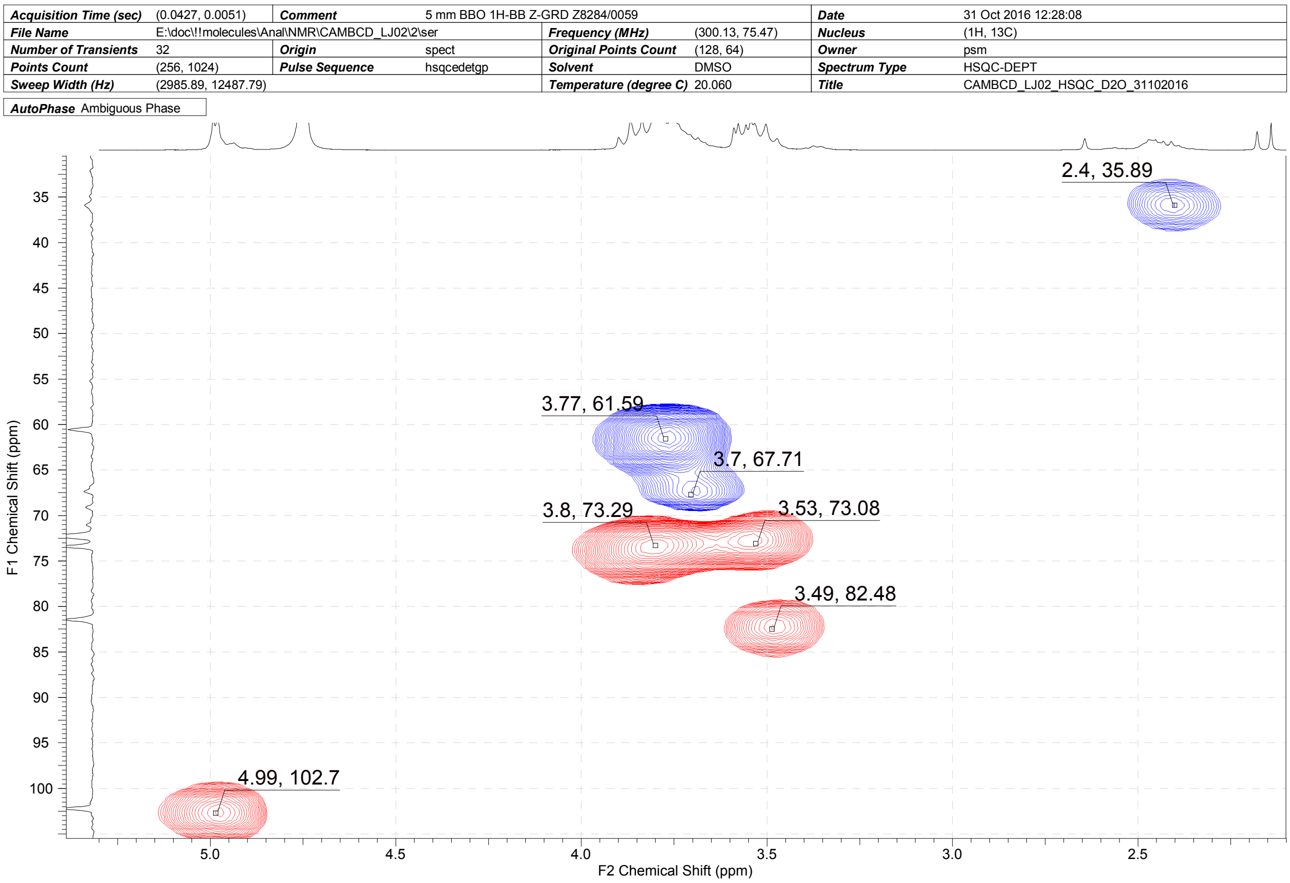

*Figure S 33: HSQC-DEPT spectrum of carbamoylethylated βCD (**5**) prepared in ball mill (item 10 in Table 1), DS ≈3.3-3.5*

Acquisition Time (sec)	1.8088	***Comment***	CAMBCD_LJ02_13C_D2O_03112016_2086_notte			***Date***	03 Nov 2016 13:45:52		
Date Stamp	03 Nov 2016 13:45:52			***File Name***	E:\doc\!!molecules\Anal\NMR\CAMBCD_LJ02\3\fid			***Frequency (MHz)***	75.47
Nucleus	13C	***Number of Transients***	1035	***Origin***	spect	***Original Points Count***	32768	***Owner***	root
Points Count	262144	***Pulse Sequence***	zgpg.save.txt	***Receiver Gain***	4597.60	***SW(cyclical) (Hz)***	18115.94	***Solvent***	DMSO-d6
Spectrum Offset (Hz)	8301.4463	***Spectrum Type***	STANDARD	***Sweep Width (Hz)***	18115.87	***Temperature (degree C)***	21.360		

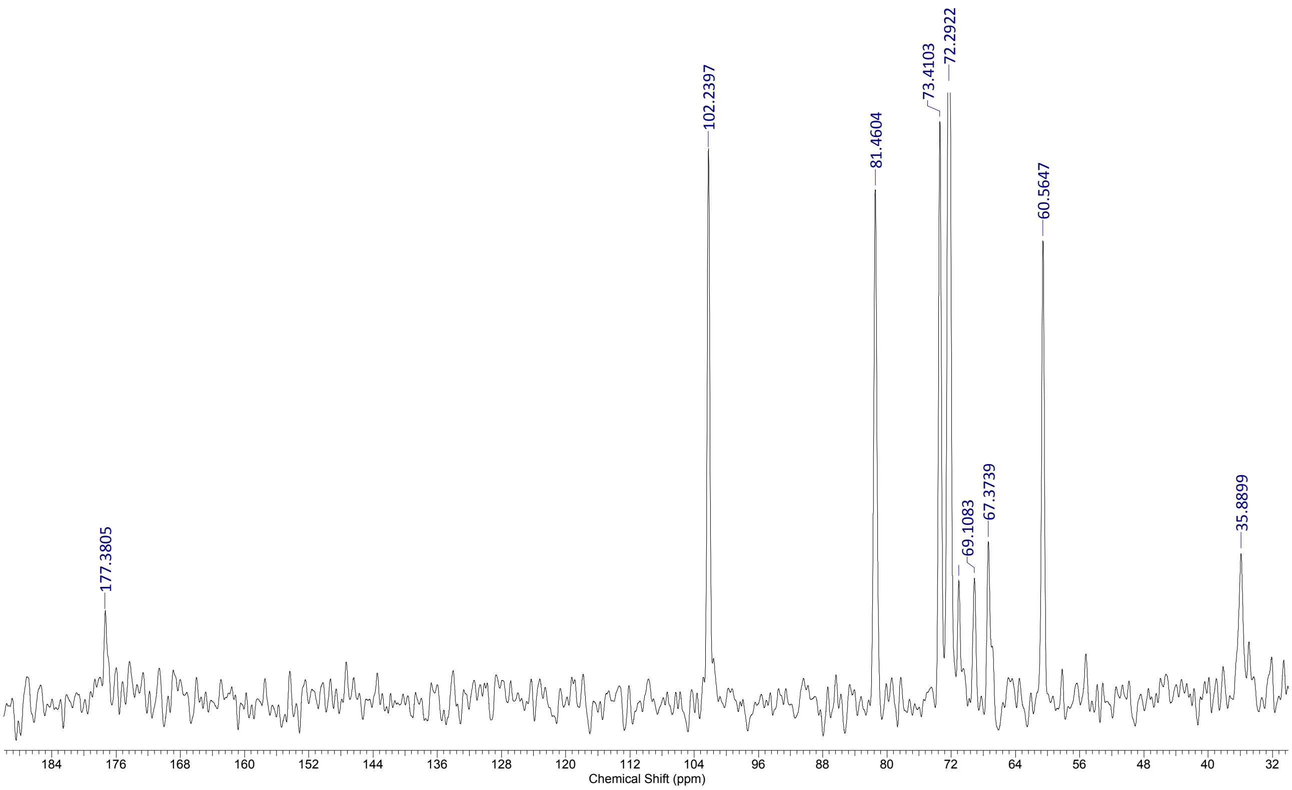

*Figure S 34: ^{13}C-NMR spectrum of carbamoylethylated ßCD (**5**) prepared in ball mill (item 10 in Table 1), DS ≈3.3-3.5*

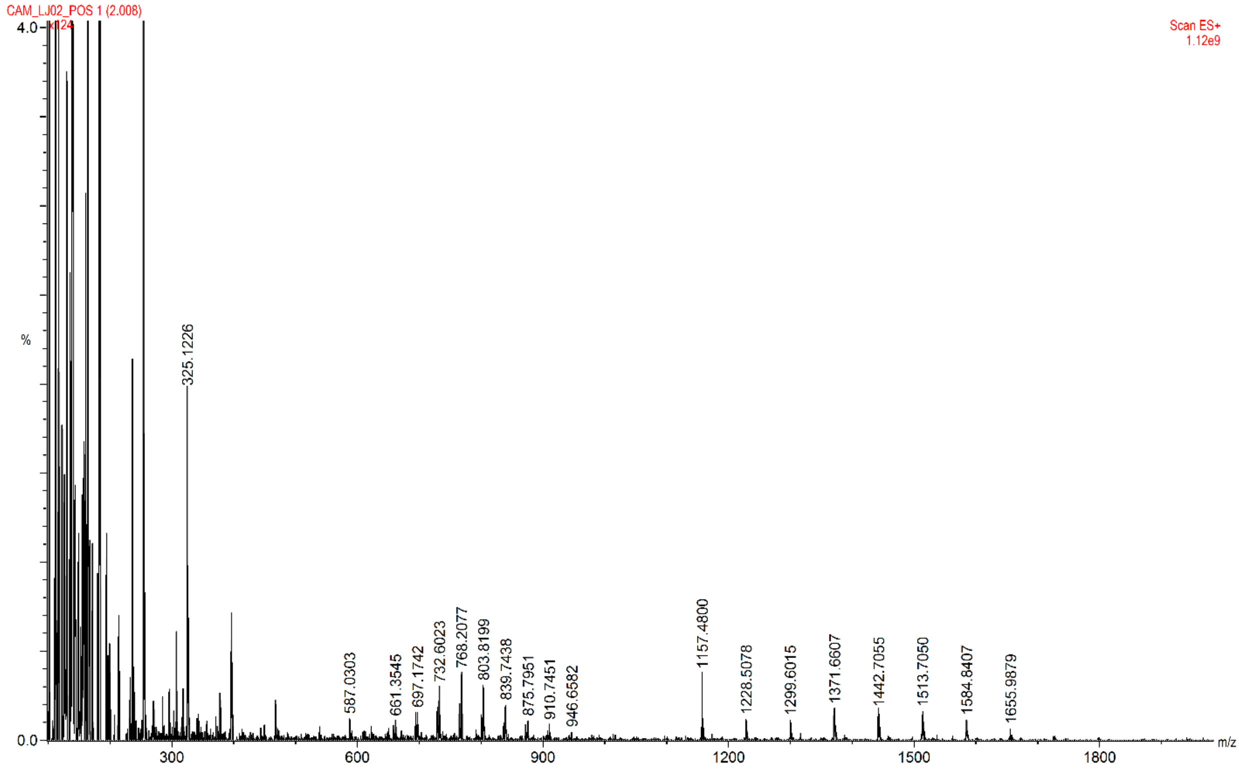

Figure S 35: ESI+ mass spectrum of carbamoylethylated ßCD (**5**) prepared in ball mill (item 10 in Table 1), DS ≈3.3-3.5

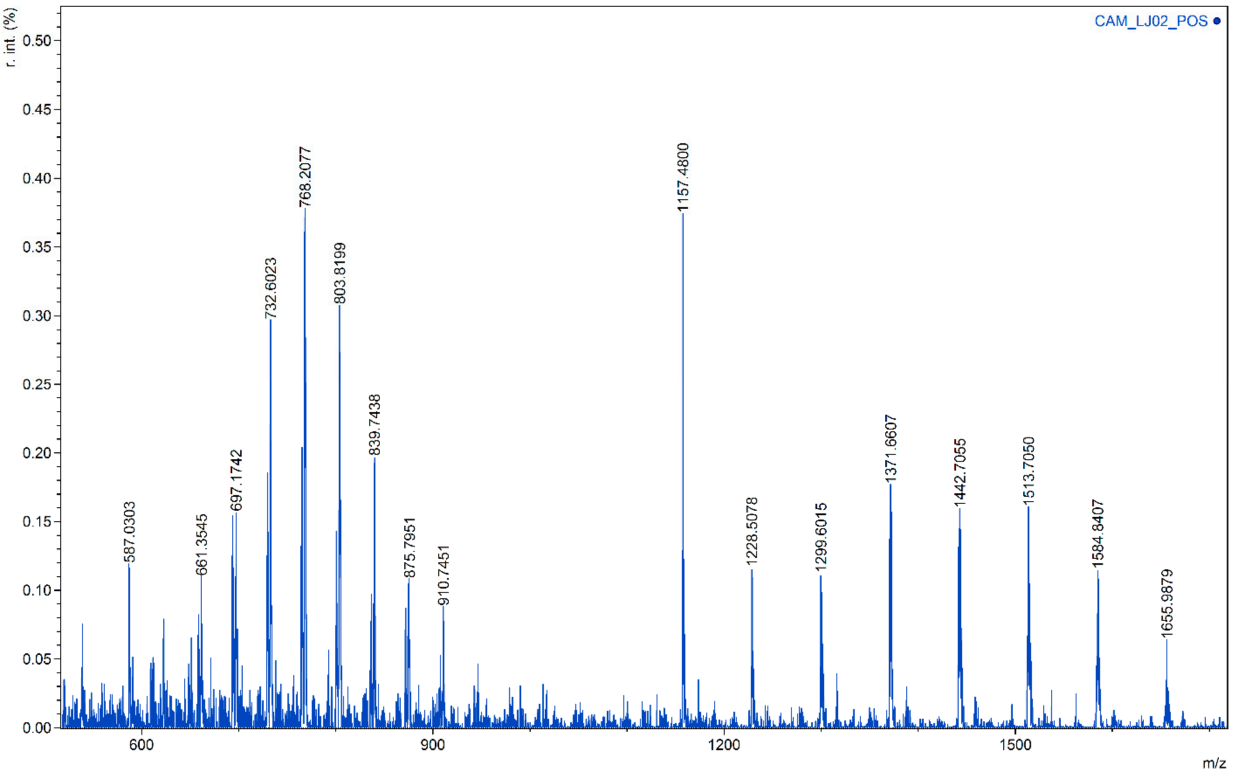

Meas. m/z	Calc. m/z	δ (Da)	Z	Annotation	Formula
661.3545	661.2112	0.1432	2	(+2 CH2CH2CONH2)/2	(C42H70O35Na2)1(CH1CH2CONH2)2
697.1742	696.7298	0.4444	2	(+3 CH2CH2CONH2)/2	(C42H70O35Na2)1(CH1CH2CONH2)3
732.6023	732.2483	0.3539	2	(+4 CH2CH2CONH2)/2	(C42H70O35Na2)1(CH1CH2CONH2)4
768.2077	767.7669	0.4409	2	(+5 CH2CH2CONH2)/2	(C42H70O35Na2)1(CH1CH2CONH2)5
803.8199	803.2854	0.5345	2	(+6 CH2CH2CONH2)/2	(C42H70O35Na2)1(CH1CH2CONH2)6
839.7438	838.8040	0.9398	2	(+7 CH2CH2CONH2)/2	(C42H70O35Na2)1(CH1CH2CONH2)7
875.7951	875.3185	0.4766	2	(+7 CH2CH2CONH2 +1 CH2CH2COOH(?))/2	(C42H70O35Na2)1(CH1CH2CONH2)7/8(CH2CH2COOH)1(?)
910.7451	909.8411	0.9040	2	(+8 CH2CH2CONH2 +1 CH2CH2COOH(?))/2	(C42H70O35Na2)1(CH1CH2CONH2)8/9(CH2CH2COOH)1(?)
1157.4800	1157.3590	0.1210	1	C42H70O35 (βCD)	(C42H70O35Na1)1(CH1CH2CONH2)0
1228.5078	1228.3961	0.1118	1	+1 CH2CH2CONH2	(C42H70O35Na)1(CH1CH2CONH2)1
1299.6015	1299.4332	0.1682	1	+2 CH2CH2CONH2	(C42H70O35Na)1(CH1CH2CONH2)2
1371.6607	1370.4703	1.1904	1	+3 CH2CH2CONH2	(C42H70O35Na)1(CH1CH2CONH2)3
1442.7055	1441.5074	1.1981	1	+4 CH2CH2CONH2	(C42H70O35Na1)1(CH1CH2CONH2)4
1513.7050	1512.5446	1.1604	1	+5 CH2CH2CONH2	(C42H70O35Na1)1(CH1CH2CONH2)5
1584.8407	1583.5817	1.2590	1	+6 CH2CH2CONH2	(C42H70O35Na1)1(CH1CH2CONH2)6
1655.9879	1654.6188	1.3691	1	+7 CH2CH2CONH2	(C42H70O35Na1)1(CH1CH2CONH2)7

Figure S 36: Peak identification in ESI+ mass spectrum of carbamoylethylated ßCD (**5**) prepared in ball mill (item 10 in Table 1), DS ≈3.8-4.3

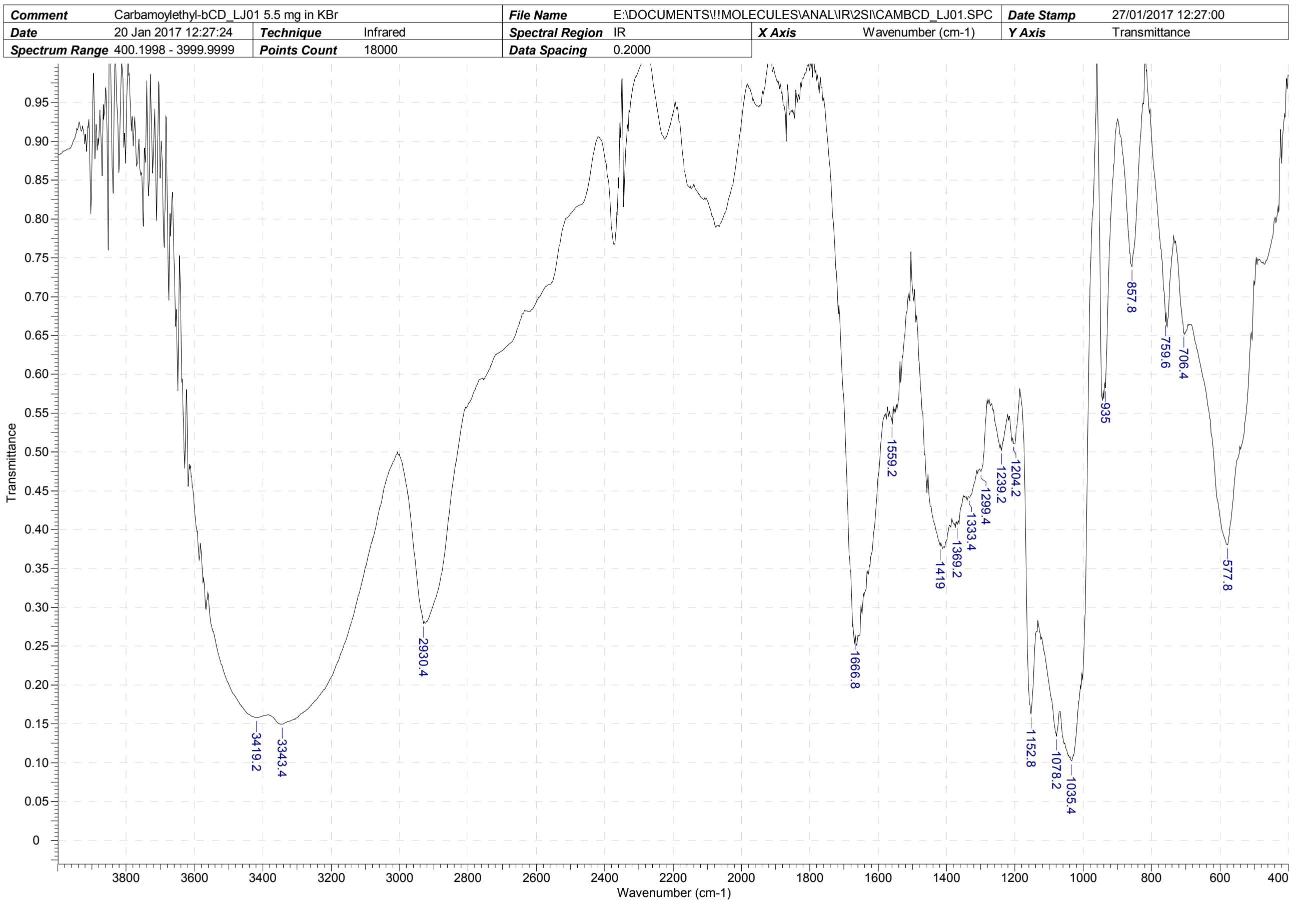

*Figure S 37: IR spectrum of carbamoylethylated βCD (**5**) prepared in ball mill (item 10 in Table 1), DS ≈3.3-3.5*

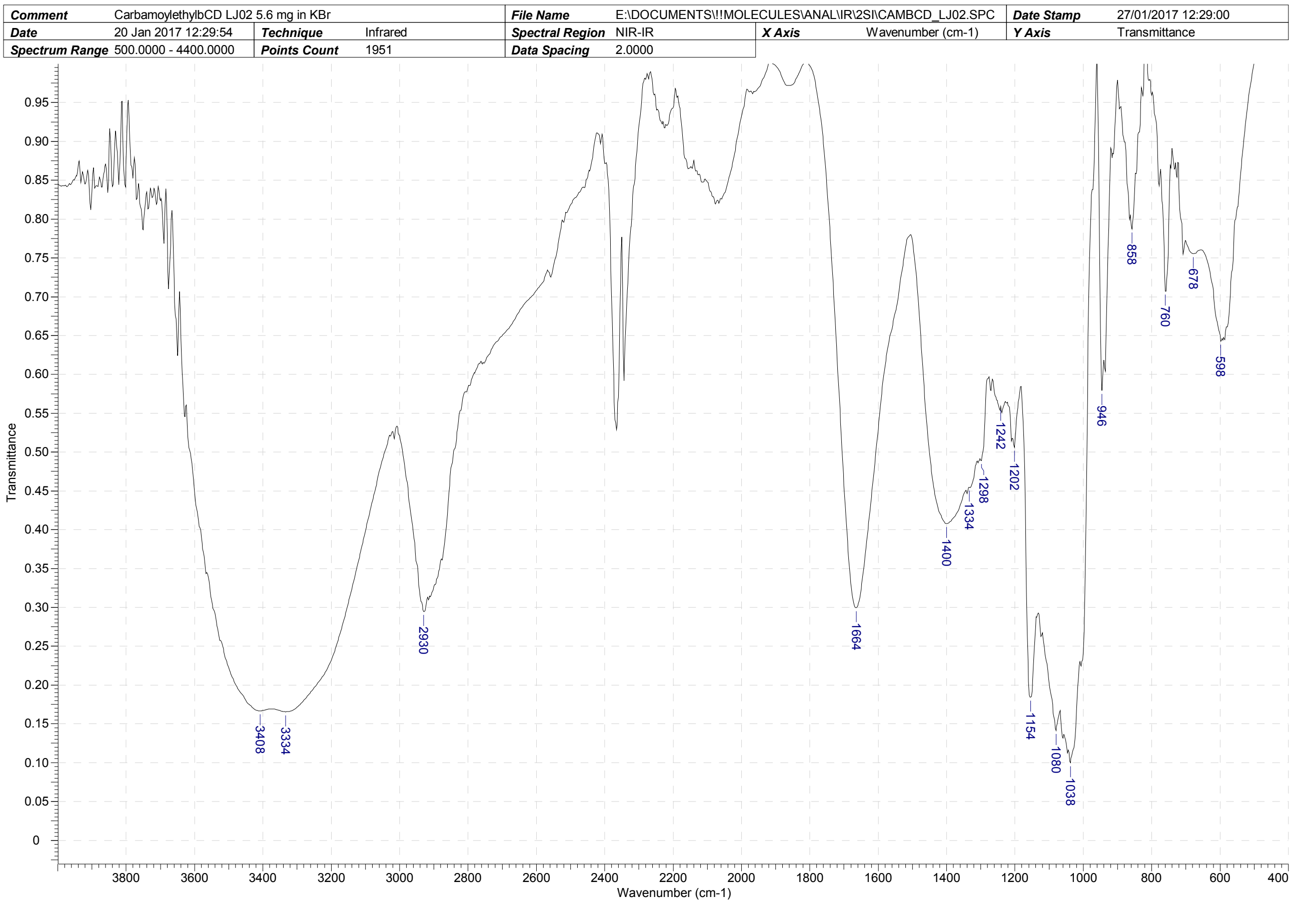

*Figure S 38: IR spectrum of carbamoylethylated βCD (**5**) prepared in ball mill (item 10 in Table 1), DS ≈3.3-3.5*

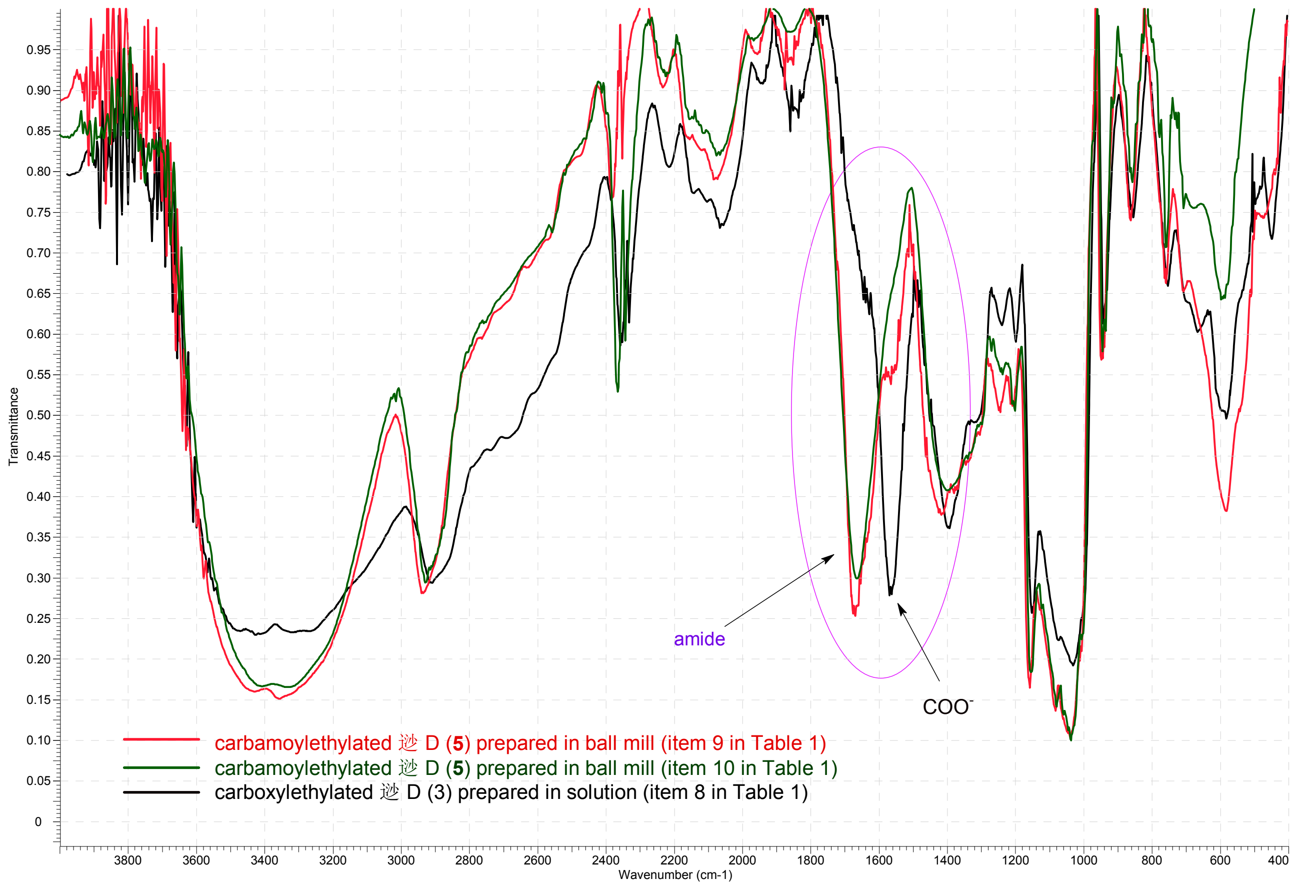

*Figure S 39: Overlay of IR spectra of carbamoylethylated βCD (**5**) prepared in ball mill (item 9&10 in Table 1), DS ≈3.3-3.5 carboxyethylated βCD prepared in solution (item 8 in Table 1)*

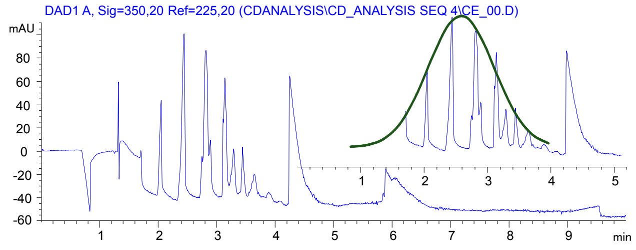

Carboxyethylated βCD (**3**) DS 3.0-3.4, synthesized in solution(item 8 in Table 1)

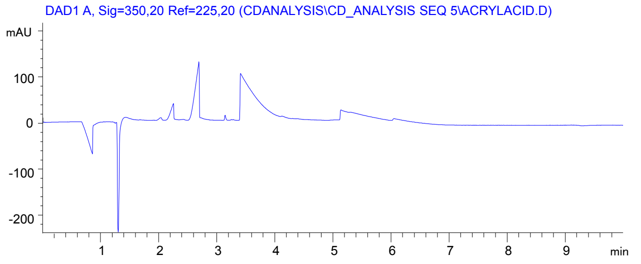

Acrylic acid sodium salt

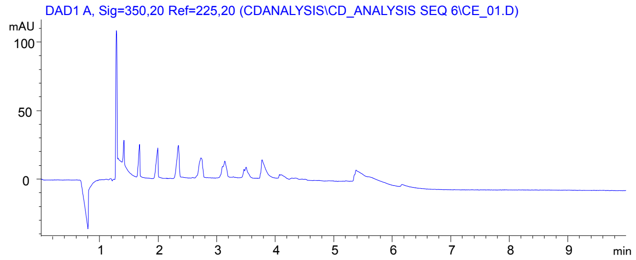

Carboxyethylated βCD (**3’**) DS 3.0-3.3, synthesis in ball mill (item 5 in Table 1)

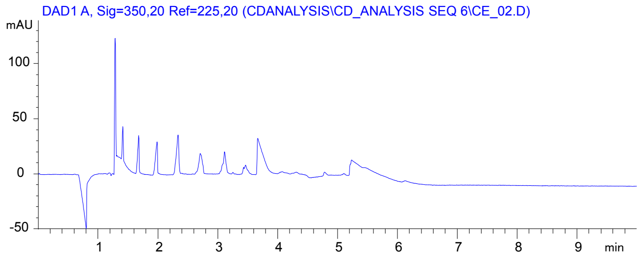

Carboxyethylated βCD (**3’**) DS 2.8-3.2, synthesis in ball mill (item 6 in Table 1)

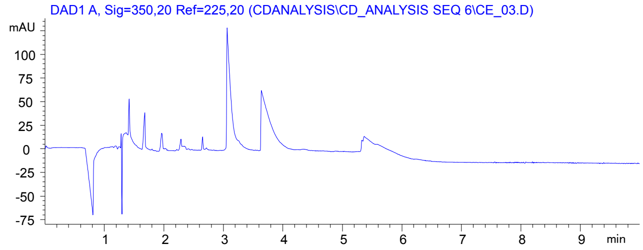

Carboxyethylated βCD (**3’’**) DS 2.8-3.1, synthesis in ball mill (item 7 in Table 1)

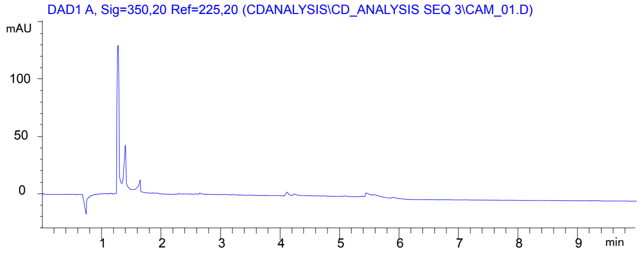

Carboxyethyl residue in carbamoylethylated βCD (**5**) DS 3.8-4.3 (item 9 in Table 1)

Figure S 40: Capillary electropherogram of carboxyethylated βCDs

Acquisition Time (sec)	4.0000	***Comment***	samplename: SBbCD_00SOL solvent: d2o temp: 25C probe: sw8099				
Date	Dec 17 2010	***Date Stamp***	Dec 17 2010	***File Name***	E:\doc\!!molecules\Anal\NMR\SBbCD_00SOL\s2pul_d2o\fid		
Frequency (MHz)	399.91	***Nucleus***	1H	***Number of Transients***	16	***Original Points Count***	40000
Points Count	65536	***Pulse Sequence***	s2pul	***Receiver Gain***	20.00	***Solvent***	DEUTERIUM OXIDE
Spectrum Offset (Hz)	1992.8751	***Spectrum Type***	STANDARD	***Sweep Width (Hz)***	10000.00	***Temperature (degree C)***	25.000

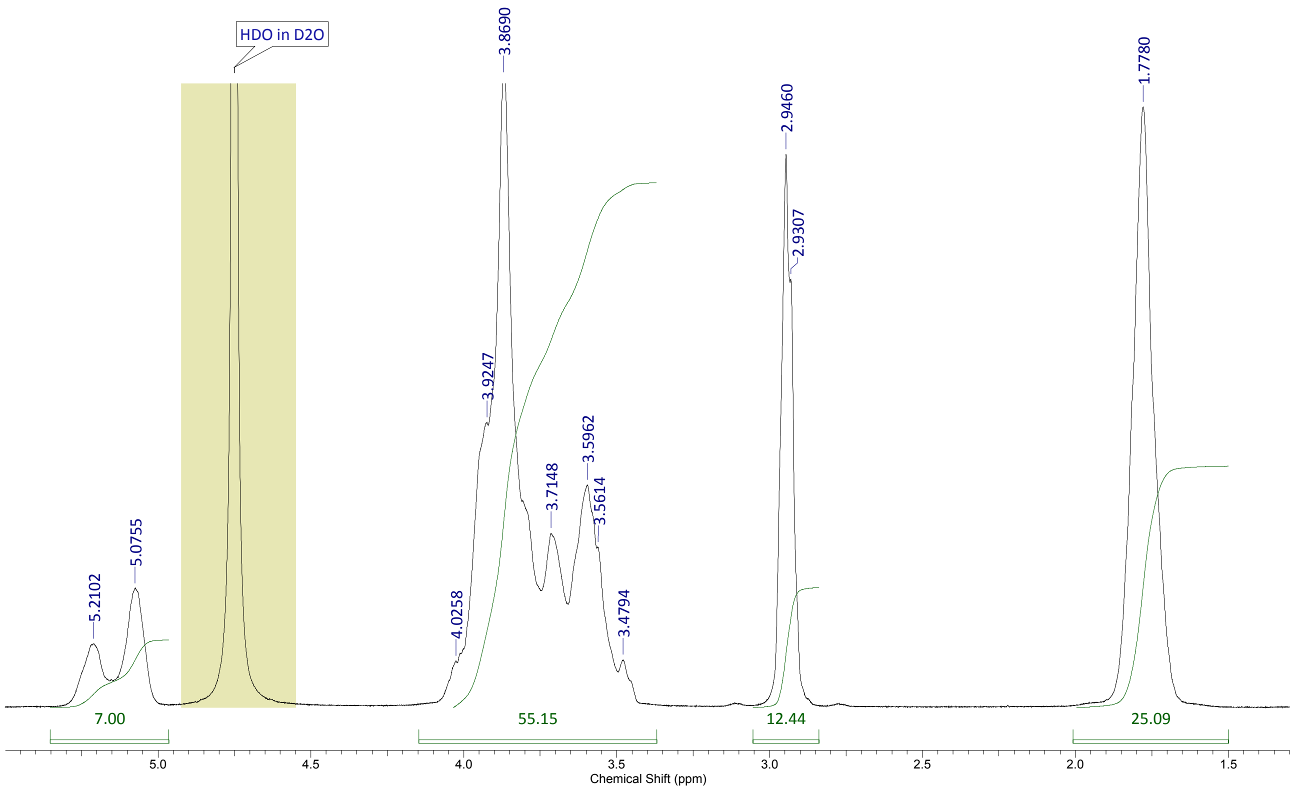

*Figure S 41: Proton NMR spectrum of sulfobutylated βCD (**4**) prepared in solution (item 11 in Table 1), DS ≈6.2-6.5*

Acquisition Time (sec)	(0.1500, 0.0023)	***Comment***	samplename: SBbCD_00SOL solvent: d2o temp: 25C probe: sw8099			***Date***	17 Dec 2010 09:21:12
Date Stamp	Dec 17 2010	***File Name***	E:\doc\!!molecules\Anal\NMR\SBbCD_00SOL\ghsqc_d2o\fid			***Frequency (MHz)***	(399.91, 100.57)
Nucleus	(1H, 13C)	***Number of Transients***	16	***Original Points Count***	(1500, 64)	***Points Count***	(8192, 256)
Pulse Sequence	gHSQC	***Solvent***	d2o	***Spectrum Type***	HSQC	***Sweep Width (Hz)***	(9998.78, 28298.12)

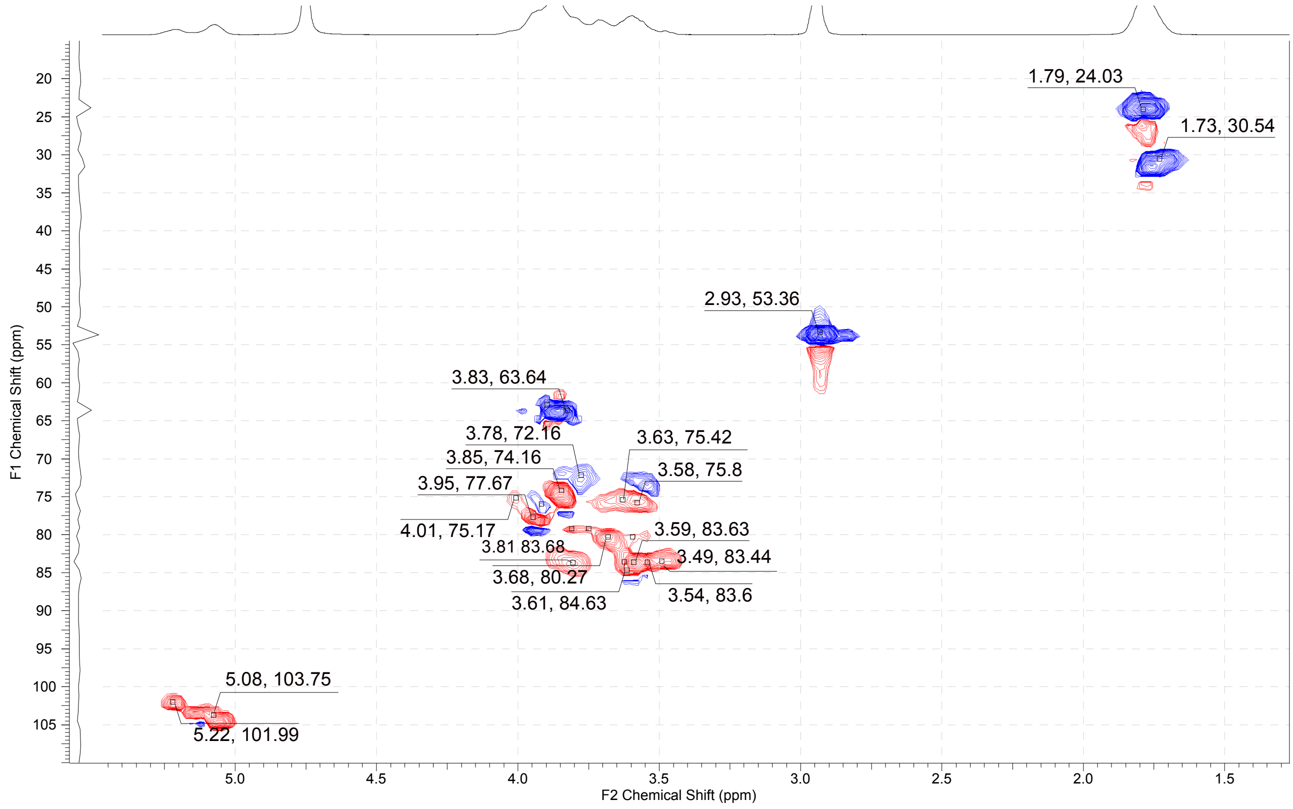

*Figure S 42: HSQC-DEPT spectrum of sulfobutylated βCD (**4**) prepared in solution (item 11 in Table 1), DS ≈6.2-6.5*

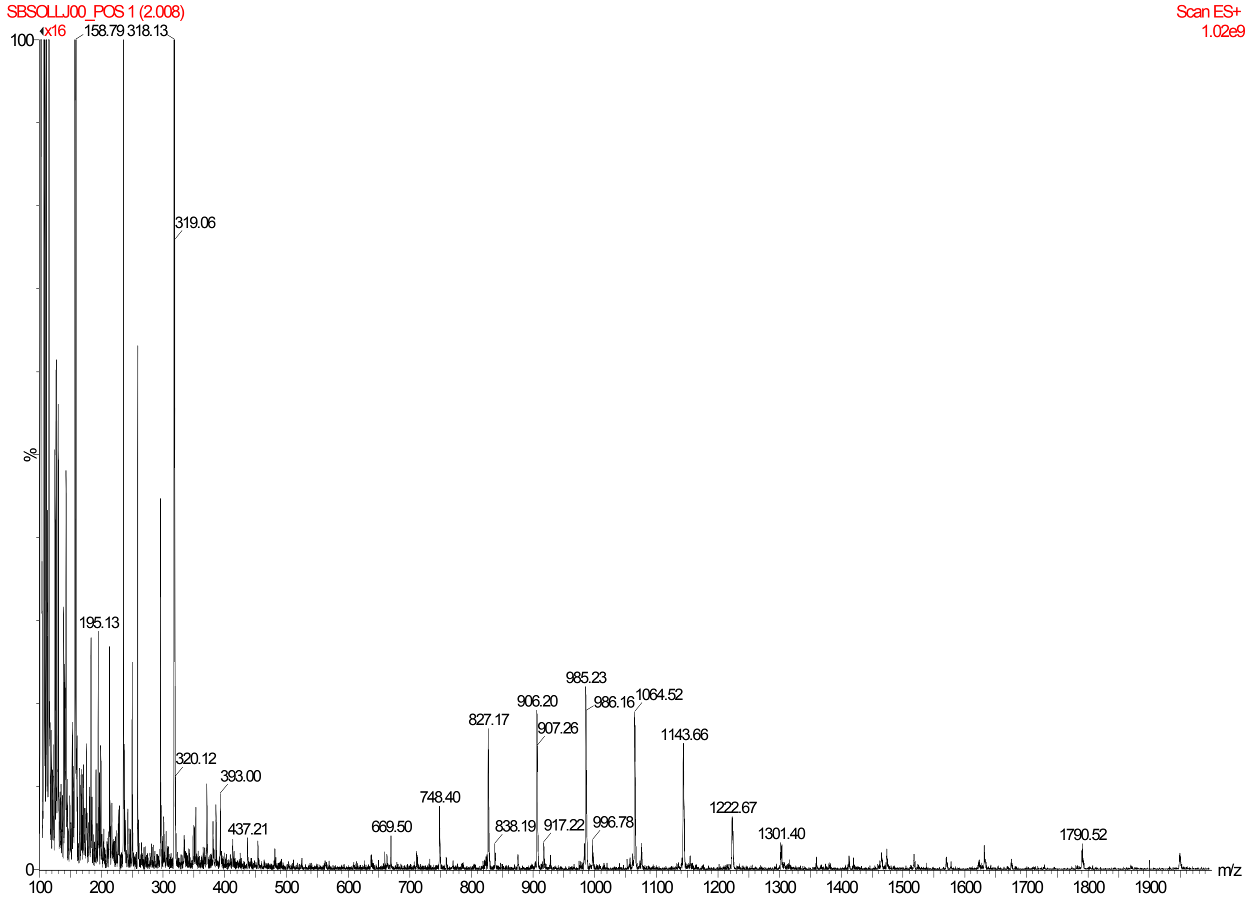

*Figure S 43: ESI+ mass spectrum of sulfobutylated βCD (**4**) prepared in solution (item 11 in Table 1), DS ≈6.2-6.5*

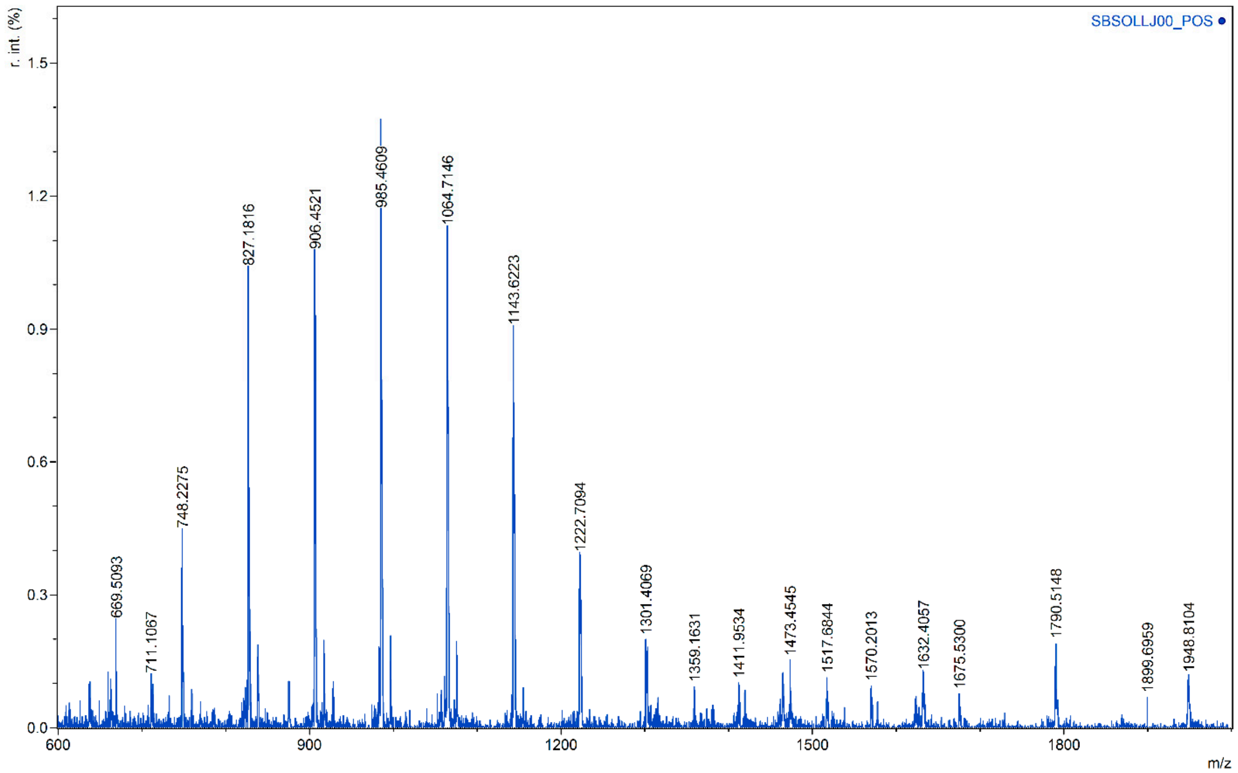

Meas. m/z	Calc. m/z	δ (Da)	Z	Annotation	Formula
669.5093	669.1748	0.3345	2	(+1 C4H8SO3Na)/2	(C42H70O35Na2)1(C4H7SO3H)0(C4H7SO3Na)1
711.1067	709.7879	1.3188	3	(+1 C4H8SO3H +5 C4H8SO3Na)/3	(C42H70O35Na3)1(C4H7SO3H)1(C4H7SO3Na)5
748.2275	748.1755	0.0520	2	(+2 C4H8SO3Na)/2	(C42H70O35Na2)1(C4H7SO3H)0(C4H7SO3Na)2
827.1816	827.1761	0.0054	2	(+3 C4H8SO3Na)/2	(C42H70O35Na2)1(C4H7SO3H)0(C4H7SO3Na)3
906.4521	906.1768	0.2752	2	(+4 C4H8SO3Na)/2	(C42H70O35Na2)1(C4H7SO3H)0(C4H7SO3Na)4
985.4609	985.1775	0.2834	2	(+5 C4H8SO3Na)/2	(C42H70O35Na2)1(C4H7SO3H)0(C4H7SO3Na)5
1064.7146	1064.1782	0.5364	2	(+6 C4H8SO3Na)/2	(C42H70O35Na2)1(C4H7SO3H)0(C4H7SO3Na)6
1143.6223	1143.1789	0.4434	2	(+7 C4H8SO3Na)/2	(C42H70O35Na2)1(C4H7SO3H)0(C4H7SO3Na)7
1222.7094	1222.1795	0.5299	2	(+8 C4H8SO3Na)/2	(C42H70O35Na2)1(C4H7SO3H)0(C4H7SO3Na)8
1301.4069	1301.1802	0.2267	2	(+9 C42H70O35Na2)/2	(C42H70O35Na2)1(C4H7SO3H)0(C4H7SO3Na)9
1315.4595	1315.3603	0.0991	1	+1 C4H8SO3Na	(C42H70O35Na1)1(C4H7SO3H)0(C4H7SO3Na)1
1359.1631	1358.1990	0.9642	2	(+2 C4H8SO3H)/2	(C42H70O35Na2)1(C4H7SO3H)2(C4H7SO3Na)8
1473.4545	1473.3617	0.0928	1	+2 C4H8SO3Na	(C42H70O35Na1)1(C4H7SO3H)0(C4H7SO3Na)2
1517.6844	1516.2003	1.4841	2	(+2 C4H8SO3H +10 C4H8SO3Na)/2	(C42H70O35Na2)1(C4H7SO3H)2(C4H7SO3Na)10
1632.4057	1631.3631	1.0427	1	+3 C4H8SO3Na	(C42H70O35Na1)1(C4H7SO3H)0(C4H7SO3Na)3
1675.5300	1674.2017	1.3283	2	(+2 C4H8SO3H +12 C4H8SO3Na)/2	(C42H70O35Na2)1(C4H7SO3H)2(C4H7SO3Na)12
1790.5148	1789.3644	1.1503	1	+4 C4H8SO3Na	(C42H70O35Na1)1(C4H7SO3H)0(C4H7SO3Na)4
1948.8104	1947.3658	1.4446	1	+5 C4H8SO3Na	(C42H70O35Na1)1(C4H7SO3H)0(C4H7SO3Na)5

*Figure S 44: Peak identification in ESI+ mass spectrum of sulfobutylated βCD (**4**) prepared in solution (item 11 in Table 1), DS ≈6.2-6.5*

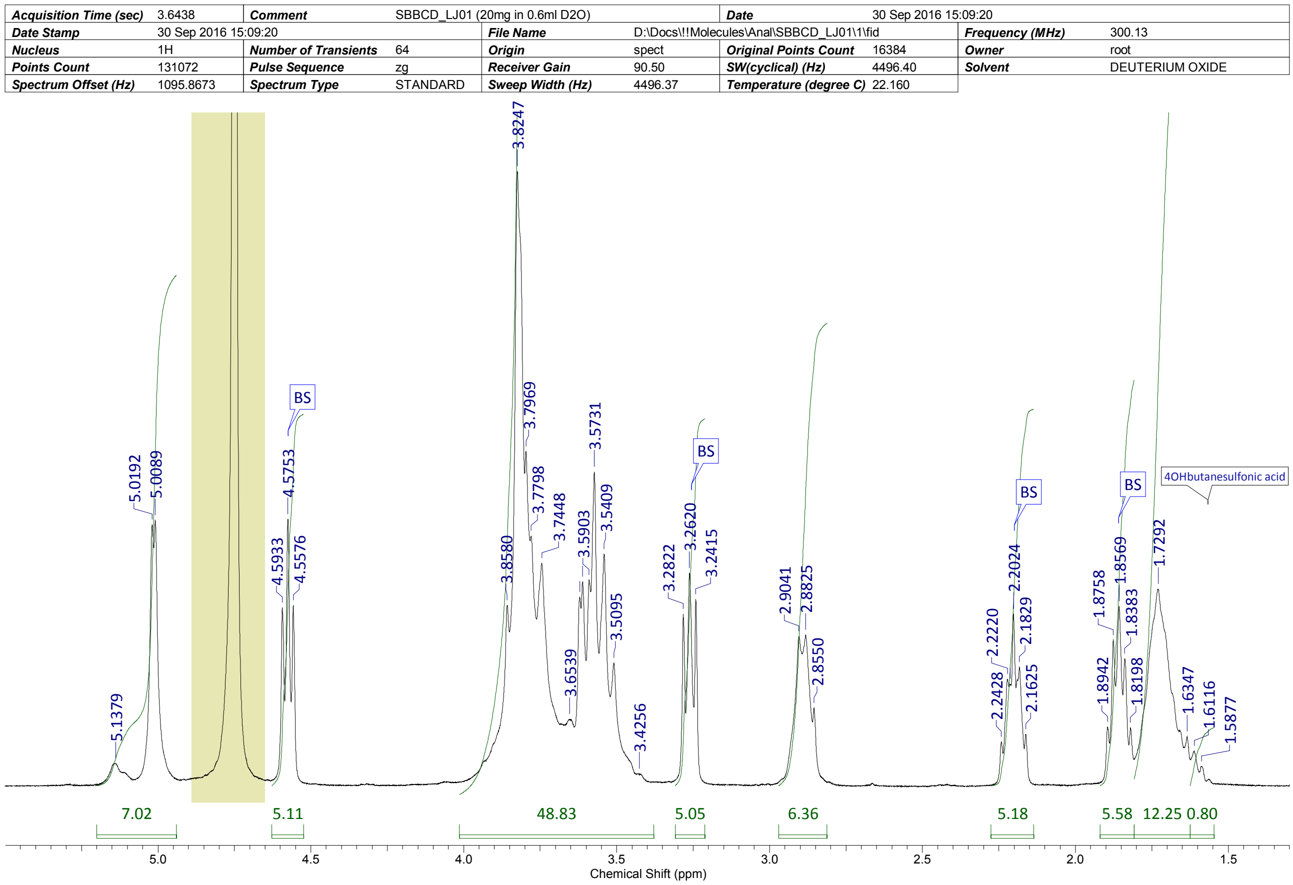

*Figure S 45: Proton NMR spectrum of sulfobutylated βCD (**4'**) prepared in ball mill (item 12 in Table 1), DS ≈3.1-3.3*

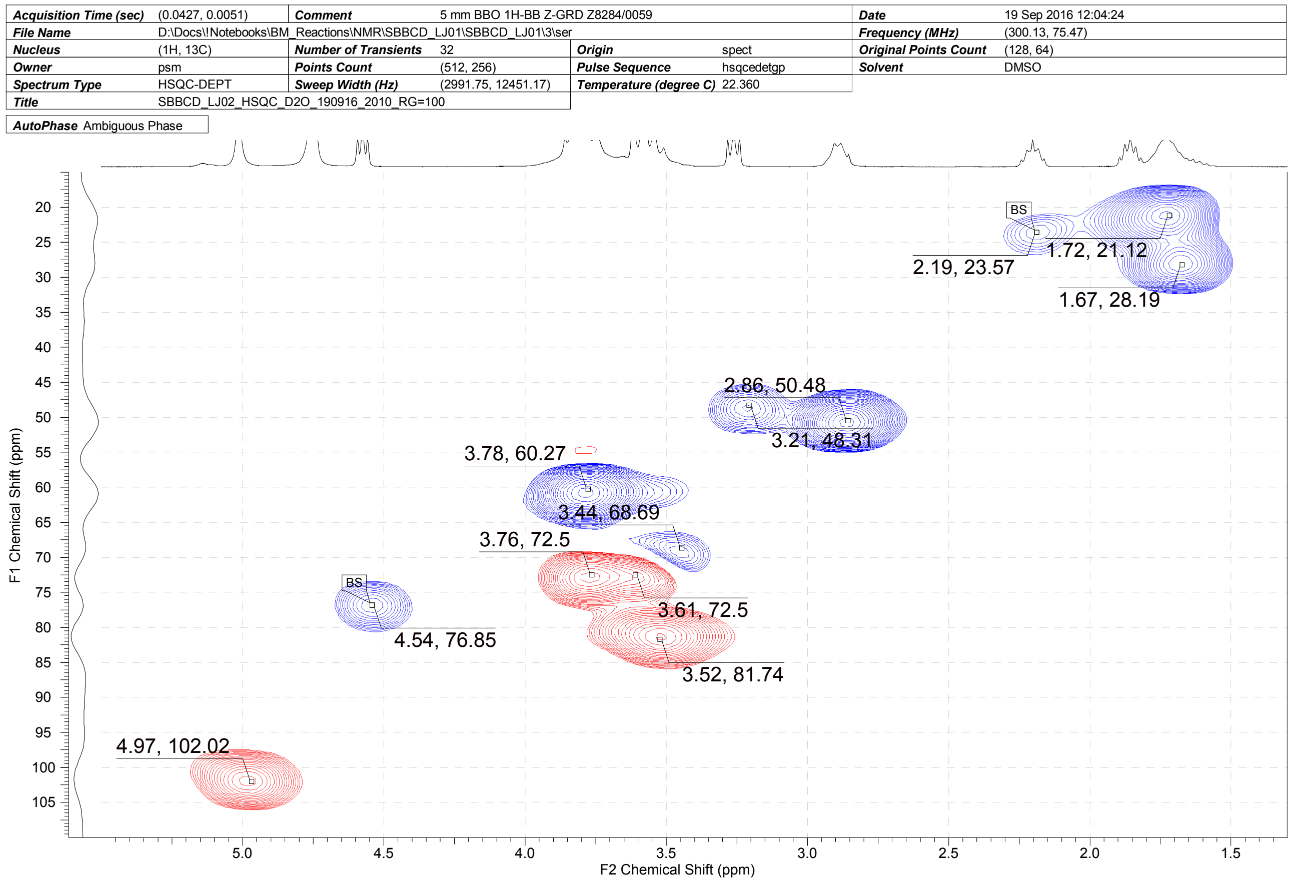

*Figure S 46: HSQC-DEPT spectrum of sulfobutylated βCD (**4'**) prepared in ball mill (item 12 in Table 1), DS ≈3.1-3.3*

Acquisition Time (sec)	3.6438	***Comment***	SBBCD_LJ03 (20mg in 0.6ml D2O)			***Date***	28 Sep 2016 12:54:56		
Date Stamp	28 Sep 2016 12:54:56			***File Name***	D:\Docs\!!Notebooks\BM_Reactions\NMR\SBBCD_LJ03\1\fid				
Frequency (MHz)	300.13	***Nucleus***	1H	***Number of Transients***	64	***Origin***	spect	***Original Points Count***	16384
Owner	root	***Points Count***	131072	***Pulse Sequence***	zg	***Receiver Gain***	90.50	***SW(cyclical) (Hz)***	4496.40
Solvent	DMSO-d6	***Spectrum Offset (Hz)***	1096.1024	***Spectrum Type***	STANDARD	***Sweep Width (Hz)***	4496.37	***Temperature (degree C)***	22.160

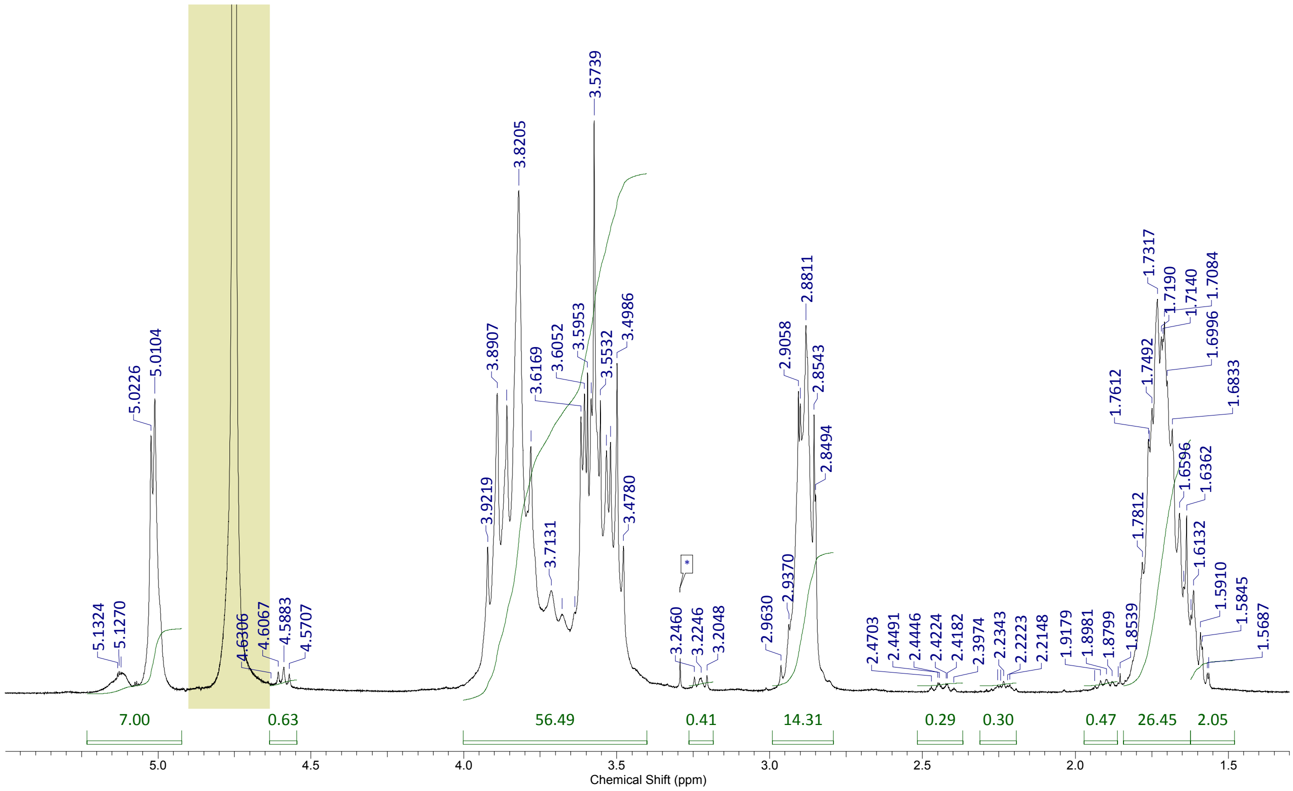

*Figure S 47: Proton NMR spectrum of sulfobutylated βCD (**4’**) prepared in ball mill (item 13 in Table 1), DS ≈5.2-5.8*

Acquisition Time (sec)	(0.0427, 0.0051)	***Comment***	5 mm BBO 1H-BB Z-GRD Z8284/0059			***Date***	28 Sep 2016 13:48:32
File Name	D:\Docs\!Notebooks\BM_Reactions\NMR\SBBCD_LJ03\2\ser			***Frequency (MHz)***	(300.13, 75.47)	***Nucleus***	(1H, 13C)
Number of Transients	32	***Origin***	spect	***Original Points Count***	(128, 64)	***Owner***	psm
Points Count	(512, 256)	***Pulse Sequence***	hsqcedetgp	***Solvent***	DMSO	***Spectrum Type***	HSQC-DEPT
Sweep Width (Hz)	(2991.75, 12451.17)			***Temperature (degree C)***	22.260		

AutoPhase	Ambiguous Phase

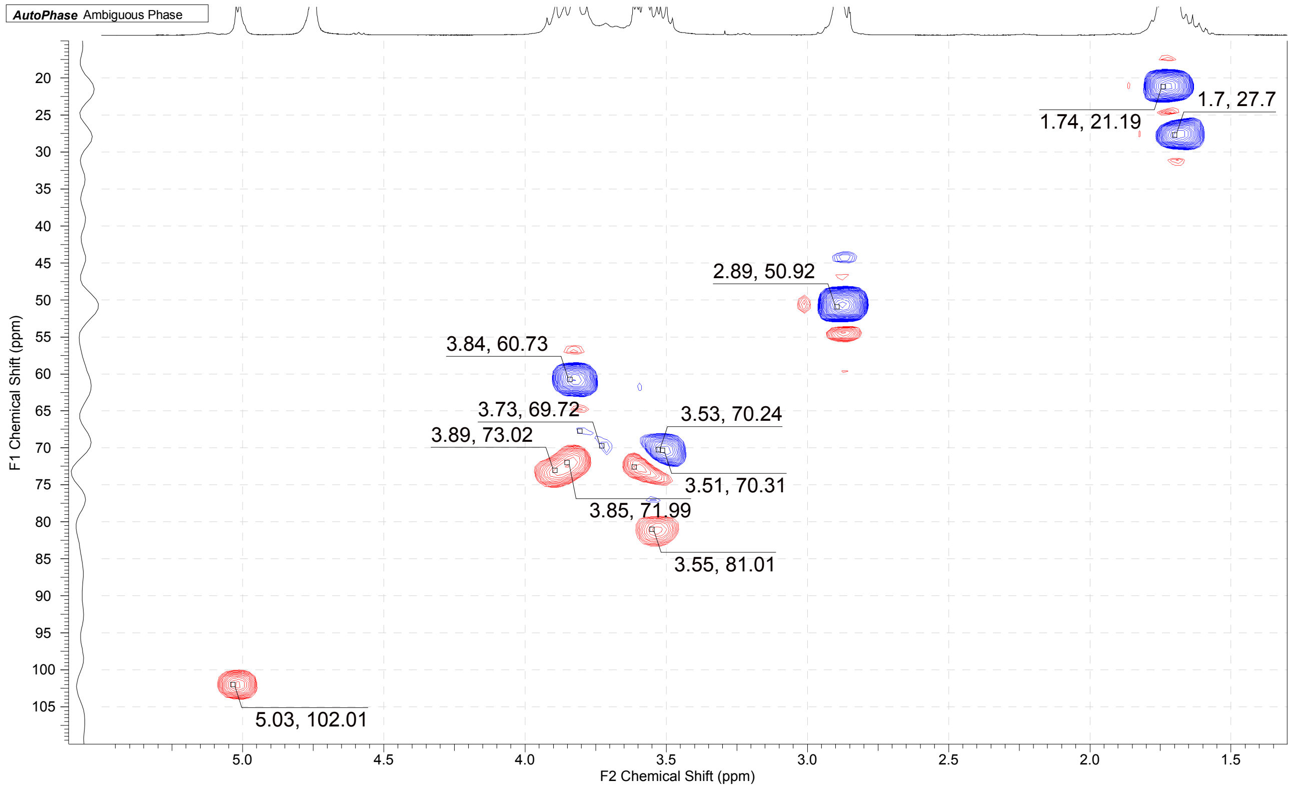

*Figure S 48: HSQC-DEPT spectrum of sulfobutylated βCD (**4’**) prepared in ball mill (item 13 in Table 1), DS ≈5.2-5.8*

**Acquisition Time (sec)**	3.6438	**Comment**	SBBCD_LJ03 (20mg in 0.6ml D2O)			**Date**	28 Sep 2016 12:54:56		
**Date Stamp**	28 Sep 2016 12:54:56			**File Name**	D:\Docs\!!Notebooks\BM_Reactions\NMR\SBBCD_LJ03\1\fid				
**Frequency (MHz)**	300.13	**Nucleus**	1H	**Number of Transients**	64	**Origin**	spect	**Original Points Count**	16384
**Owner**	root	**Points Count**	131072	**Pulse Sequence**	zg	**Receiver Gain**	90.50	**SW(cyclical) (Hz)**	4496.40
**Solvent**	DMSO-d6	**Spectrum Offset (Hz)**	1096.1024	**Spectrum Type**	STANDARD	**Sweep Width (Hz)**	4496.37	**Temperature (degree C)**	22.160

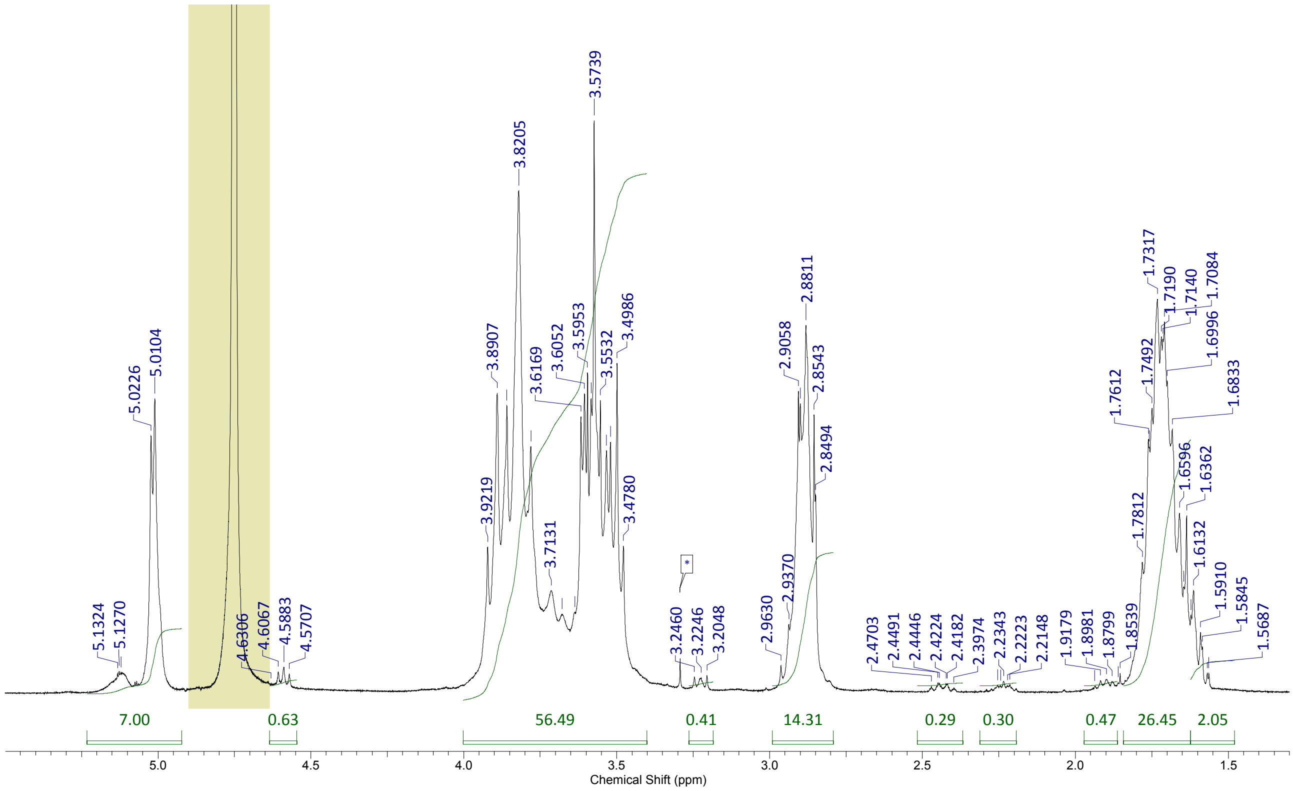

*Figure S 49: Proton NMR spectrum of sulfobutylated ßCD (**4'**) prepared in ball mill (item 14 in Table 1), DS ≈6.2-6.6*

**Acquisition Time (sec)**	(0.0427, 0.0051)	**Comment**	5 mm BBO 1H-BB Z-GRD Z8284/0059			**Date**	28 Sep 2016 13:48:32
**File Name**	D:\Docs\!!Notebooks\BM_Reactions\NMR\SBBCD_LJ03\2\ser			**Frequency (MHz)**	(300.13, 75.47)	**Nucleus**	(1H, 13C)
**Number of Transients**	32	**Origin**	spect	**Original Points Count**	(128, 64)	**Owner**	psm
**Points Count**	(512, 256)	**Pulse Sequence**	hsqcedetgp	**Solvent**	DMSO	**Spectrum Type**	HSQC-DEPT
**Sweep Width (Hz)**	(2991.75, 12451.17)			**Temperature (degree C)**	22.260		

**AutoPhase**	Ambiguous Phase

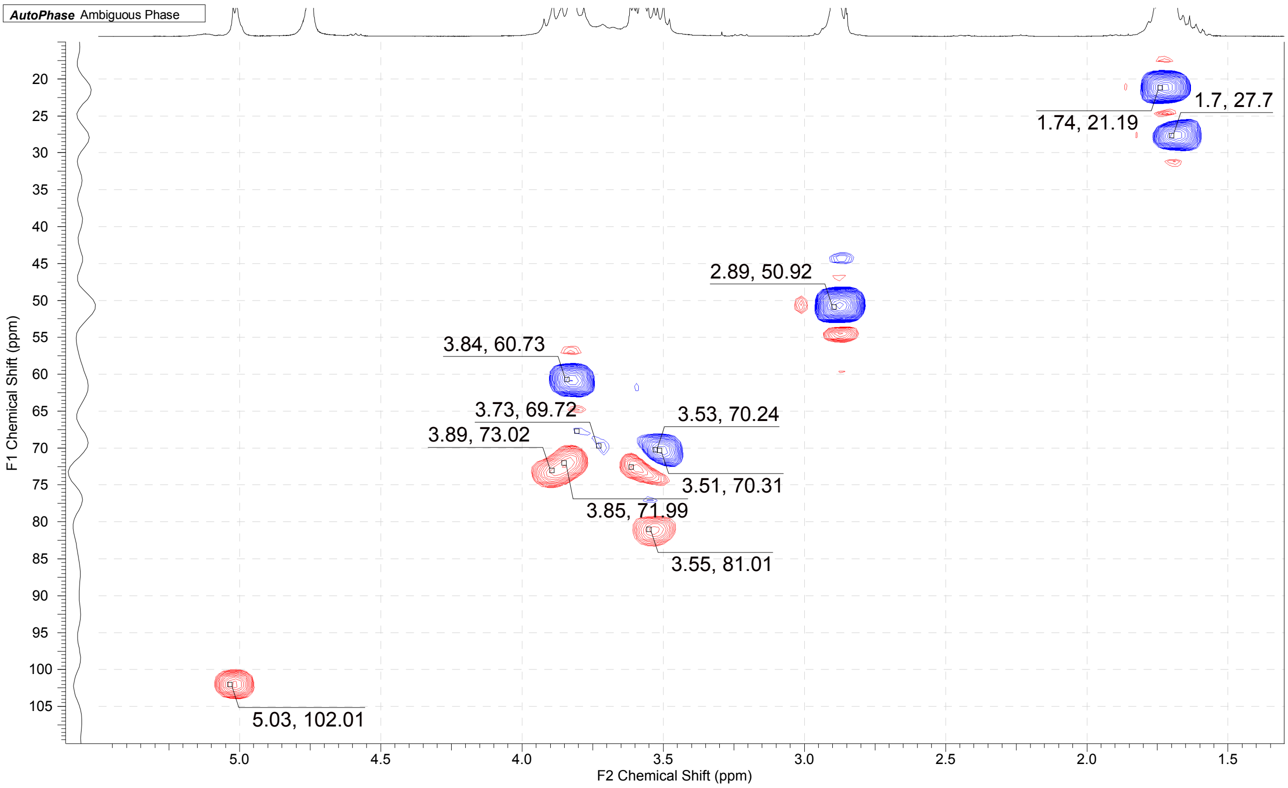

*Figure S 50: HSQC-DEPT spectrum of sulfobutylated ßCD (**4'**) prepared in ball mill (item 14 in Table 1), DS ≈6.2-6.6*

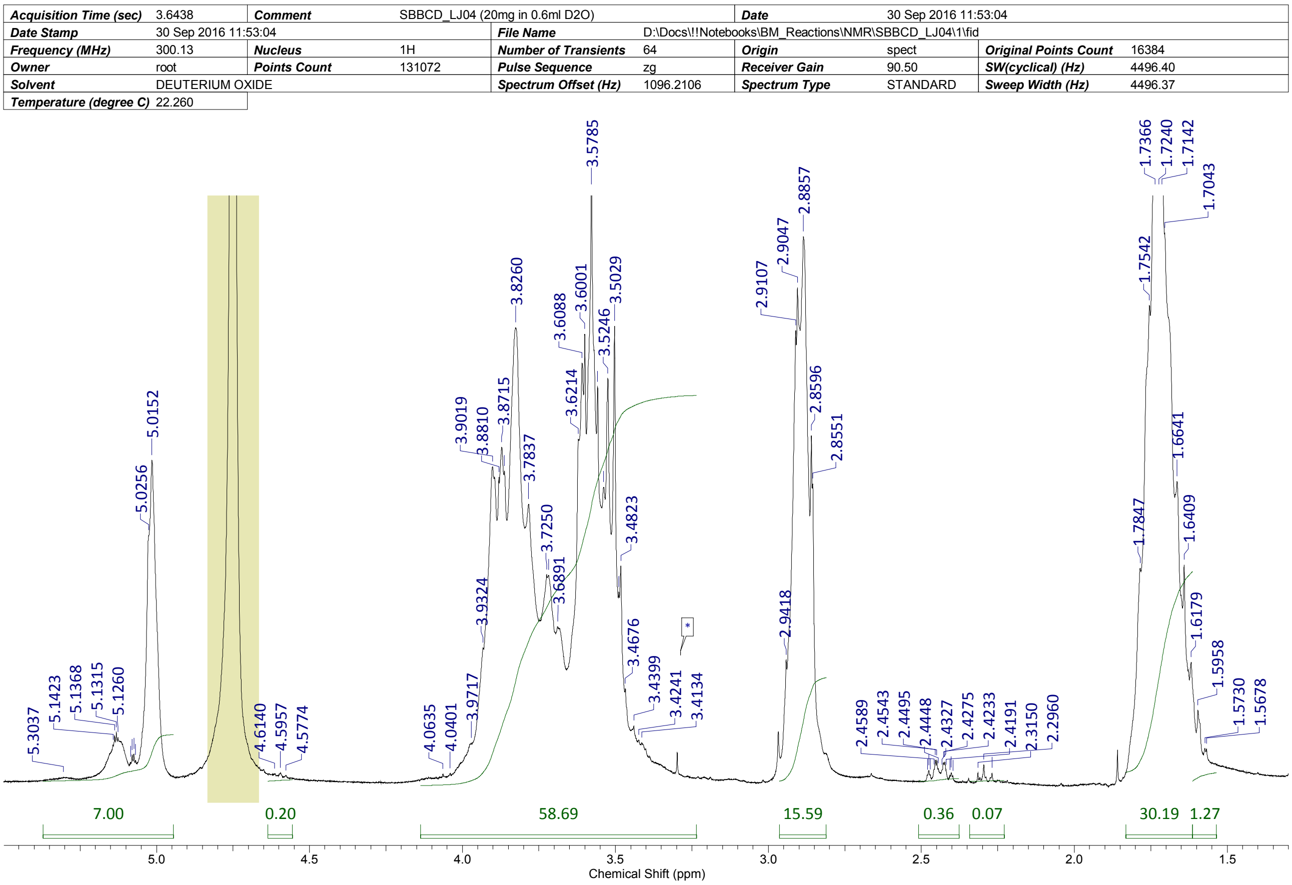

*Figure S 51: Proton NMR spectrum of sulfobutylated βCD (**4'**) prepared in ball mill (item 15 in Table 1), DS ≈7.5-7.8*

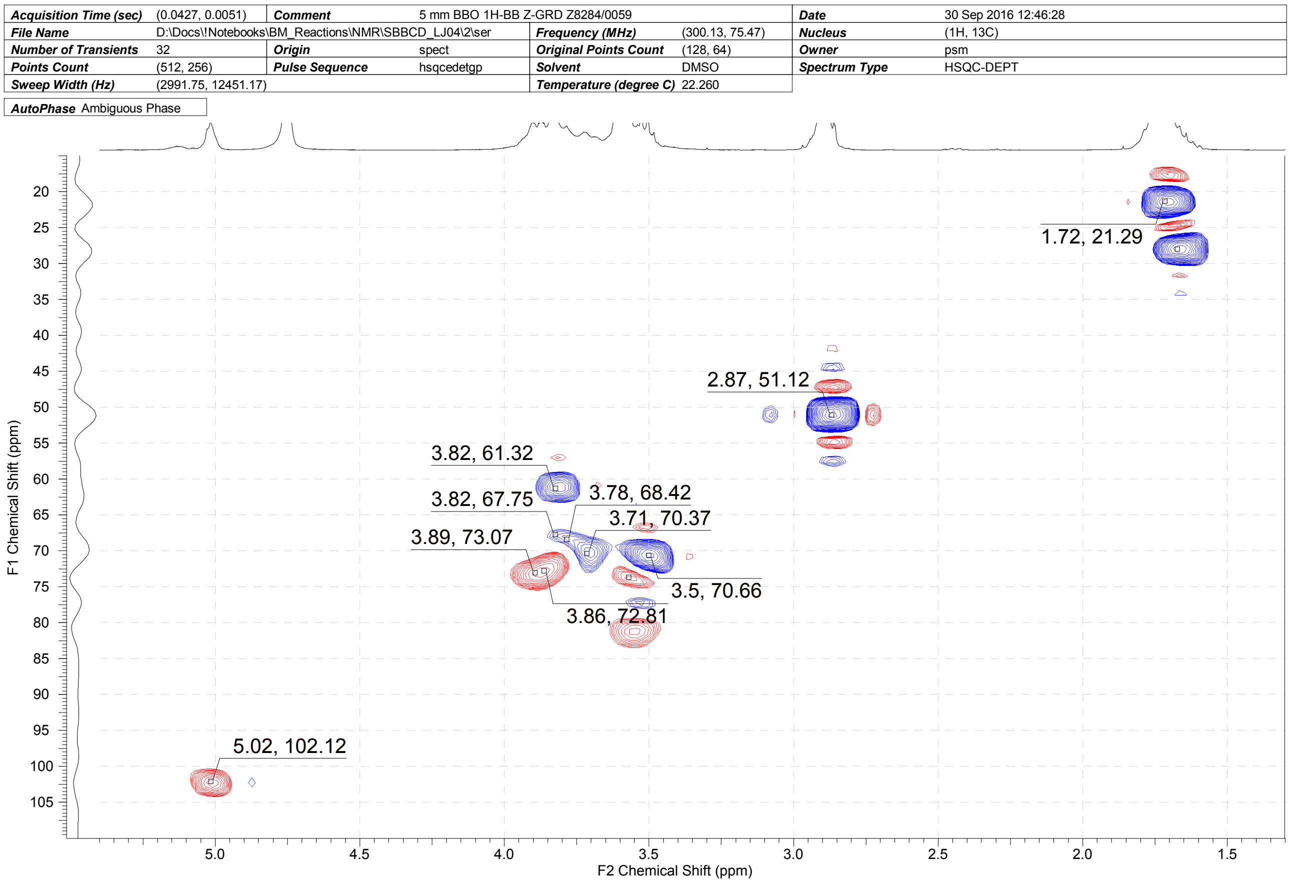

*Figure S 52: HSQC-DEPT spectrum of sulfobutylated βCD (**4'**) prepared in ball mill (item 15 in Table 1), DS ≈7.5-7.8*

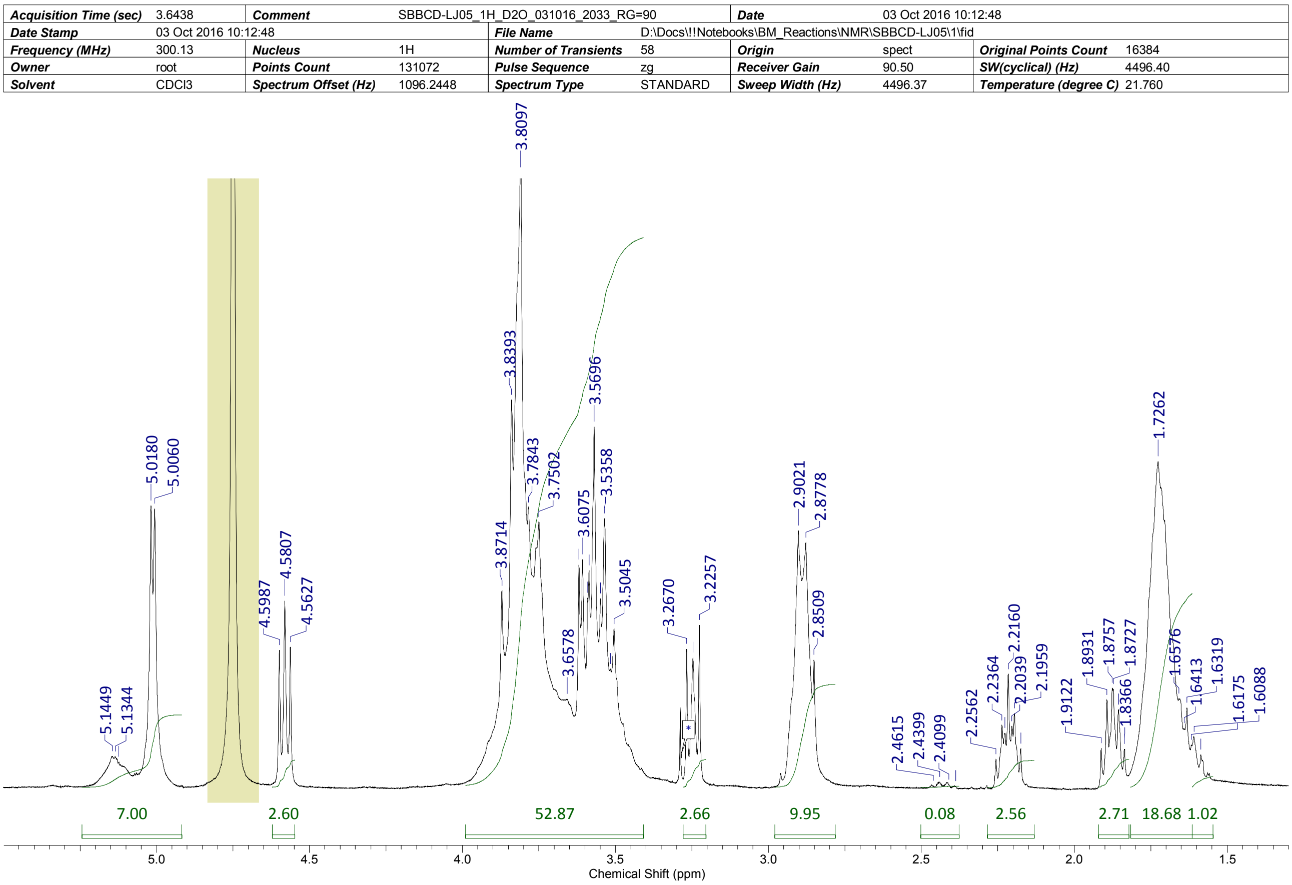

*Figure S 53: Proton NMR spectrum of sulfobutylated βCD (**4’**) prepared in ball mill (item 16 in Table 1), DS ≈4.4-4.9*

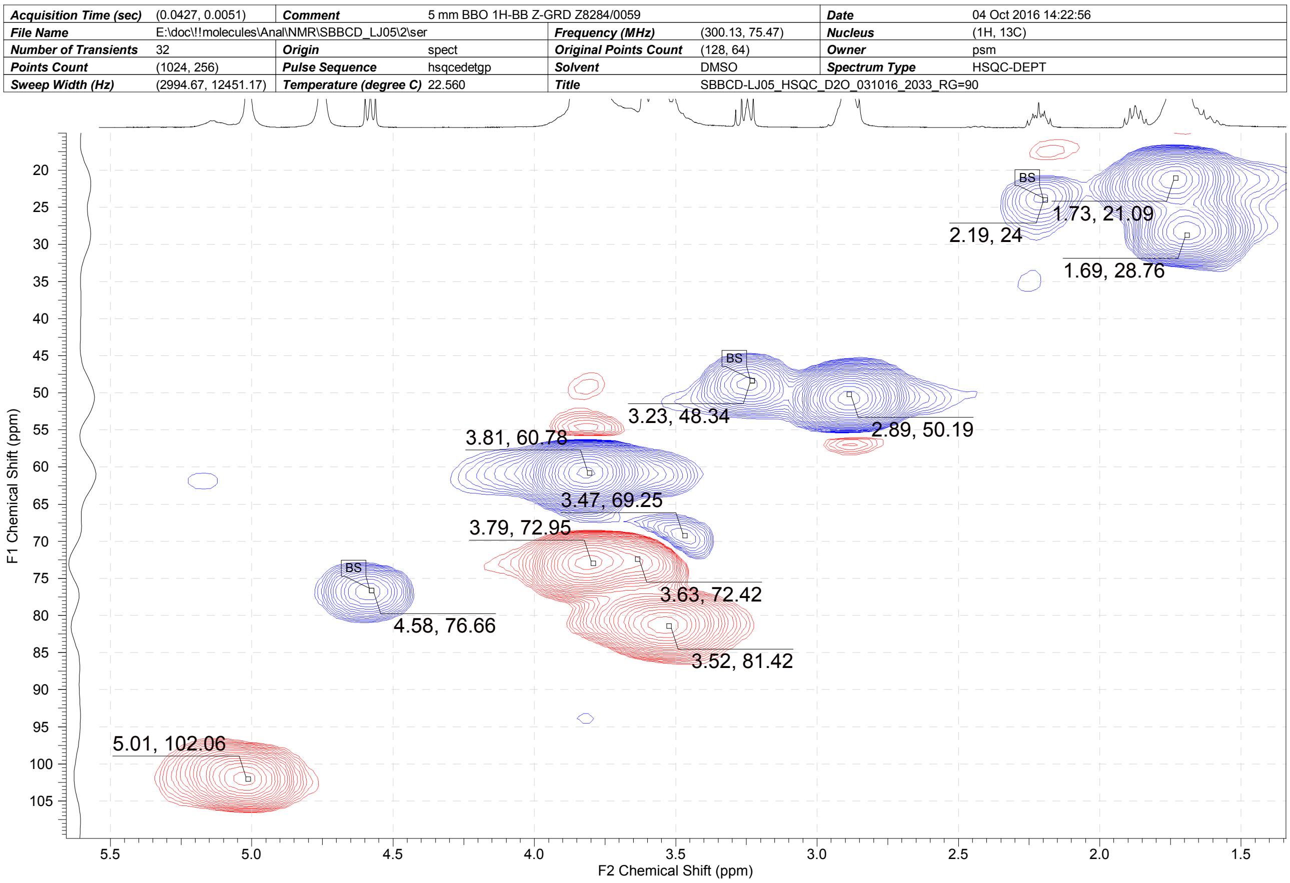

*Figure S 54: HSQC-DEPT spectrum of sulfobutylated βCD (**4’**) prepared in ball mill (item 16 in Table 1), DS ≈4.4-4.9*

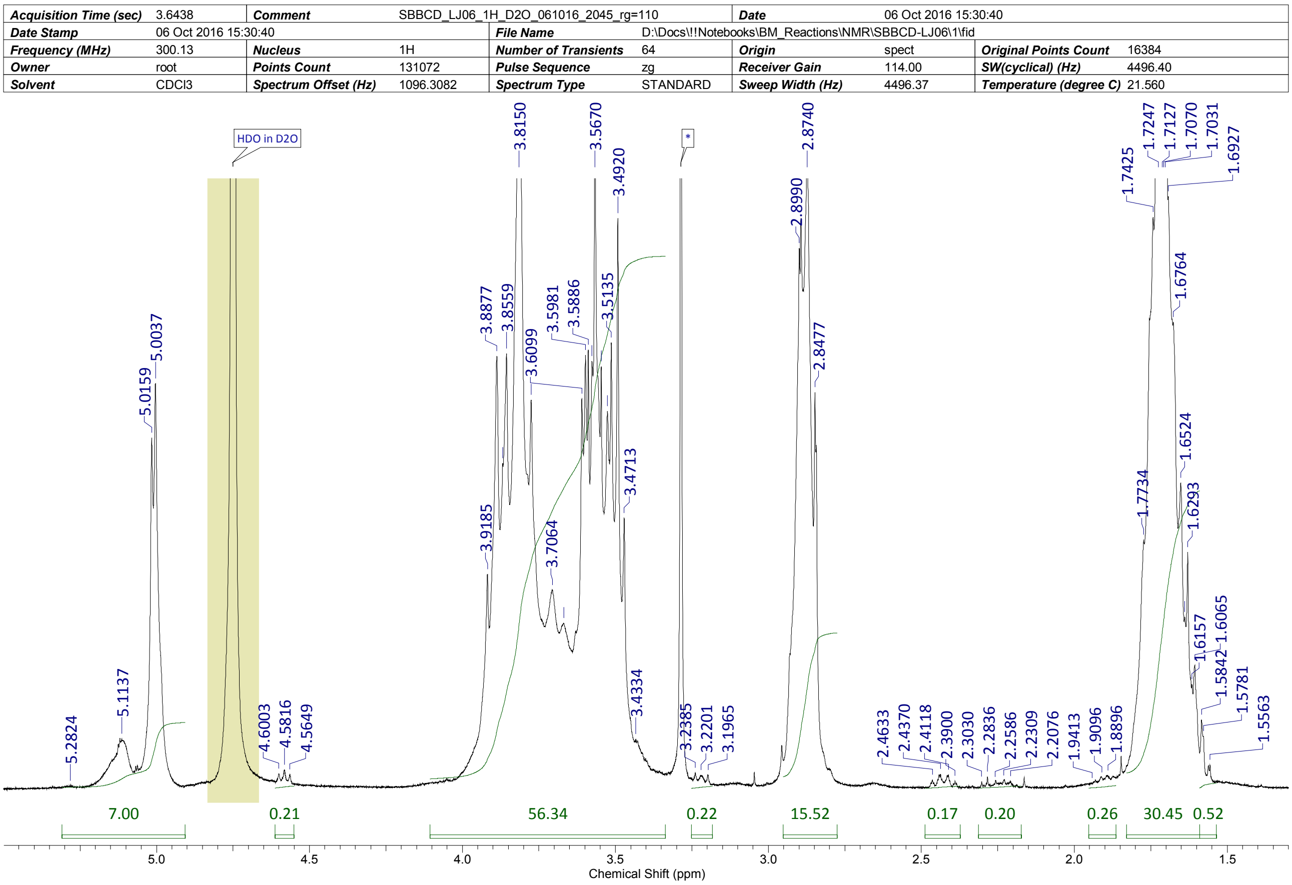

*Figure S 55: Proton NMR spectrum of sulfobutylated ßCD (**4’**) prepared in ball mill (item 17 in Table 1), DS ≈6.9-7.5*

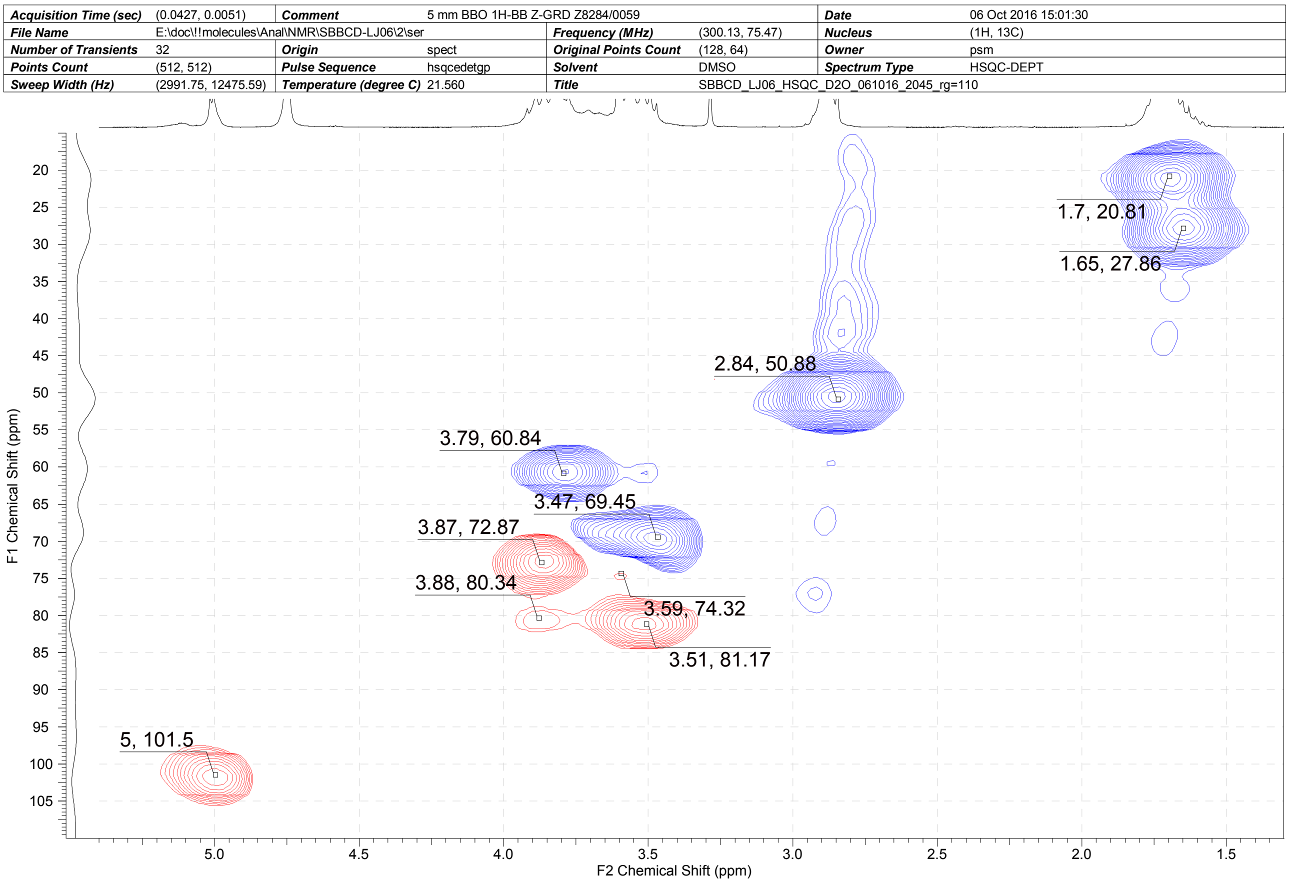

*Figure S 56: HSQC-DEPT spectrum of sulfobutylated ßCD (**4’**) prepared in ball mill (item 17 in Table 1), DS ≈6.9-7.5*

Acquisition Time (sec)	3.6438	***Comment***	HOBSANA_1H_D2O_120117_2122_RG=180 8 mg/0.8 ml	***Date***	13 Jan 2017 08:08:48		
Date Stamp	13 Jan 2017 08:08:48	***File Name***	E:\Documents\!!Molecules\Anal\HOBSANA\1\fid	***Frequency (MHz)***	300.13		
Nucleus	1H	***Number of Transients***	64	***Origin***	spect	***Original Points Count***	16384
Owner	root	***Points Count***	131072	***Pulse Sequence***	zg	***Receiver Gain***	181.00
SW(cyclical) (Hz)	4496.40	***Solvent***	CHLOROFORM-d	***Spectrum Offset (Hz)***	1096.6907	***Spectrum Type***	STANDARD
Sweep Width (Hz)	4496.37	***Temperature (degree C)***	20.260				

No.	Shift1 (ppm)	H's	Type	J (Hz)	Multiplet1	(ppm)
1	1.59	23	dd	6.40, 6.30	beta	[1.54 .. 1.65]
2	1.71	14	dddd	8.00, 7.90	gamma	[1.65 .. 1.78]
3	2.86	24	dd	7.80	delta	[2.81 .. 2.92]
4	3.55	24	dd	6.30	alpha	[3.51 .. 3.61]

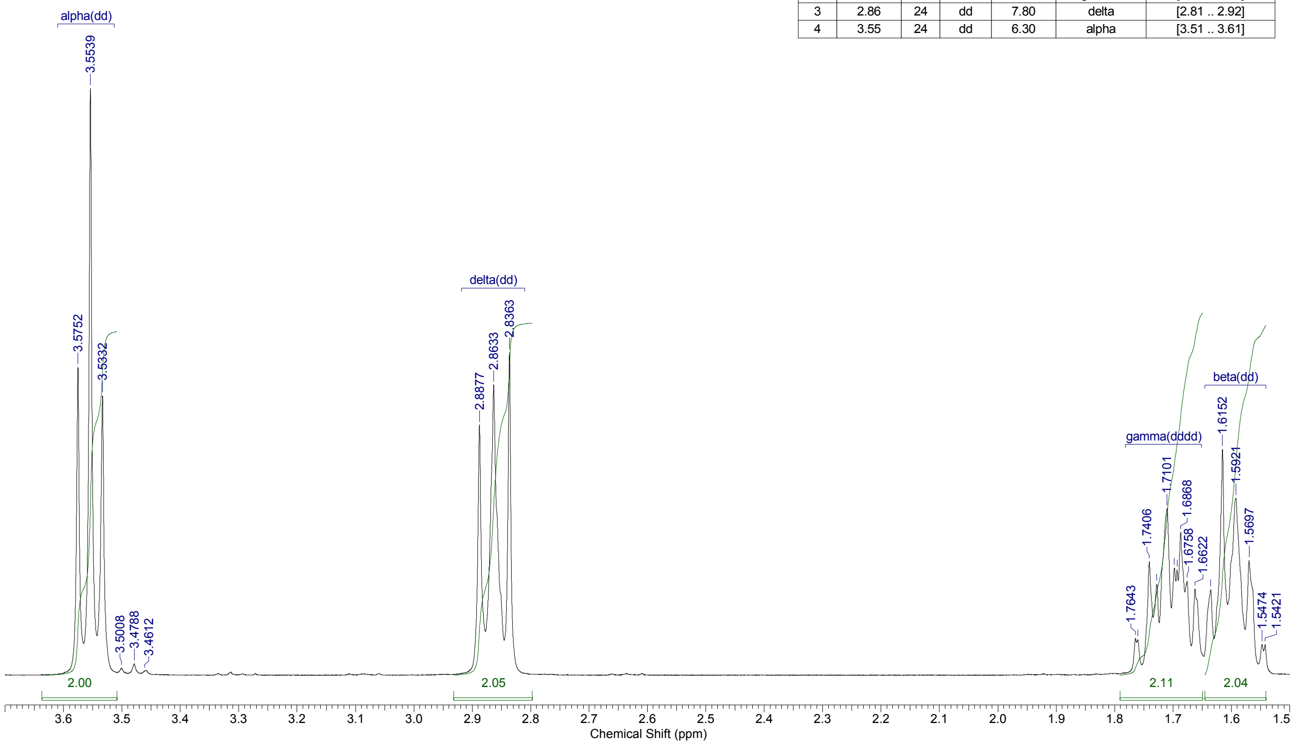

Figure S 57: Proton NMR spectrum of 4-hydroxy-1-butanesulfonic acid sodium salt (HOBSANa)

Acquisition Time (sec)	(0.0427, 0.0051)	***Comment***	5 mm BBO 1H-BB Z-GRD Z8284/0059	***Date***	13 Jan 2017 09:02:26		
File Name	E:\Documents\!!Molecules\Anal\HOBSANA\2\ser	***Frequency (MHz)***	(300.13, 75.47)	***Nucleus***	(1H, 13C)		
Number of Transients	32	***Origin***	spect	***Original Points Count***	(128, 64)	***Owner***	psm
Points Count	(1024, 512)	***Pulse Sequence***	hsqcedetgp	***Solvent***	DMSO	***Spectrum Type***	HSQC-DEPT
Sweep Width (Hz)	(2994.67, 12475.59)	***Temperature (degree C)***	20.360	***Title***	HOBSANA_HSQC_D2O_120117_2122_RG=180 8 mg/0.8 ml		
AutoPhase	Ambiguous Phase						

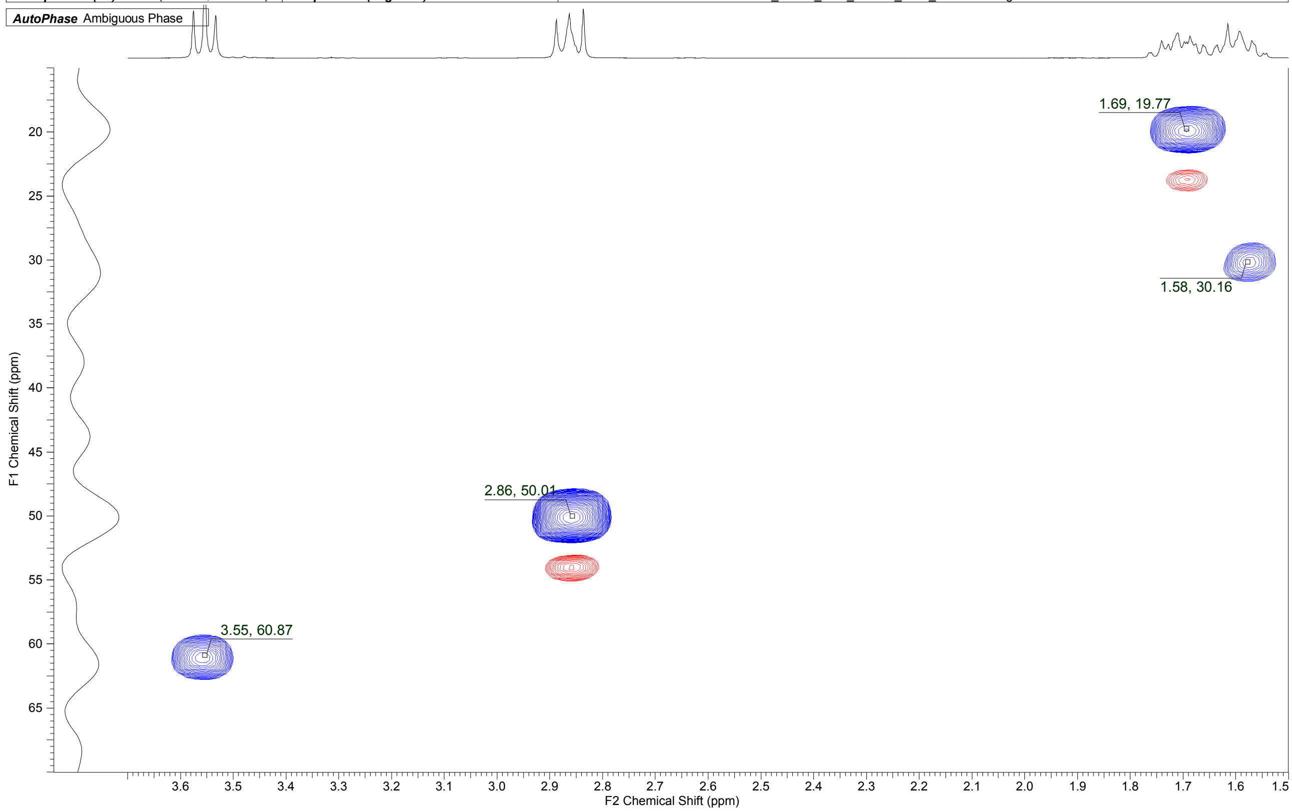

Figure S 58: HSQC-DEPT spectrum of HOBSANa

Acquisition Time (sec)	3.6438	*Comment*	BIBSANA_1H_D2O_130117_2123_RG=180 8 mg/0.8 ml	*Date*	13 Jan 2017 09:14:56				
Date Stamp	13 Jan 2017 09:14:56			*File Name*	E:\Documents\!!Molecules\Anal\BIBSANA\1\fid			*Frequency (MHz)*	300.13
Nucleus	1H	*Number of Transients*	64	*Origin*	spect	*Original Points Count*	16384	*Owner*	root
Points Count	131072	*Pulse Sequence*	zg	*Receiver Gain*	181.00	*SW(cyclical) (Hz)*	4496.40	*Solvent*	D2O
Spectrum Offset (Hz)	1096.5878	*Spectrum Type*	STANDARD	*Sweep Width (Hz)*	4496.37	*Temperature (degree C)*	20.460		

No.	Shift1 (ppm)	H's	Type	J (Hz)	Multiplet1	(ppm)
1	1.62	2	m	-	beta	[1.57 .. 1.67]
2	1.71	2	m	-	gamma	[1.67 .. 1.77]
3	2.86	24	dd	7.80	delta	[2.82 .. 2.91]
4	3.48	24	dd	6.30	alpha	[3.43 .. 3.53]

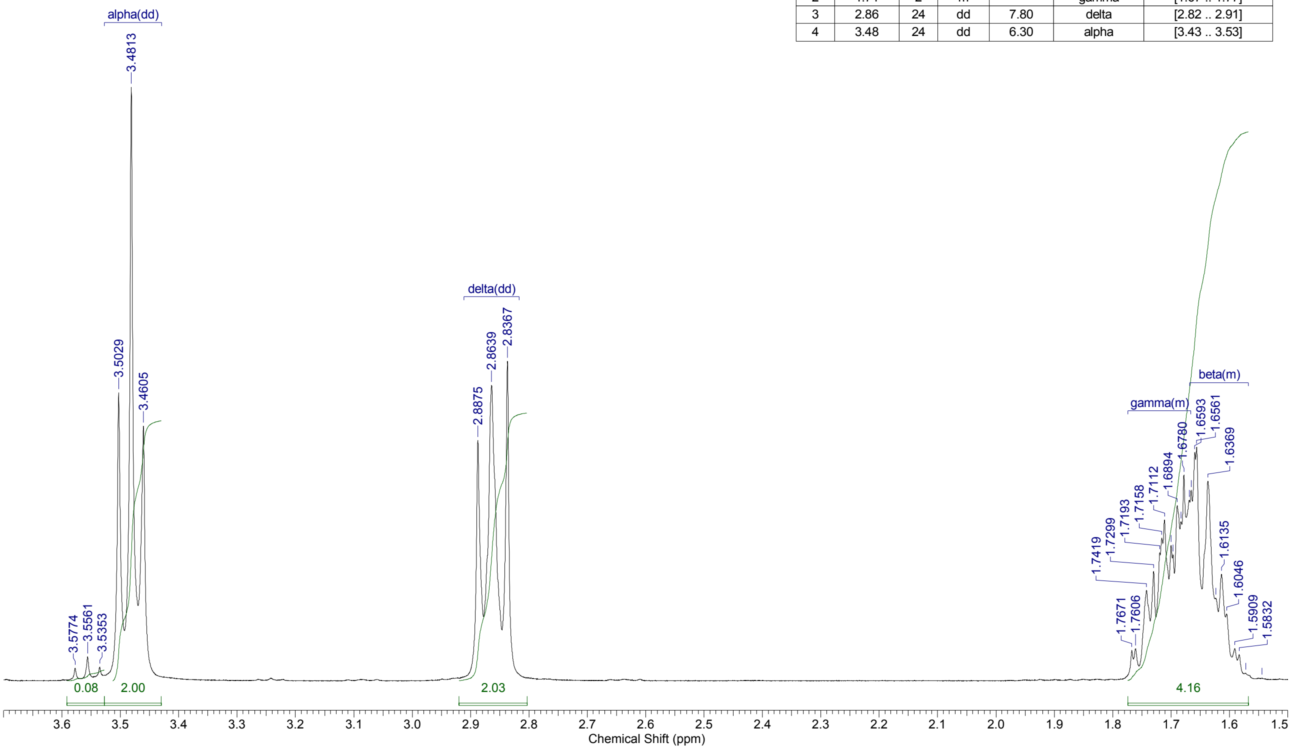

Figure S 59: Proton NMR spectrum of di(1,1'-sulfonatobutyl)ether disodium salt (disodium 4,4'-oxydibutane-1,1'-disulfonate, BIBSANa)

Acquisition Time (sec)	(0.0427, 0.0051)	*Comment*	5 mm BBO 1H-BB Z-GRD Z8284/0059			*Date*	13 Jan 2017 10:10:04
File Name	E:\Documents\!!Molecules\Anal\BIBSANA\2\ser			*Frequency (MHz)*	(300.13, 75.47)	*Nucleus*	(1H, 13C)
Number of Transients	32	*Origin*	spect	*Original Points Count*	(128, 64)	*Owner*	psm
Points Count	(1024, 512)	*Pulse Sequence*	hsqcedetgp	*Solvent*	DMSO	*Spectrum Type*	HSQC-DEPT
Sweep Width (Hz)	(2994.67, 12475.59)	*Temperature (degree C)*	20.560	*Title*	BIBSANA_HSQC_D2O_130117_2123_RG=180 8 mg/0.8 ml		

AutoPhase Ambiguous Phase

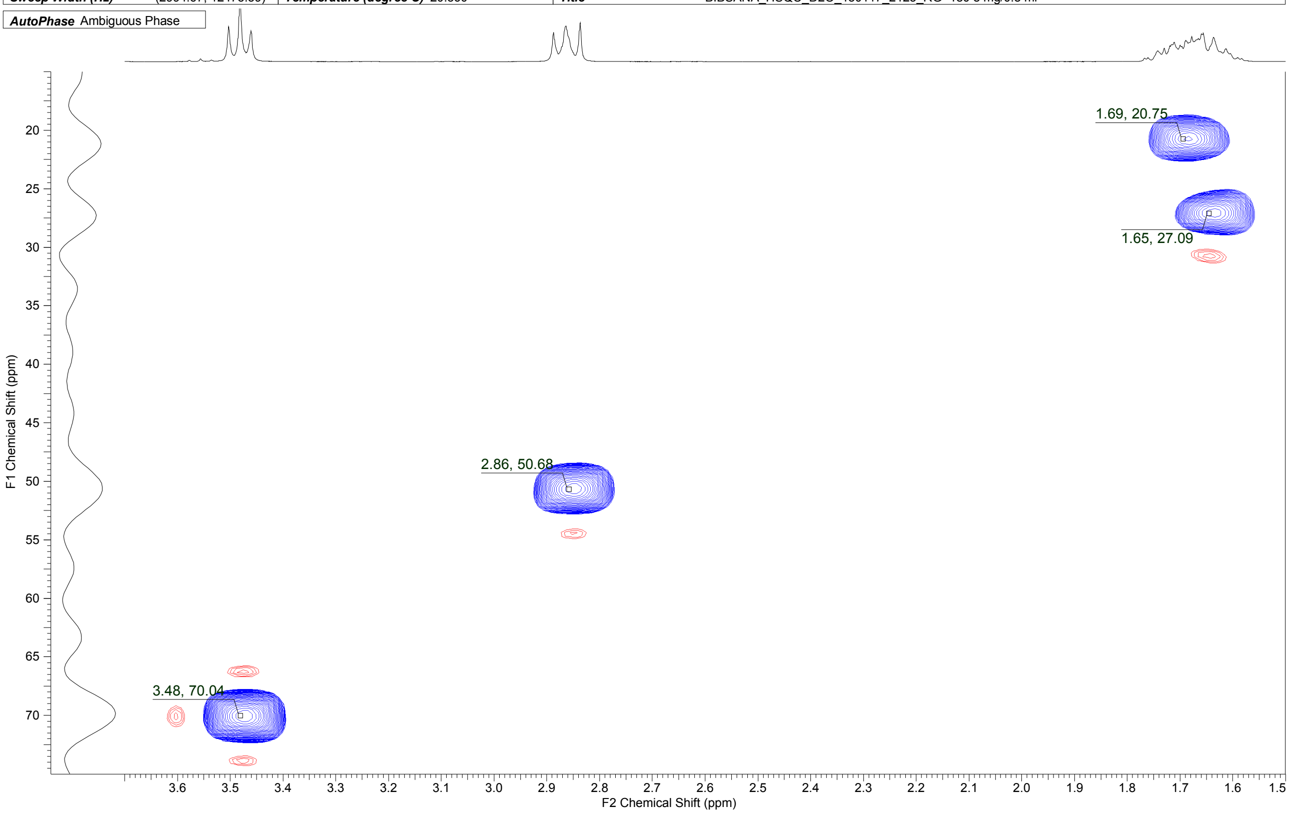

Figure S 60: HSQC-DEPT spectrum of BIBSANa

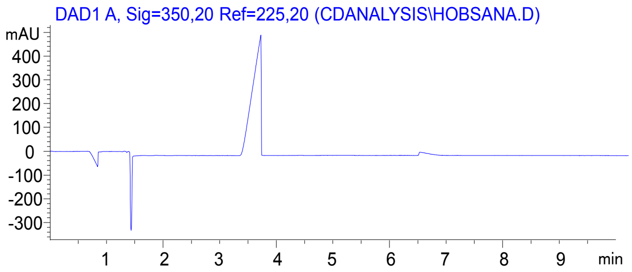

4-hydroxybutanesulfonic acid

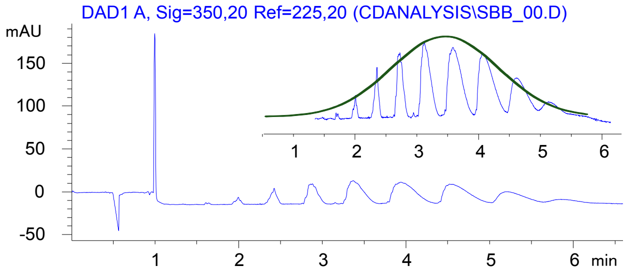

Sulfobutylated ßCD (**4**) DS 6.2-6.5, synthesized in solution(item 11 in Table 1)

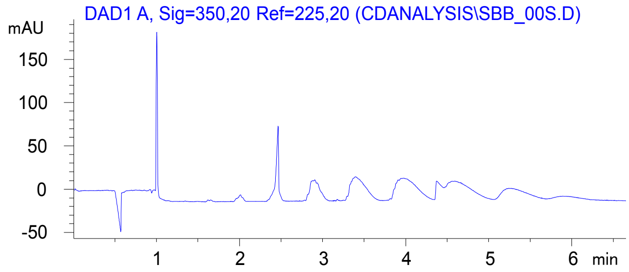

Sulfobutylated ßCD (**4**) DS 6.2-6.5, synthesized in solution(item 11 in Table 1) spiked with 1 % 4-hydroxybutanesulfonic acid

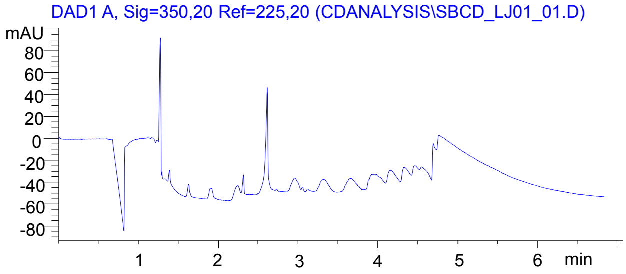

Sulfobutylated ßCD (**4'**) DS 3.1-3.3, synthesis in ball mill (item 12 in Table 1)

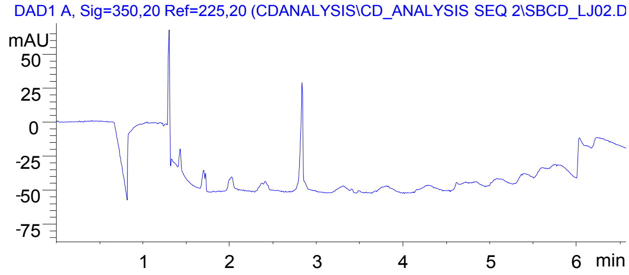

Sulfobutylated ßCD (**4'**) DS 5.2-5.8, synthesis in ball mill (item 13 in Table 1)

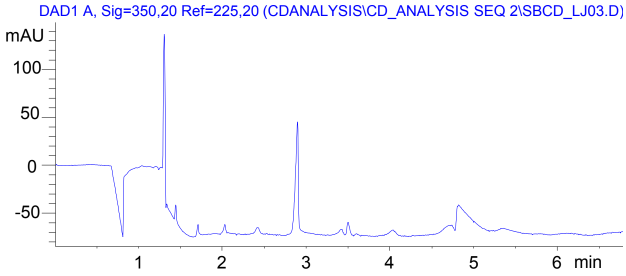

Sulfobutylated ßCD (**4'**) DS 6.2-6.6, synthesis in ball mill (item 14 in Table 1)

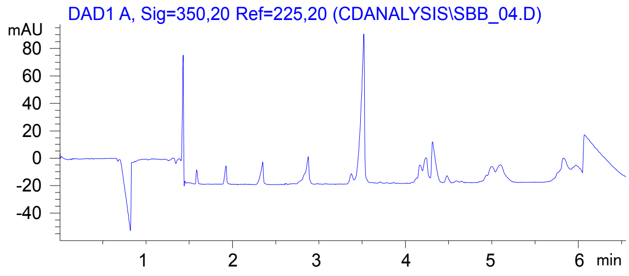

Sulfobutylated ßCD (**4'**) DS 7.5-7.8, synthesis in ball mill (item 15 in Table 1)

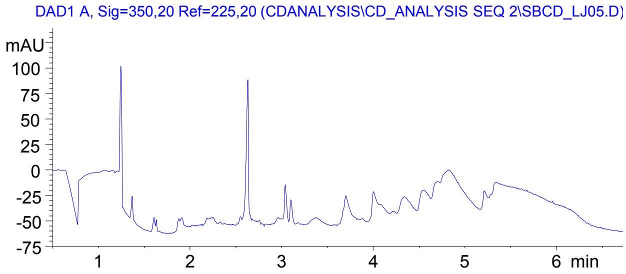

Sulfobutylated ßCD (**4'**) DS 4.4-4.9, synthesis in ball mill (item 16 in Table 1)

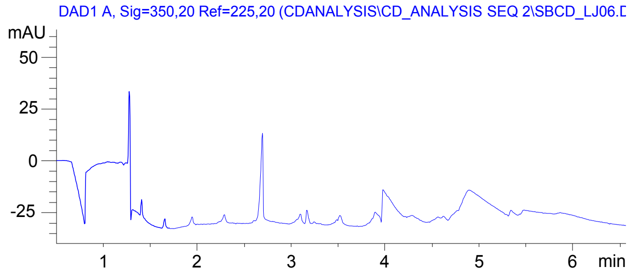

Sulfobutylated ßCD (**4'**) DS 6.9-7.5, synthesis in ball mill (item 17 in Table 1)

Figure S 61: Capillary electropherogram of sulfobutylated ßCDs

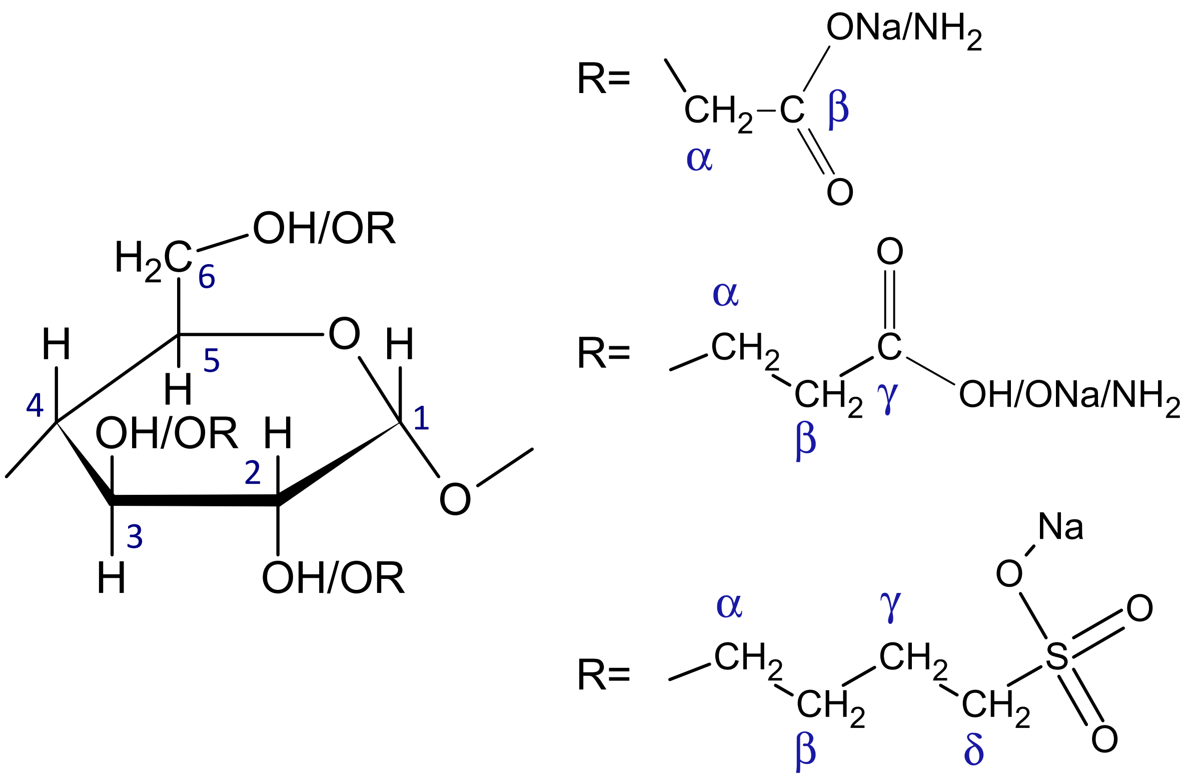

Glucopyranose unit Substituent

Scheme 1

Scheme S 1: Carbon atom numbering for the NMR assignment (OH: unsubstituted; OR: substituted)

Table S 1: Proton assignment of cyclodextrin derivatives prepared in solution(based on ^{1}H- and HSQC-DEPT experiments)

	Compound **2** (CMβCD)[1]	Compound **3** (CEβCD)[2]	Compound **5** (CAMβCD)[3]	Compound **4** (SBβCD)[4]	HOBSANa[5]	BIBSANa[6]
C(1)H [7]	5.00, 5.21	4.99	4.97, 4.99	5.02, 5.11	--	--
C(2)H unsubst	3.54-3.61	3.56-3.68	3.41-3.63	3.48-3.61	--	--
C(2)H subst [8]	3.34-3.63	3.43-3.59	3.38-3.60	3.57-3.67	--	--
C(3)H unsubst [8]	3.84-4.04	3.79-3.96	3.63-3.98	3.70-3.90	--	--
C(3)H subst	--	--	--	3.70-3.83	--	--
C(4)H [8]	3.44-3.62	3.43-3.59	3.38-3.60	3.40-3.57	--	--
C(5)H [8]	3.69-3.88	3.73-3.92	3.63-3.98	3.76-3.90	--	--
C(6)H2 unsubst	3.70-3.91	3.70-3.89	3.61-3.93	3.71-3.90	--	--
C(6)H2 subst	--	3.72-3.81(?)	--	3.65-3.73	--	--
alpha to -O	3.86-3.95; 4.03-4.21	3.62-3.74	3.61-3.77	3.43-3.54	3.55	3.48
beta to -O	--	2.35-2.46	2.38-2.51	1.67	1.58	1.65
gamma to -O	--	--	--	1.71	1.69	1.69
delta to -O	--	--	--	2.86	2.86	2.86

[1] CM= Carboxymethyl; [2] CE=Carboxyethyl; [3] CAM=Carbamoylethyl; [4] SB=Sulfobutyl; [5] HOBSANa=4-hydroxybutanesulfonic acid sodium salt; [6] BIBSANa: di(1,1'-sulfonatolbutyl)ether disodium salt (disodium 4,4'-oxydibutane-1,1'-disulfonate); [7] main peaks only; [8] shaded assignments may be altered

Table S 2: Carbon assignment of cyclodextrin derivatives prepared in solution (based on ^{13}C- and HSQC-DEPT experiments)

	Compound **2** (CMβCD)[1]	Compound **3** (CEβCD)[2]	Compound **5** (CAMβCD)[3]	Compound **4** (SBβCD)[4]	HOBSANa[5]	BIBSANa[6]
C1 [7]	100.3, 102.0	102.1	103.1	99.2, 101.1	--	--
C2 unsubst	72.6	72.0	71.9	72.5	--	--
C2 subst [8]	81.1	80.7	81.4	78.3-80.4	--	--
C3 unsubst [7,8]	72.2, 73.8	73.3, 71.1	72.4	70.6	--	--
C3 subst	--	--	--	80.6	--	--
C4 [8]	81.4	81.5	81.4	80.5	--	--
C5 [8]	71.8	72.3	72.7	70.6	--	--
C6 unsubst	61.2	60.4	60.1	60.8	--	--
C6 subst	--	--	--	71.2	--	--
alpha to CD-O [8]	69.7, 70.4	68.6	66.3	70.2	60.9	70.0
beta to CD-O [8]	177.9	38.1	33.4	27.9	30.2	27.1
gamma to CD-O	--	180.7	177.4 (174.3)[9]	21.0	19.8	20.8
delta to CD-O	--	--	--	50.2	50.0	50.7

[1] CM= Carboxymethyl; [2] CE=Carboxyethyl; [3] CAM=Carbamoylethyl; [4] SB=Sulfobutyl; [5] HOBSANa=4-hydroxybutanesulfonic acid sodium salt; [6] BIBSANa: di(1,1'-sulfonatobutyl)ether disodium salt (disodium 4,4'-oxydibutane-1,1'-disulfonate); [7] main peaks only; [8] shaded assignments may be altered; [9] protonated carboxyl group (tentative) of the partially hydrolyzed product

Table S 3: Characteristic C=O IR bands of the prepared (carbonyl)ethyl group containing CD derivatives

	Compound **3** (CEβCD)	Compound **5** (CAMβCD) *Item 9*	Compound **5** (CAMβCD) *Item 10*
C=O as COO^-	1570	1559	--
C=O as COOH	1726	--	--
C=O as $CONH_2$	--	1667	1664

Table S 4: R_F-values of the prepared compounds after twice run of the same plate in the same solvent mixture at 7 cm distance in 100 µg

βCD	0.53-0.54					
CMβCD [1]	0.05-0.48	strip with multiple spots	0.45-0.48	mono		
CEβCD [2]	0.15-0.47	strip with multiple spots	0.44-0.47	mono		
CAMβCD [3]	0.54-0.65	strip with multiple spots	0.55-0.57	mono	0.21-0.54	light brown strip no distinctive spots
SBβCD [4]	0.05-0.43	strip with multiple spots	0.40-0.43	mono		

[1] CM= Carboxymethyl; [2] CE=Carboxyethyl; [3] CAM=Carbamoylethyl; [4] SB=Sulfobutyl

References

[1] Strohalm, M.; Kavan, D.; Novák, P.; Volný, M. & Havlíček, V., mMass 3: A Cross-Platform Software Environment for Precise Analysis of Mass Spectrometric Data. *Analytical Chemistry,* **2010**, *82*, 4648-4651

[2] mMass v5.5, An Open Source Mass Spectrometry Tool, www.mmass.org
